# Targeting heterogeneous tumor microenvironments in pancreatic cancer mouse models of metastasis by TGF-**β** depletion

**DOI:** 10.1172/jci.insight.182766

**Published:** 2024-11-08

**Authors:** Sophia Y. Chen, Heng-Chung Kung, Birginia Espinoza, India Washington, Kai Chen, Jianxin Wang, Haley Zlomke, Michael Loycano, Rulin Wang, Michael Pickup, William R. Burns, Juan Fu, William L. Hwang, Lei Zheng

**Affiliations:** 1 Department of Oncology and the Sidney Kimmel Comprehensive Cancer Center,; 2 Pancreatic Cancer Precision Medicine Center of Excellence Program, and; 3 Department of Surgery, The Johns Hopkins University School of Medicine, Baltimore, Maryland, USA.; 4 Bristol Myers Squibb, Princeton, New Jersey, USA.; 5 Center for Systems Biology, Department of Radiation Oncology, Center for Cancer Research, Massachusetts General Hospital, Harvard Medical School, Boston, Massachusetts, USA.; 6 Broad Institute of MIT and Harvard, Cambridge, Massachusetts, USA.

**Keywords:** Immunology, Oncology, Cancer immunotherapy

## Abstract

The dual tumor-suppressive and -promoting functions of TGF-β signaling has made its targeting challenging. We examined the effects of TGF-β depletion by AVID200/BMS-986416 (TGF-β-TRAP), a TGF-β ligand trap, on the tumor microenvironment of pancreatic ductal adenocarcinoma (PDAC) murine models with different organ-specific metastasis. Our study demonstrated that TGF-β-TRAP potentiates the efficacy of anti–programmed cell death 1 (anti–PD-1) in a PDAC orthotopic murine model with liver metastasis tropism, significantly reducing liver metastases. We further demonstrated the heterogeneous response of cytotoxic effector T cells to combination TGF-β-TRAP and anti–PD-1 treatment across several tumor models. Single-nuclear RNA sequencing suggested that TGF-β-TRAP modulates cancer-associated fibroblast (CAF) heterogeneity and suppresses neutrophil degranulation and CD4^+^ T cell response to neutrophil degranulation. Ligand-receptor analysis indicated that TGF-β-TRAP may modulate the CCL5/CCR5 axis as well as costimulatory and checkpoint signaling from CAFs and myeloid cells. Notably, the most highly expressed ligands of CCR5 shifted from the immunosuppressive CCL5 to CCL7 and CCL8, which may mediate the immune agonist activity of CCR5 following TGF-β-TRAP and anti–PD-1 combination treatment. This study suggested that TGF-β depletion modulates CAF heterogeneity and potentially reprograms CAFs and myeloid cells into antitumor immune agonists in PDAC, supporting the validation of such effects in human specimens.

## Abstract 2

TGFB depletion by AVID200 enhances anti-PD-1 therapy and reprograms cancer-associated fibroblasts and myeloid cells within the tumor microenvironment of a murine model with liver-metastasis tropism.

## Graphical abstract

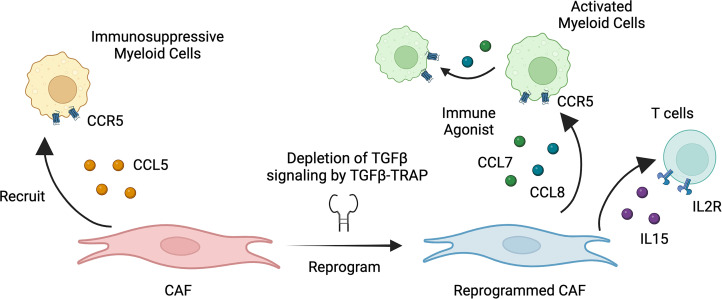

## Introduction

Though immune checkpoint inhibitors (ICIs) have revolutionized the treatment paradigm of various cancers, it has found limited success with pancreatic ductal adenocarcinoma (PDAC). The resistance to ICIs in PDAC has been primarily attributed to its immune-quiescent tumor microenvironment (TME) and dense stroma (1). Given these challenges, identifying agents that target the PDAC stroma is an attractive potential therapeutic strategy.

The transforming growth factor β (TGF-β) signaling pathway regulates many key physiologic processes, and dysregulation of this pathway has been associated with various diseases, including cancer. Depending on the cellular context and surrounding environment, TGF-β, a multifunctional cytokine, can act as a tumor suppressor or a tumor promoter (2). In normal tissues and preinvasive dysplasia, TGF-β displays tumor-suppressive activity by inducing cell cycle arrest, regulating cell differentiation, and promoting apoptosis. In invasive cancers, TGF-β can promote tumor progression, epithelial-to-mesenchymal transition (EMT), invasion, metastasis, angiogenesis, and immune escape (3–5). Thus, the dual function and pleiotropic nature of TGF-β signaling has made TGF-β a challenging target. Nevertheless, targeting TGF-β in established cancers such as PDAC, which are highly aggressive and metastatic, remains of significant interest despite the challenges.

Existing strategies to block TGF-β signaling include receptor tyrosine kinase inhibitors, monoclonal neutralizing antibodies that prevent TGF-β binding to receptor complexes, antisense oligonucleotides, and TGF-β–related vaccines (6–8). However, efficacy is limited by both isoform specificity and adverse effects from lack of target selectivity. To address these shortcomings, TGF-β ligand traps composed of Fc-stabilized dimers of TGF-βII receptor extracellular domains were developed to sequester TGF-β1 and TGF-β3 (not TGF-β2) to prevent binding to TGF-βII receptors (6, 9). Given that TGF-β2 is a positive regulator of hematopoiesis and normal cardiac function, the advantages of selectively targeting TGF-β1/3 may make TGF-β ligand traps better tolerated as oncologic therapy. Importantly, the TGF-βII receptor extracellular domains on the trap molecules have a high affinity for TGF-β1/3 and are thus anticipated to completely neutralize these TGF-β ligands.

Although multiple TGF-β ligand traps (10) have been developed, their immune mechanisms of action have not been fully elucidated. Genetic depletion of TGF-β to study the immune mechanisms of TGF-β is not feasible as the germline null allele of a single TGF-β ligand isoform causes embryonic lethality in mice (11). As such, TGF-β ligand traps serve as effective tools for studying the immunomodulatory effects of TGF-β in PDAC by neutralizing TGF-β ligands. AVID200 (BMS-986416/TGF-β-TRAP) is one such TGF-β ligand trap with a reported binding affinity up to 1,000-fold higher than other trap agents (12–14). Several studies have reported the benefits of AVID200 for the treatment of hematologic disorders such as myelofibrosis (15, 16) and Fanconi anemia (17). Though studies on the effect of AVID200 on solid tumors have been limited, AVID200 monotherapy was well tolerated and effectively modulated TGF-β1/3 in a phase I study (NCT03834662) of 19 patients with advanced or metastatic solid tumor malignancies (18, 19). These promising findings warrant further investigation of AVID200 as a potential agent for oncologic therapy in combination with other anticancer therapies including ICIs. Nevertheless, how TGF-β traps modulate TME components such as cancer-associated fibroblasts (CAFs) and myeloid cells in PDAC has not been investigated in depth, with studies limited to effects on T cells and target molecule expression on myeloid cells (20).

In many cancers including PDAC, CAFs have been found to play major roles in cancer progression, including secreting multiple cytokines and growth factors to suppress the immune response and promote cancer cell growth and metastasis (21–23). Previous studies have demonstrated heterogeneity within CAFs, with 3 subpopulations of CAFs having been identified in PDAC: myofibroblastic CAFs (myCAFs), inflammatory CAFs (iCAFs), and antigen-presenting CAFs (24, 25). The complex interplay between CAFs and TGF-β has also been reported in several studies. CAF-secreted TGF-β has been shown to promote EMT, drive myCAF differentiation, and antagonize IL-1–induced JAK/STAT signaling necessary for iCAF formation (26, 27). Furthermore, a subpopulation of TGF-β–driven myCAFs that express leucine-rich repeat containing 15 (LRRC15) has been associated with poor response to anti–programmed cell death 1/ligand 1 (anti–PD-1/L1) therapy in several clinical trials (28). Selective depletion of LRRC15^+^ CAFs reactivated tumor-infiltrating CD8^+^ T cells and enhanced responsiveness to ICIs in a murine PDAC model (29).

TGF-β is also a key factor driving the differentiation and reprogramming of myeloid cells, particularly pro-tumor M2 polarized macrophages and myeloid-derived suppressive cells (MDSCs). Our group found that interaction between integrin αV/β8 on cancer cells and GARP on M1-like macrophages resulted in the activation of TGF-β signaling and reprogramming of M1-like macrophages to M2-like (30). Crucially, M2-like macrophages and MDSCs are also potent sources of TGF-β, further enhancing the pro-tumor effects of TGF-β within the TME. However, the interplay between TGF-β–mediated CAF differentiation and myeloid cell reprogramming has not been studied.

Previously, we found that murine PDAC cell lines with organ-specific metastatic tropism are associated with differences in the methylation of CAF metabolic genes and overall CAF heterogeneity (31). This prior study provides an in vivo PDAC model to investigate how depletion of TGF-β by TGF-β traps may modulate the interaction between heterogenous CAF populations and myeloid cells and influence treatment response to TGF-β depletion and ICIs.

## Results

### TGF-β-TRAP potentiates anti–PD-1 treatment response in a PDAC orthotopic murine model with metastatic liver tropism.

**Figure 1:**
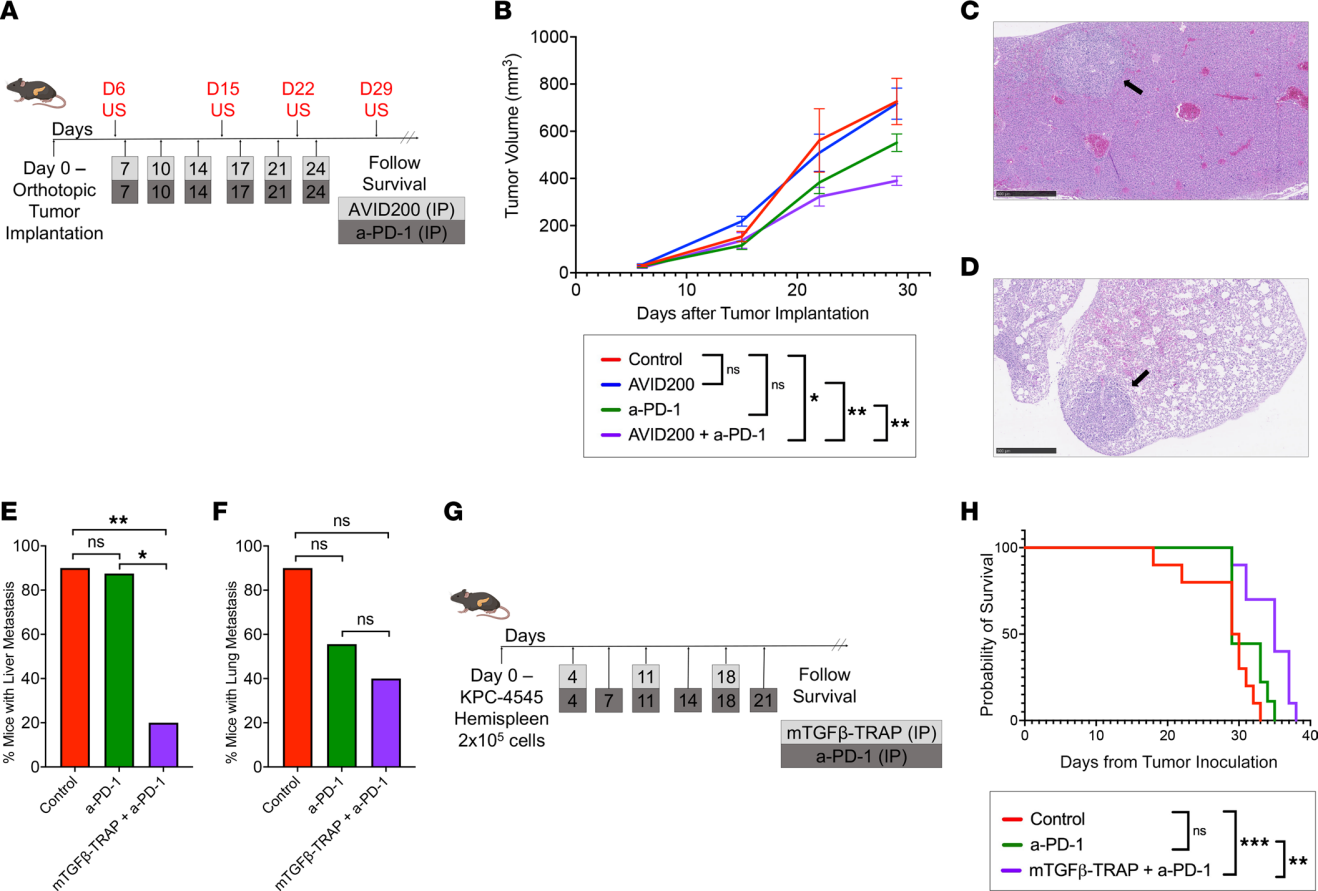
The combination of TGF-β-TRAP and a-PD-1 slows the rate of tumor growth and prolongs survival in PDAC mouse models with KPC-4545 cell line. (**A**) Treatment schema for PDAC orthotopic model tumor growth experiment using AVID200, the human surrogate form of TGF-β-TRAP. Pretreatment US was performed on day 6. Mice were treated with AVID200 (5 mg/kg i.p. twice/week), a-PD-1 (5 mg/kg i.p. twice/week), or IgG control (5 mg/kg i.p. twice/week) for 3 weeks. (**B**) PDAC orthotopic tumor size was evaluated by US weekly until day 29 in mice treated with different combinations of AVID200 and a-PD-1 (*n* = 5 mice per group). Data represent mean ±SEM. (**C** and **D**) Representative H&E images of liver (**C**) and lung metastatic (**D**) sites in PDAC orthotopic mouse model syngeneic wild-type C57BL/6 mice. Original magnification ×5. (**E** and **F**) When PDAC orthotopic tumor mice reached survival endpoint, necropsies were performed, and the number of mice with liver metastases for the KPC-4545 model (**E**) and lung metastases for the KPC-3403 model (**F**) were identified grossly and histologically (*n* = 8–10 mice per group). (**G**) Treatment schema for PDAC hemispleen model using mTGF-β-TRAP, the mouse surrogate form of TGF-β-TRAP. Mice were treated with mTGF-β-TRAP (15 mg/kg i.p. once/week), a-PD-1 (5 mg/kg i.p. twice/week), or IgG control (5 mg/kg i.p. twice/week) for 3 weeks. (**H**) Kaplan-Meier survival curves of PDAC hemispleen model mice treated with different combinations of mTGF-β-TRAP and a-PD-1 (*n* = 10 mice per group). US, ultrasound. Unpaired *t* test was used to compare day 29 tumor volumes. Log-rank test was used for survival analysis. A χ^2^ test was used to examine the correlation between treatment groups and metastasis rates. *, *P* < 0.05; **, *P* < 0.01; ***, *P* < 0.001.

To examine the impact of the combination of TGF-β-TRAP and anti–PD-1 antibody (a-PD-1) on primary tumor growth, we utilized a previously established KPC orthotopic murine model of PDAC (32). We used 2 KPC cell lines: KPC-4545 tumor cell line derived from the primary tumor of a KPC mouse with liver metastases only and KPC-3403 tumor cell line derived from the primary tumor of a KPC mouse with lung metastases only, as previously described (31). We inoculated KPC-4545 tumors orthotopically into mice on day 0 and divided the mice into 4 treatment groups: 1) isotype control (mIgG1, hIgG1f), 2) AVID200 (human TGF-β-TRAP), 3) a-PD-1, and 4) AVID200+a-PD-1 (*n* = 5 mice per group). Tumor volume measurements were obtained by ultrasound (US) once a week, and treatment was administered intraperitoneally as documented in Figure 1A. Compared with the isotype control, a-PD-1 significantly reduced tumor growth while AVID200 had no significant impact on tumor growth in the KPC-4545 model. Of note, after 29 days, the combination of AVID200+a-PD-1 significantly reduced tumor size compared with a-PD-1 monotherapy (Figure 1B), suggesting that TGF-β-TRAP potentiates the antitumor response to a-PD-1 in this orthotopic PDAC model with metastatic liver tropism. Single-agent AVID200 also did not have a notable effect on tumor cell proliferation as measured by Ki-67 staining in both the KPC-4545 and KPC-3403 model (Supplemental Figure 1D; supplemental material available online with this article; https://doi.org/10.1172/jci.insight.182766DS1). This result suggests that the tumor suppression observed in the a-PD-1+AVID200 treatment group was not due to a tumor-intrinsic effect of TGF-β blockade.

Next, we assessed the impact of the combination of TGF-β-TRAP+a-PD-1 on primary tumor growth with KPC-3403 using a similar treatment schema as described previously (Figure 1A). Although at day 29, the AVID200+a-PD-1 combination resulted in a significantly decreased tumor size compared with isotype control and AVID200 alone, the combination was comparable to a-PD-1 monotherapy (Supplemental Figure 1A). These findings suggest that the response to TGF-β-TRAP is different in the PDAC orthotopic murine model with lung metastasis potential compared to the liver metastasis potential model with TGF-β-TRAP potentiating the anti-tumor effects of a-PD-1 in the liver metastasis potential model only.

As anticipated, no lung metastasis was observed in the KPC-4545 tumor model, and no liver metastasis was observed in the KPC-3403 tumor model. A trend toward decreased liver metastasis rate in KPC-4545 tumors was observed with AVID200+a-PD-1 (Supplemental Figure 1B). No decrease in liver metastasis rate was observed in mice treated with AVID200 or a-PD-1 alone in the KPC-4545 tumor model (Supplemental Figure 1B). Similarly, there was a trend toward decreased lung metastasis rate of KPC-3403 tumors treated with AVID200+a-PD-1; however, AVID200 alone did not affect the lung metastasis rate (Supplemental Figure 1C).

### TGF-β-TRAP suppresses liver metastasis formation in PDAC orthotopic murine model with metastatic liver tropism.

We next assessed the incidences of liver and lung metastasis in these 2 orthotopic murine models of PDAC using a treatment schema described in Supplemental Figure 2. mTGF-β-TRAP, the mouse surrogate form of TGF-β-TRAP, was used to replace AVID200 because of concerns of mouse anti-human reaction against AVID200 and was found to recapitulate the antitumor activity of AVID200 in the hemispleen liver metastasis model. To focus on examining metastasis formation in this experiment, orthotopic tumors implanted were approximately 5–7 mm^3^ and were thus larger than those in Figure 1B. As anticipated, the mice quickly reached the survival endpoint and, thus, did not demonstrate treatment effects on the primary pancreatic tumors. Therefore, tumor volumes at necropsy were comparable among all treatment groups in both KPC-4545 (Supplemental Figure 3A) and KPC-3403 (Supplemental Figure 3B) orthotopic tumor models, allowing us to assess the differences in incidences of liver (Figure 1C) and lung (Figure 1D) metastasis in these orthotopic murine models. Since both single-agent a-PD-1 and combination a-PD-1+AVID200, but not single-agent AVID200, demonstrated antitumor efficacy against the KPC-4545 model (Figure 1B), we sought to further differentiate between single-agent a-PD-1 and combination groups. As such, we focused on these 2 groups and excluded the single-agent mTGF-β-TRAP group in this experiment (Figure 1, E and F). In the KPC-4545 model, we observed that mice treated with the combination of mTGF-β-TRAP+a-PD-1 had a significantly decreased incidence of liver metastasis compared with control and a-PD-1 alone (Figure 1E). There were no differences in liver metastasis between control and a-PD-1 monotherapy. Lung metastasis remained undetectable in all treatment groups for the KPC-4545 model. In contrast, in the KPC-3403 orthotopic model, we did not observe the effect of combination mTGF-β-TRAP+a-PD-1 therapy on the incidence of lung metastasis compared with a-PD-1 alone (Figure 1F). Interestingly, a decreased trend in the incidence of lung metastasis was again observed in the KPC-3403 mice treated with a-PD-1 compared with control (Figure 1F). These results suggest that TGF-β-TRAP+a-PD-1 has a more specific effect on the KPC-4545 model and influence on liver metastasis formation.

The above experiment suggested that TGF-β-TRAP suppresses liver metastasis formation in combination with a-PD-1. Following our experiment above, we thus examined the antitumor effect of TGF-β-TRAP+a-PD-1 combination therapy compared with control and single-agent a-PD-1 treatment on liver metastasis directly using our hemispleen liver metastasis model (Figure 1G and Supplemental Figure 1E). The hemispleen model establishes liver metastases by hemispleen injection of KPC-4545 cells (33). The combination of mTGF-β-TRAP+a-PD-1 demonstrated a statistically significant improvement in survival compared with control (*P* = 0.0006) and a-PD-1 monotherapy (*P* = 0.0071) (Figure 1H). These results further support the therapeutic role of mTGF-β-TRAP+a-PD-1 combination therapy in PDAC liver metastases. These results highlight the phenotypically distinct metastatic properties and TGF-β therapy response of 2 PDAC cell lines derived from the same genetically engineered mouse model.

### TGF-β-TRAP reverses a-PD-1–induced T cell exhaustion in PDAC orthotopic tumors.

**Figure 2:**
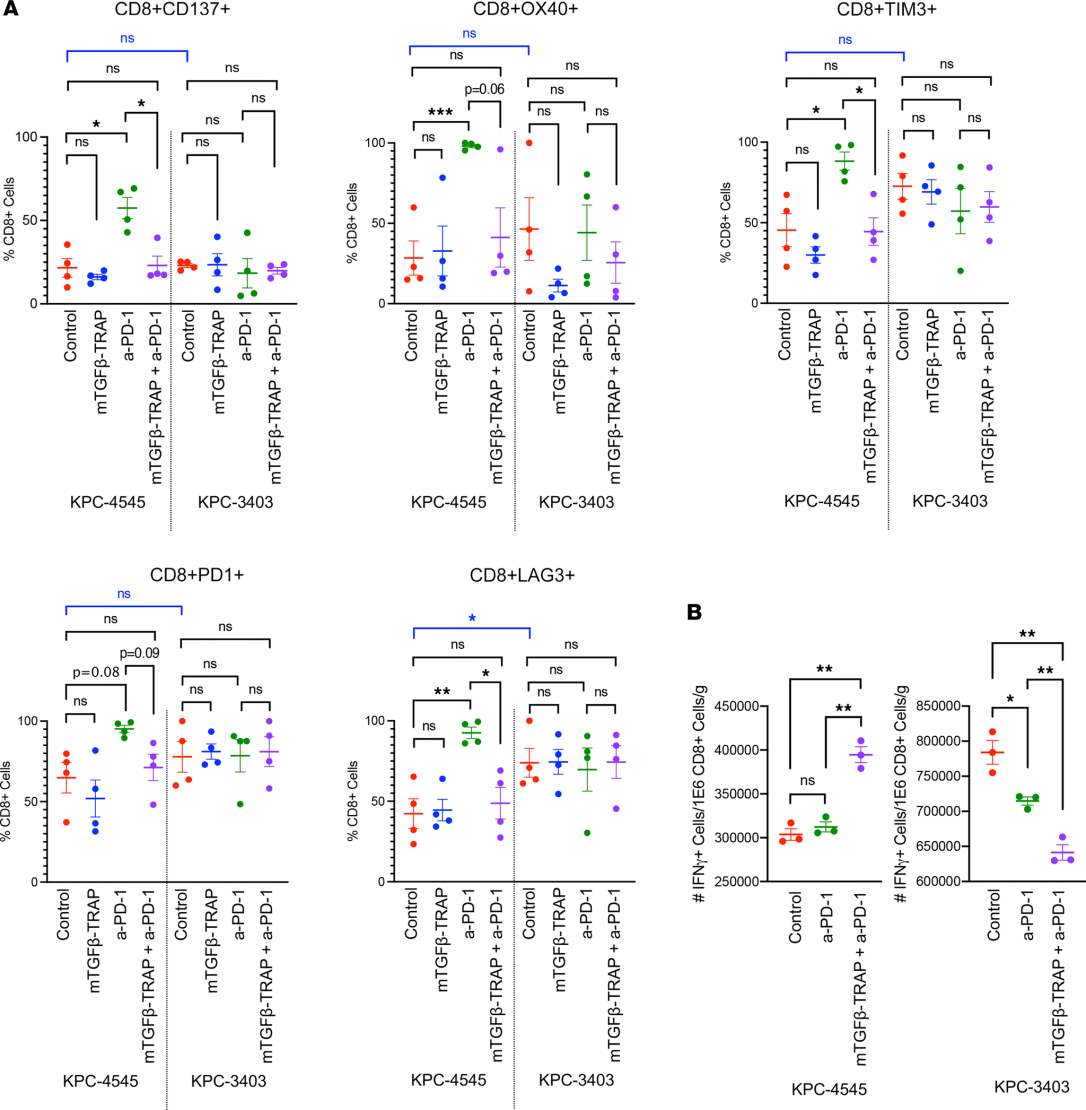
The combination of mTGF-β-TRAP and a-PD-1 has limited effect on T cell activation but may reverse T cell exhaustion in PDAC orthotopic tumors. (**A** and **B**) Flow cytometry was performed on isolated tumor-infiltrating immune cells from resected orthotopic tumor on day 13 (data in **A** and **B** are from separate experiments). The following tumor-infiltrating immune cells were analyzed: (**A**) percentage of CD137^+^, OX40^+^, LAG3^+^, TIM3^+^, and PD-1^+^ cells among CD45^+^CD8^+^ cells (*n* = 4 mice per group). (**B**) The number of isolated IFN-γ^+^ tumor-infiltrating immune cells was normalized to tumor weight per 1 × 10^6^ CD8^+^ cells (*n* = 5 mice per group, pooled and measured in triplicate). Data represent mean ± SEM. *, *P* < 0.05; **, *P* < 0.01; ***, *P* < 0.001, by multiple Student’s *t* test with Holm-Šidák correction.

Although our 2 tumor models would not be sufficient for establishing the specific role of TGF-β-TRAP in treating PDAC liver metastasis, they would be valuable for us to elucidate the potential mechanisms in the TME underlying the heterogeneity of response to TGF-β-TRAP treatment. We thus performed flow analysis of the primary orthotopic tumors in the KPC-4545 and KPC-3403 models. Mice were treated as described in Figure 1; however, on day 13, all mice were sacrificed, and pancreatic tumors were harvested to analyze tumor-infiltrating leukocytes. Flow analysis of T cells and their subtypes was performed (Supplemental Figure 4). We observed no differences at baseline between KPC-4545 and KPC-3403 tumors in the percentages of CD4^+^ or CD8^+^ T cells among CD45^+^ cells (Supplemental Figure 5A) or their normalized cell counts per 1 × 10^6^ cells (Supplemental Figure 5B). Additionally, no differences in the baseline percentages of CD137^+^, OX40^+^, TIM3^+^, and PD-1^+^ cells among CD8^+^ (Figure 2A) and CD4^+^ T cells (Supplemental Figure 5C) were observed. Although KPC-3403 tumors had a higher percentage of CD8^+^LAG3^+^ cells among CD8^+^ T cells compared with KPC-4545 tumors, no differences in the percentage of CD4^+^LAG3^+^ cells were observed. The baseline percentages of CD4^+^FoxP3^+^ T regulatory cells (Tregs) were also comparable between KPC-4545 and KPC-3403 tumors (Supplemental Figure 5D and Supplemental Figure 6). These findings demonstrate that there were minimal to no differences in baseline T cell functional status for both KPC tumor models.

We then investigated the change of T cell functional status in response to TGF-β-TRAP treatment with or without a-PD-1. We found that there was no effect of mTGF-β-TRAP alone on the percentage of CD8^+^CD137^+^ cells (Figure 2A). Although a-PD-1 monotherapy enhanced the percentage of CD8^+^CD137^+^ and CD8^+^OX40^+^ T cells, the addition of mTGF-β-TRAP to a-PD-1 resulted in a significant decrease in CD8^+^CD137^+^ T cells and decreased trend in CD8^+^OX40^+^ T cells in the KPC-4545 model (Figure 2A). We did not observe a quantitative effect of TGF-β-TRAP treatment on CD4^+^CD137^+^ cells or CD4^+^OX40^+^ cells in either KPC-4545 or KPC-3403 tumors (Supplemental Figure 5C). These findings suggest that TGF-β-TRAP plays a limited role in both the CD4^+^ and CD8^+^ T cell activation processes, explaining why TGF-β-TRAP monotherapy did not have an antitumor and antimetastasis effect (Figure 1 and Supplemental Figure 1). We also did not observe an impact of TGF-β-TRAP on Tregs, although there was a modest effect of a-PD-1 and a-PD-1+TGF-β-TRAP on the percentage of Tregs among CD4^+^ T cells in the KPC-3403 tumors (Supplemental Figure 5D).

In contrast, although a-PD-1 monotherapy significantly increased the percentage of exhausted T cells including CD8^+^LAG3^+^, CD8^+^TIM3^+^, CD4^+^LAG3^+^, CD4^+^TIM3^+^, and CD4^+^PD-1^+^ T cells in the KPC-4545 tumors, addition of mTGF-β-TRAP to a-PD-1 normalized the percentage of exhausted T cell subtypes in KPC-4545 tumors back to baseline (Figure 2A and Supplemental Figure 5C). No changes in the percentage of exhausted CD8^+^ or CD4^+^ T cells were observed in the KPC-3403 tumors following treatment (Figure 2A and Supplemental Figure 5C). These results suggested that, while a-PD-1 treatment further induces T cell exhaustion, adding TGF-β-TRAP may reverse T cell exhaustion in both CD4^+^ and CD8^+^ T cells in KPC tumors with liver metastasis tropism. The response of the T cell exhaustion status to TGF-β-TRAP treatment appears to be heterogenous in KPC tumors with distinct metastatic tropism.

### Heterogeneous response of cytotoxic effector T cells to combination mTGF-β-TRAP and a-PD-1 treatment in KPC tumors.

To further evaluate effector T cell response to TGF-β-TRAP, we performed intracellular staining for IFN-γ (Supplemental Figure 7). For KPC-4545 orthotopic tumors, mTGF-β-TRAP+a-PD-1 treatment resulted in an increased number of IFN-γ^+^ cells normalized to 1 × 10^6^ CD8^+^ cells per gram of tumor compared with control and a-PD-1 alone (Figure 2B). In contrast, for KPC-3403 orthotopic tumors, the mTGF-β-TRAP+a-PD-1 combination resulted in fewer IFN-γ^+^ cells normalized to 1 × 10^6^ CD8^+^ cells per gram of tumor (Figure 2B). These findings suggested a heterogeneous response of cytotoxic effector T cells to the mTGF-β-TRAP+a-PD-1 treatment in KPC tumors.

### Baseline heterogeneity of myeloid cell subtypes in different KPC tumors.

**Figure 3:**
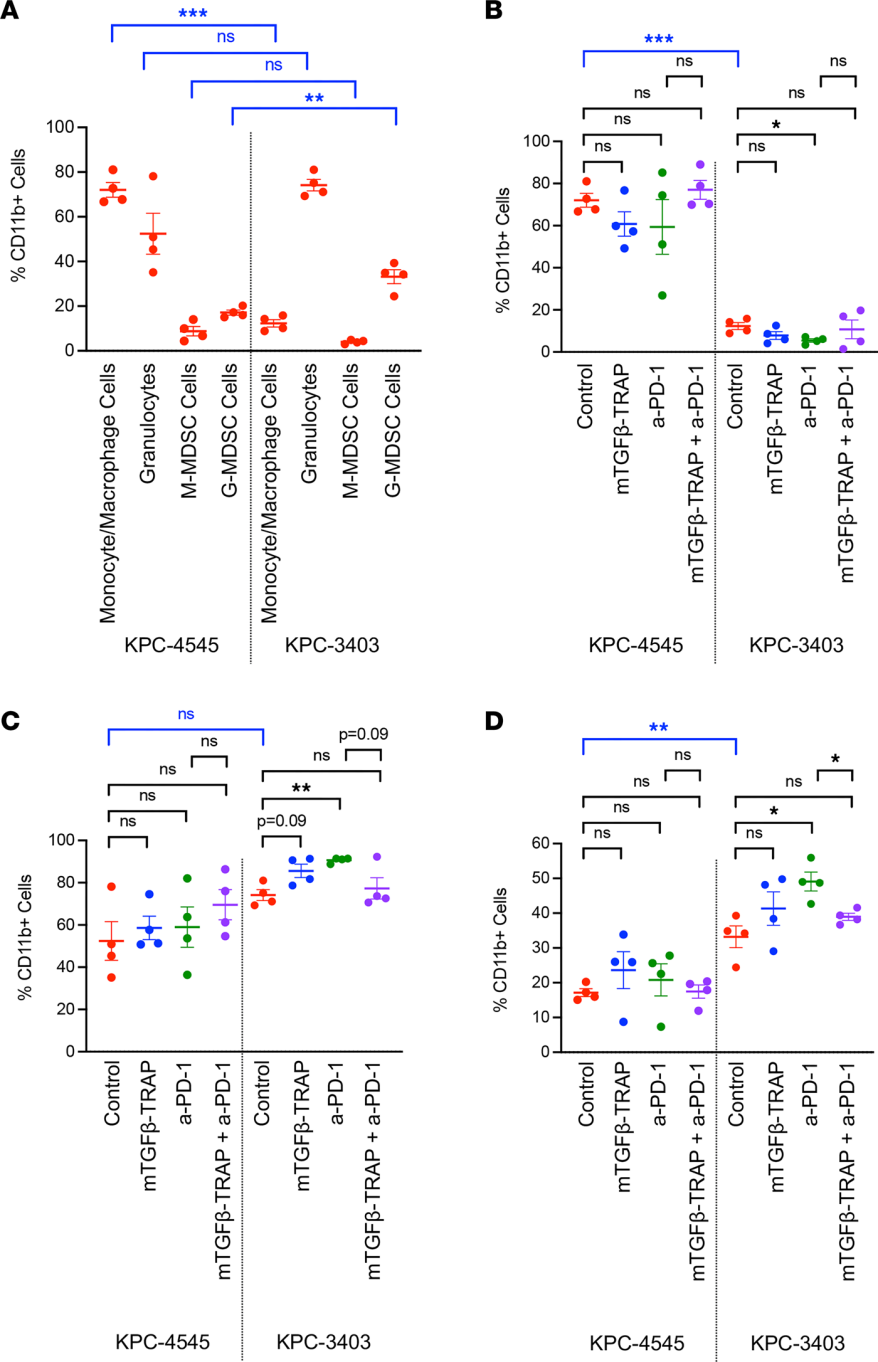
Baseline heterogeneity of myeloid cell subtypes in different KPC tumors. Flow cytometry was performed on isolated tumor-infiltrating immune cells from resected orthotopic tumor on day 13. The following isolated tumor-infiltrating immune cells were analyzed: (**A**) percentage of monocyte/macrophage cells, granulocytes, monocytic MDSCs (M-MDSCs), and G-MDSCs among CD45^+^CD11b^+^ cells at baseline (*n* = 4 mice per group) and (**B**–**D**) percentage of monocyte/macrophage cells (**B**), granulocytes (**C**), and G-MDSC cells (**D**) among CD45^+^CD11b^+^ cells by treatment group. Data represent mean ± SEM. *, *P* < 0.05; **, *P* < 0.01; ***, *P* < 0.001, by multiple Student’s *t* test with Holm-Šidák correction.

Given TGF-β’s role in myeloid cell differentiation and reprogramming, we sought to determine the impact of TGF-β-TRAP on myeloid cells by performing flow analysis of myeloid cell subtypes (Supplemental Figure 8). At baseline, KPC-4545 orthotopic tumors had a higher percentage of monocytes/macrophages (CD45^+^CD11b^+^F4/80^+^) among CD45^+^CD11b^+^ cells compared with KPC-3403 but lower percentages of granulocytic-MDSCs (G-MDSCs; CD45^+^CD11b^+^Ly6C^lo^Ly6G^+^) (Figure 3A). No significant differences in the percentages of monocytic-MDSCs (M-MDSCs; CD45^+^ CD11b^+^Ly6C^hi^Ly6G^–^) and granulocytes (CD45^+^CD11b^+^Ly6G^+^) were observed between the 2 KPC tumor types (Figure 3A).

When assessing changes of the myeloid cell subpopulations in response to treatment, we observed that addition of single-agent mTGF-β-TRAP did not affect the percentage of monocytes/macrophages (Figure 3B), granulocytes (Figure 3C), or M-MDSCs (Supplemental Figure 5E) among CD45^+^CD11b^+^ cells in either KPC tumor type. However, we did note a nonsignificant decrease in the percentage of G-MDSCs in the KPC-4545 tumors following addition of mTGF-β-TRAP in the combination treatment group compared with the a-PD-1 monotherapy group (Figure 3D). In the KPC-3403 tumor, though a-PD-1 increased the percentage of granulocytes and G-MDSCs in KPC-3403 tumors, the addition of mTGF-β-TRAP in the combination group decreased the percentage of G-MDSCs compared with the a-PD-1 monotherapy group (Figure 3, C and D). Taken together, while there is a baseline heterogeneity of myeloid cell subtypes in different KPC tumors, single-agent TGF-β-TRAP does not change the relative abundance of myeloid cell subpopulations infiltrating tumors compared with control. Nevertheless, it could be possible that mTGF-β-TRAP modulates the functions of myeloid cell populations.

### Single-nuclear RNA sequencing supports TGF-β signaling blockade by TGF-β-TRAP.

**Figure 4:**
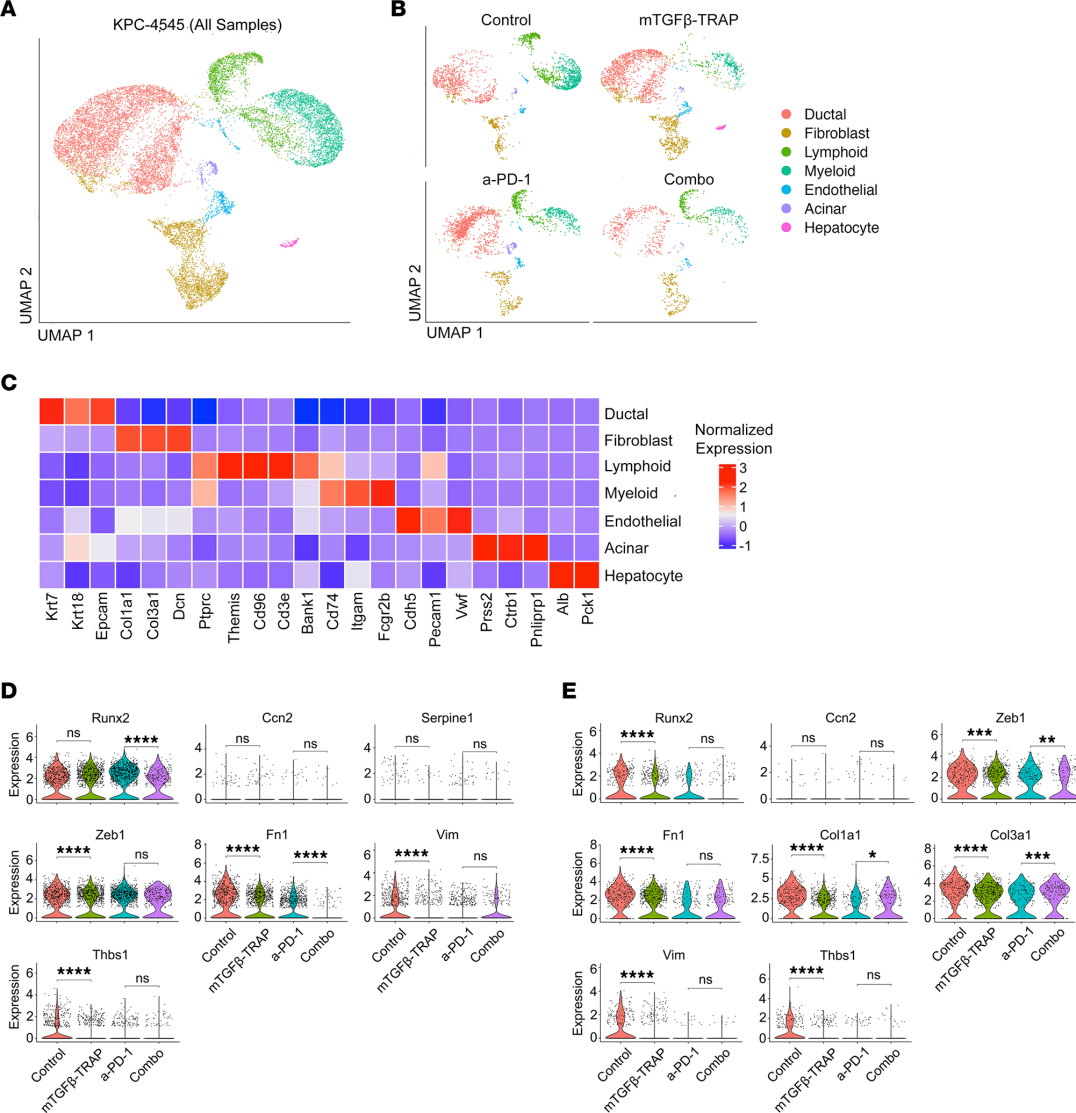
snRNA-Seq of treated and untreated KPC-4545 orthotopic tumor. (**A** and **B**) Uniform manifold approximation and projection (UMAP) embedding of single-nucleus profiles of representative cell types across the 6 KPC-4545 samples combined (**A**) and 4 TGF-β-TRAP samples separated by treatment (**B**). (**C**) Heatmap of mean normalized expression of selected marker genes across captured cell types. (**D** and **E**) Violin plot of normalized gene expression of TGF-β downstream target genes across samples in all tumor epithelial cells (**D**) and CAFs (**E**). Violin plots display log-normalized gene expression using Seurat’s default VlnPlot function. Each point represents the log-normalized expression value of a particular gene in one specific cell from the single-nuclear RNA sequencing data. Combo represents a-PD-1+mTGF-β-TRAP. *P < 0.05, **P < 0.01, ***P < 0.001, ****P < 0.0001 by Mann-Whitney *U* test.

To further explore the effects of TGF-β-TRAP on the functions of immune and stromal cells within the TME, we generated single-nuclear RNA-sequencing (snRNA-Seq) profiles of tumors from each of the 6 treatment groups including 1) isotype controls, 2) mTGF-β-TRAP, 3) a-PD-1, 4) anti–IL-1β, 5) mTGF-β-TRAP+a-PD-1, and 6) anti–IL-1β+a-PD-1 within the same batch. Groups 4 and 6 belong to another concurrent study and were clustered initially within the batch but not included for further downstream analysis. The snRNA-Seq data of all 6 tumors from the KPC-4545 samples were merged into 1 object with a total of 13,578 nuclei and were normalized. We applied principal component analysis on the top 2,000 highly variable genes across all cells to identify 15 clusters, which were annotated with gene markers from the literature (Figure 4, A–C). Compared with our previous approach with single-cell RNA-Seq (34), we were able to more comprehensively recover and capture PDAC stromal cells, including CAFs, immune cells, and endothelial cells, by using snRNA-Seq (35).

To understand whether TGF-β-TRAP inhibits or alters TGF-β signaling in tumor cells and CAFs, we analyzed the TGF-β downstream target genes (Figure 4, D and E) as well as other signaling intermediates in the canonical TGF-β signaling cascade (Supplemental Figure 9, A and B) in tumor epithelial cells and CAFs, respectively. The results suggested that TGF-β-TRAP inhibits TGF-β signaling in tumor cells and CAFs. Within CAFs, we also noted a significant increase in the expression of *Smad6* (Supplemental Figure 9B), which is known to inhibit TGF-β signaling, following combination treatment of TGF-β-TRAP+a-PD-1 compared with a-PD-1 alone, providing a potential mechanism for the synergistic effect of TGF-β-TRAP and a-PD-1 on TGF-β signaling. Interestingly, we noted a significant decrease in *Tgfbr1* expression in CAFs but not the expression of any *Smad* genes following a-PD-1 treatment compared with the isotype control (Supplemental Figure 9B), suggesting that a-PD-1 treatment may suppress TGF-β signaling by a mechanism different from that of TGF-β-TRAP.

### TGF-β-TRAP modulates CAF heterogeneity and composition of CAF subclusters.

TGF-β signaling has been shown to be critical for modulating the heterogeneity of CAFs in the TME and thereby modulating the antitumor response to PDAC (25). To understand the effects of TGF-β-TRAP on CAF heterogeneity, we isolated the previously identified CAF population and performed subclustering analysis, resulting in the identification of 3 major CAF clusters (Supplemental Figure 10, A and B, and Supplemental Table 1). At baseline (isotype control group), all 3 CAF clusters initially expressed both iCAF features including *Svep1*, *Plpp3*, *Fbn1*, *Ccdc80*, *C3*, *Abl2*, and *Fbln2*, and myCAF features including *Tpm1*, *Igfbp3*, *Thbs2*, *Spp1*, *Col12a1*, *Dock8*, and *Col15a1* (Supplemental Figure 10, C–E). This suggests the presence of a more homogenous CAF phenotype within the PDAC TME, consistent with the result shown previously in this mouse model of KPC tumor with high liver metastasis potential (31).

Following TGF-β-TRAP treatment, CAF cluster 1 skewed toward an iCAF phenotype by having a more decreased expression in myCAF signature genes than iCAF signature genes (Supplemental Figure 10F). CAF cluster 2 skewed toward an iCAF phenotype, displaying stronger expression of iCAF markers and weaker expression of myCAF markers (Supplemental Figure 10G). On the other hand, CAF cluster 3 skewed toward a myCAF phenotype by displaying stronger myCAF and weaker iCAF gene expression (Supplemental Figure 10H). This result suggested that TGF-β-TRAP treatment restores the CAF heterogeneity, which would need further validation by using other tumor models in the future. Following a-PD-1 treatment, cluster 1 again skewed toward an iCAF phenotype by having a more decreased expression in myCAF signature genes than iCAF signature genes (Supplemental Figure 10I). Cluster 2 also skewed toward a more iCAF-dominant phenotype with relatively stronger expression of iCAF markers and weaker expression of myCAF markers (Supplemental Figure 10J). However, cluster 3 maintained both iCAF and myCAF features (Supplemental Figure 10K). This result suggested that a-PD-1 treatment has some similar effect but not the same as TGF-β-TRAP treatment on CAF heterogeneity. Finally, following the combination treatment with a-PD-1+TGF-β-TRAP, as anticipated, both clusters 1 and 2 skewed toward an iCAF phenotype, with both clusters showing relatively stronger iCAF and weaker myCAF signature gene expression (Supplemental Figure 10, L and M). Similar to a-PD-1 treatment, cluster 3 maintained features of both CAF phenotypes (Supplemental Figure 10N).

**Figure 5:**
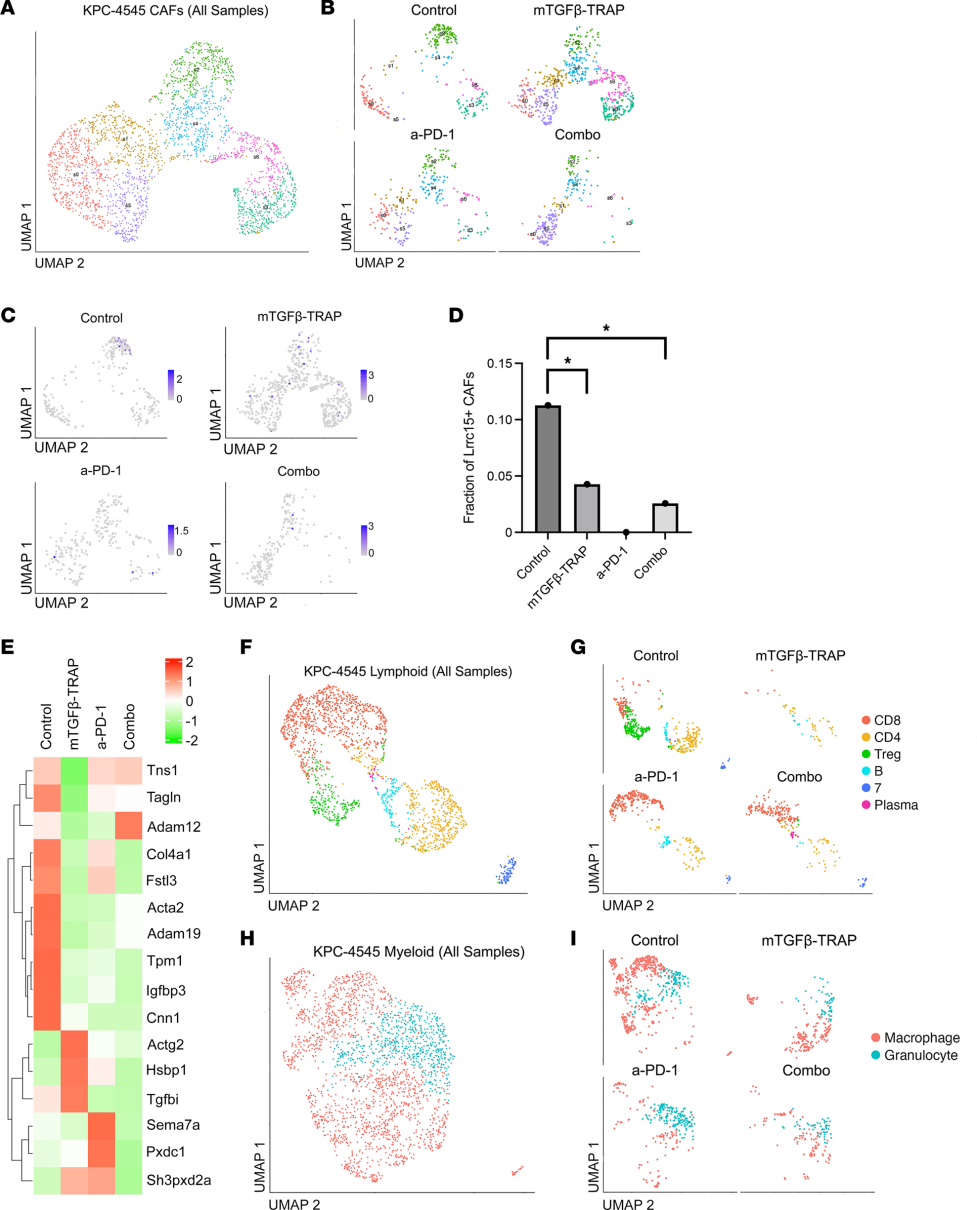
TGF-β-TRAP modulates CAF heterogeneity and immune landscape of the KPC-4545 tumor. (**A** and **B**) Reclustered CAFs across all 6 KPC-4545 samples combined (**A**) and 4 TGF-β-TRAP samples separated by treatment (**B**). (**C**) Normalized expression of *Lrrc15* in CAFs across TGF-β-TRAP samples. (**D**) Fraction of *Lrrc15*^+^ nuclei in the *Lrrc15*^+^ CAF cluster consisting of s2 and s4 across the TGF-β-TRAP samples. *Lrrc15*^+^ CAFs were compared with Fisher’s exact test. (**E**) Heatmap visualizing the normalized mean expression of F-TBRS signature genes in CAF cluster 2 across samples. (**F** and **G**) UMAP of reclustered and annotated lymphoid cells across all KPC-4545 samples combined (**F**) and separated by treatment (**G**). (**H** and **I**) UMAP of reclustered myeloid cells annotated as macrophages or granulocytes across all KPC-4545 samples (**H**) and separated by treatment (**I**). Combo represents a-PD-1+mTGF-β-TRAP. *, *P* < 0.05.

We further identified 7 CAF subclusters, labeled s0–s6, within the 3 major CAF clusters: s0, s1, s5 in cluster 1; s2, s4 in cluster 2; and s3, s6 in cluster 3 (Figure 5A). At baseline, essentially only s0, s2, and s3 were present, with 1 subcluster in each CAF cluster (Figure 5B). Following TGF-β-TRAP treatment, s1 and s5 became more prominent in cluster 1; s4 became more prominent in cluster 2; and s6 became more prominent in cluster 3. Following a-PD-1 treatment, similar to the result following TGF-β-TRAP treatment, s1 and s5 constituted cluster 1, and s4 became bigger in cluster 2; however, there were fewer CAFs in s3 and s6. Following combination treatment, s0, s3, and s6 essentially disappeared, with s5 remaining in cluster 1 and s2 and s4 remaining in cluster 2. Therefore, in addition to the change of CAF heterogeneity, TGF-β-TRAP treatment, a-PD-1 treatment, or their combination also modulated the infiltration of these subclusters of CAFs, which warrant further validation and characterizations.

Given the large population of CAF s5, which was not present in the control sample, following combination treatment, we sought to better characterize this subcluster (Figure 5B). When compared with all other CAF subclusters, the top differentially expressed gene of CAF s5 was *Thsd4* (Supplemental Figure 11 and Supplemental Table 2). Inhibition of *Thsd4* expression has been reported to promote cell migration and metastasis in colorectal cancer (CRC) (36). In turn, overexpression of *Thsd4* decreased phosphorylation of Smad2 and Smad3 and impeded invasion of CRC (36). As such, this subcluster could mediate the reduced liver metastasis observed in the KPC-4545 model following combination treatment.

### TGF-β-TRAP suppresses Lrrc15^+^ CAF subclusters.

Recent studies have identified a TGF-β–driven, LRRC15^+^ CAF lineage that suppresses antitumor immunity and is associated with poor response to a-PD-1 in several malignancies (28, 29). We subsequently identified s2 as this potential immunosuppressive, *Lrrc15*-expressing CAF subpopulation (Figure 5C). As s2 does not appear to change markedly following TGF-β-TRAP, a-PD-1, or their combination treatment, we examined *Lrrc15*-expressing cells within cluster 2 (s2, s4 combined) and compared the changes of their proportions following different treatments. There was a significant decrease in the proportion of *Lrrc15*^+^ CAFs in cluster 2 CAFs following TGF-β-TRAP and combination treatment, respectively, compared with the control treatment (Figure 5D). Furthermore, cluster 2 CAFs at baseline showed a strong enrichment of pan-fibroblast TGF-β response signature (F-TBRS), which measures the TGF-β pathway activity in fibroblasts (Figure 5E), suggesting TGF-β signaling as a key driver of cluster 2 CAFs. The enrichment of the F-TBRS signature in cluster 2 became weaker following TGF-β-TRAP, a-PD-1, or a-PD-1+TGF-β-TRAP treatment.

### TGF-β-TRAP treatment modulates both lymphoid and myeloid cells in PDAC.

Next, we subclustered the lymphoid cells (Figure 5F). In untreated tumors, we identified CD8^+^ T cell, CD4^+^ T cell, B cell, a Ccr7^+^ T cell population (cluster 7), and a large Treg population (Figure 5G). Following TGF-β-TRAP monotherapy, in addition to the depletion of Tregs, we noted a decreased infiltration of lymphoid cells overall (Figure 5G). Based on our snRNA-Seq, there was an increased infiltration of CD8^+^ T cells along with the depletion of Tregs following a-PD-1 monotherapy, which may be specific for the KPC-4545 tumor model (Figure 5G). Subsequently, we observed both an increased infiltration of CD8^+^ T cells and depletion of Tregs following combination of a-PD-1+TGF-β-TRAP (Figure 5G).

Consistent with our flow cytometry data, the infiltrated CD8^+^ T cells showed comparable to higher level expression of T cell exhaustion genes following a-PD-1 treatment, suggesting the presence of an exhausted CD8^+^ T cell population (Supplemental Figure 12). We also found a reversal in T cell exhaustion following addition of mTGF-β-TRAP to a-PD-1, with a decrease in expression of exhaustion genes such as *Tigit*, *Pdcd1*, *Itgae*, *Tox*, and *Entpd1* in the combination group compared with the a-PD-1 single-agent group (Supplemental Figure 12). The mTGF-β-TRAP single-agent group was not included in analysis given the low infiltration of CD8^+^ T cells in this sample. The increased infiltration of cytotoxic CD8^+^ T cells compared with the control sample and reversed T cell exhaustion may mediate the increased overall survival and enhanced tumor control in the combination group.

Within the myeloid subclustering, we annotated nuclei as macrophages and granulocytes based on markers from literature (Figure 5H). There was an overall decrease in the absolute number of both granulocytes and macrophages after TGF-β-TRAP treatment (Figure 5I). Additionally, there was a shift in the macrophage population on the UMAP following TGF-β-TRAP monotherapy, suggesting a reprogramming of macrophages. In contrast, following a-PD-1 treatment, there was an increase in the fraction of granulocytes compared with control (Figure 5I). Nevertheless, a-PD-1+TGF-β-TRAP combination treatment resulted in the decrease of both granulocytes and macrophages. While this decrease in granulocytes observed in the snRNA-Seq data is not seen in our KPC-4545 tumor flow analysis, there is a decreased trend in the fraction of G-MDSCs following combination treatment compared with a-PD-1 treatment in our flow analysis. Therefore, it is possible the decrease in the granulocyte population captured in the snRNA-Seq was contributed predominantly by G-MDSCs, though we were not able to clearly differentiate between the 2 closely related cell types in the snRNA-Seq analysis given their highly overlapped transcriptional profiles (37, 38). This would be in line with the decreased trend in the percentage of G-MDSCs among granulocytes observed in the flow analysis following addition of mTGF-β-TRAP to a-PD-1 treatment (25.1%) compared with a-PD-1 alone (35.3%).

### TGF-β-TRAP suppresses neutrophil degranulation and the response of CD4^+^ T cells to neutrophil degranulation.

**Figure 6:**
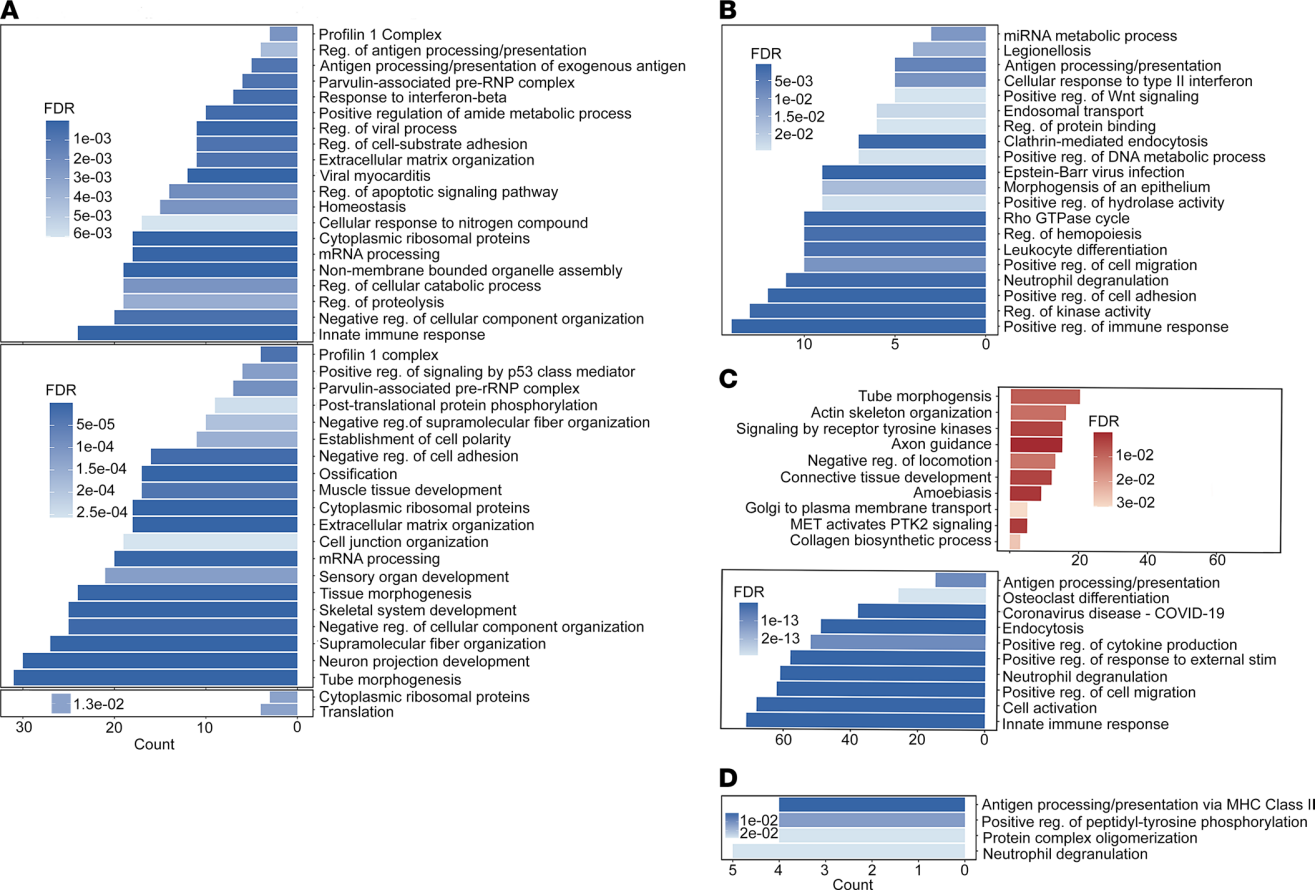
Pathway analysis of differentially expressed genes after TGF-β-TRAP treatment. (**A**–**D**) Top significant pathways (FDR < 0.05) from significant differentially expressed genes (Log_2_FC < –0.5 or Log_2_FC > 0.5 and adjusted *P* < 0.05) between control and TGF-β-TRAP–treated samples for all 3 major clusters of CAFs (top, middle, and bottom) (**A**), CD4^+^ T cells (**B**), macrophages (**C**), and granulocytes (**D**). Blue and red indicate downregulated and upregulated after TGF-β-TRAP treatment, respectively. FDR, false discovery rate; FC, fold change.

We conducted differential gene expression analysis among treatment groups including control versus TGF-β-TRAP and a-PD-1 versus a-PD-1+TGF-β-TRAP in cell types of interest (Supplemental Figure 13, A–N). Likely due to small cell numbers, we did not observe significantly enriched pathways from differentially expressed genes (DEGs) between a-PD-1 and the a-PD-1+TGF-β-TRAP groups. Across all 3 clusters of CAFs, functional enrichment of DEGs showed that pathways involved in the regulation of mRNA and protein processing, including cytoplasmic ribosomal proteins and regulation of cellular catabolic process, were downregulated after TGF-β-TRAP treatment (Figure 6A). Downregulated genes following TGF-β-TRAP treatment were also enriched in ECM organization, regulation of cell-substrate adhesion, and profilin 1 complex, suggesting the ability of TGF-β-TRAP to alter the ECM and the interaction with ECM of CAFs, particularly for CAF clusters 1 and 2.

Likely due to a low CD8^+^ T cell number in the TGF-β-TRAP–treated tumor, no significantly DEGs were identified between the TGF-β-TRAP–treated tumor and control-treated tumor in CD8^+^ T cells (Supplemental Figure 13G). However, we did observe that downregulated DEGs in CD4^+^ T cells following TGF-β-TRAP monotherapy were enriched in the pathways including positive regulation of Wnt signaling pathway, positive regulation of cell migration, cellular response to type II interferon, positive regulation of immune response, and neutrophil degranulation (Figure 6B).

Within the monocyte/macrophage population, upregulated DEGs after TGF-β-TRAP treatment were enriched in pathways involved in cellular organization and transport, such as tube morphogenesis, actin cytoskeleton organization, and Golgi to plasma membrane transport (Figure 6C). TGF-β-TRAP also appears to affect cellular motility within the monocytic/macrophage population. Upregulation of the negative regulation of locomotion pathway and a corresponding downregulation of the positive regulation of cell migration pathway suggests that TGF-β-TRAP may inhibit the motility of macrophages in the TME. TGF-β-TRAP also affected the cellular function of macrophages by downregulating pathways such as innate immune response, positive regulation of cytokine production, and antigen processing and presentation. Within the granulocytic population, TGF-β-TRAP treatment resulted in the downregulation of the pathways including antigen processing and presentation of exogenous peptide antigen via MHC class II, positive regulation of peptidyl-tyrosine phosphorylation, and protein complex oligomerization (Figure 6D). Interestingly, the neutrophil degranulation pathway was also enriched in the downregulated DEGs in granulocytes, supporting a role of TGF-β-TRAP in suppressing neutrophil degranulation and the response of CD4^+^ T cells to neutrophil degranulation.

### TGF-β-TRAP reprograms myeloid cells and CAFs by modifying ligand-receptor signaling.

**Figure 7:**
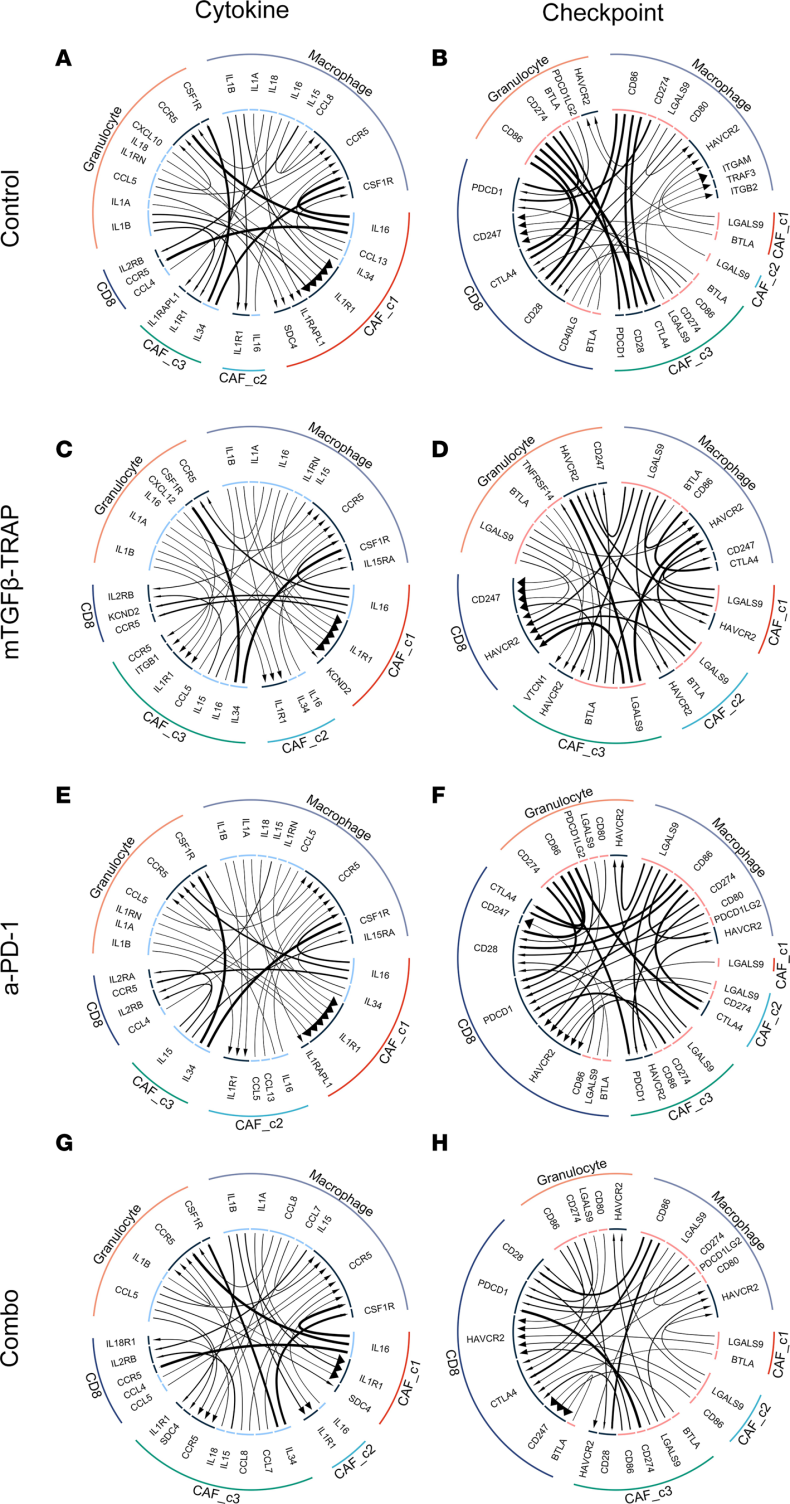
Cytokine and checkpoint ligand-receptor analysis between CAFs and immune cells. (**A**–**H**) Chord diagram of ligand-receptor interactions between all 3 major clusters of CAFs, CD8^+^ T cells, macrophages, and granulocytes inferred by iTALK. Each row represents a treatment sample in the order of control (**A** and **B**), mTGF-β-TRAP (**C** and **D**), a-PD-1 (**E** and **F**), and combo (a-PD-1+mTGF-β-TRAP) (**G** and **H**) from top to bottom. Each column represents a category defined by iTALK, including cytokines and checkpoint. CTLA4, cytotoxic T lymphocyte antigen-4.

To understand the interactions between different cell types and the impact of TGF-β-TRAP on such interactions, we used the iTALK package to infer ligand-receptor interaction with the snRNA-Seq data. Here we focused on 4 cell types, including CAFs, CD8^+^ T cells, granulocytes, and macrophages. Interactions were categorized into growth factor, cytokines, checkpoint, and other based on the type of ligands (Figure 7 and Supplemental Figure 14). We compared the top 30 ligand-receptor interactions within each category among different treatment groups (Supplemental Tables 3–18). In the control-treated tumor, CAFs in all 3 clusters appeared to be the main source of the growth factor ligands (Supplemental Figure 14A and Supplemental Table 3) including TGF-βs, FGFs, and VEGFs, which bind to the receptors including CD44, TGF-βRs, and ITGs. Such a pattern did not change in the TGF-β-TRAP–, a-PD-1–, or TGF-β-TRAP+a-PD-1–treated tumors (Supplemental Tables 4, 11, and 12).

In the control-treated tumor, IL-1 signaling, including IL-1A, IL-1β, and IL-18, appeared to be the dominant cytokine-type ligand-receptor interaction between macrophages/granulocytes and CAFs (Figure 7A). However, following TGF-β-TRAP, a-PD-1, or TGF-β-TRAP/a-PD-1 combo treatments, IL-15 signaling became more prominent, consistent with an enhanced effector T cell response. Following TGF-β-TRAP treatment, IL-16 signaling became more prominent, and its receptors also became more diverse, including both CCR5 and KCND2 (Figure 7C). However, following a-PD-1 or TGF-β-TRAP+a-PD-1 combo treatments, IL-16 signaling became less prominent but derived exclusively from CAFs (Figure 7, E and G). In contrast, CCL5, another ligand of CCR5, almost disappeared following the TGF-β-TRAP treatment. Interestingly, CCR5 signaling was similar between the a-PD-1–treated tumor and the control-treated tumor. However, the ligands of CCR5 in the TGF-β-TRAP+a-PD-1 combo treatment expanded to CCL7 and CCL8 from macrophages, which may mediate the previously described immune agonist activity of CCR5 (37, 38). Nevertheless, the CCL5/CCR5 axis, which is anticipated to recruit immunosuppressive macrophages, remained prominent in this combo-treated tumor. Interestingly, the CCL5 signaling appeared to come exclusively from granulocytes following the combination treatment, suggesting that the addition of a granulocyte/neutrophil-targeting agent may be beneficial.

Costimulatory and checkpoint signals were altered following TGF-β-TRAP treatment (Figure 7). PD-L1–PD-1 interaction and CD80/CD86–CTLA-4 interaction appeared to be eliminated following TGF-β-TRAP treatment. On the other hand, T cell exhaustion signals mediated by LGALS9/TIM3 were amplified. Compared with the a-PD-1–treated tumor, the tumor treated with TGF-β-TRAP+a-PD-1 also demonstrated enhanced LGALS9/TIM3 signaling. However, the tumor treated with combination therapy maintained CD80/CD86–CTLA-4 signaling. We did not observe any major differences in other ligand-receptor signaling axes among different treatment groups. Together, the iTALK analysis suggests that TGF-β-TRAP treatment modulates cell-cell signaling, particularly the CCL5/CCR5 axis and costimulatory/checkpoint signaling from CAFs and myeloid cells, and that such reprogramming is further modulated by the combination with a-PD-1.

### Murine PDACs with different organ-specific metastasis potentials have different TMEs.

Next, we attempted to understand how the TME of the KPC-4545 model, which has metastatic liver tropism, is different from that of the KPC-3403 model, which has a metastatic lung tropism. We generated snRNA-Seq profiles from the KPC-3403 tumors (Supplemental Figure 15A) following the same procedure as the KPC-4545 tumors. As described above, within each model, we first merged tumors from all treatment groups into 1 single data set, totaling 9,021 nuclei for KPC-3403. Principal component analysis and unsupervised clustering identified 18 clusters in the KPC-3403 model, which were annotated according to signature gene markers (Supplemental Figure 15A). Interestingly, we observed a cluster annotated as adipocytes in the KPC-3403 model but not in the KPC-4545 model. On the other hand, we observed a cluster of acinar cells in the KPC-4545 model (Figure 4A), but not in the KPC-3403 model, possibly due to a small variation in the dissection of tumors from the normal portion of the pancreas. We also noticed repeatedly the low fraction reads in cells, which distinguished KPC-3403 from KPC-4545, and subsequently as anticipated, the low-confidence mapping to the transcriptome rate in the untreated tumor of the KPC-3403 model (Supplemental Table 19). We then mixed the snRNA-Seq data from all 4 TGF-β-TRAP treatment groups together for the initial comparisons between cell types of interest. Three main clusters of CAFs were also identified in the KPC-3403 model (Supplemental Figure 15B). Overall, in the 4 TGF-β-TRAP–related KPC-3403 samples combined, CAF cluster 1 skewed toward a myCAF phenotype while CAF clusters 2 and 3 skewed toward an iCAF phenotype (Supplemental Figure 15, C–E). This relative CAF heterogeneity was maintained before (Supplemental Figure 15, F–H) and after treatment (Supplemental Figure 15, I–Q) in KPC-3403.

In contrast, we did not observe obvious differences by comparing lymphoid and myeloid clusters between the KPC-4545 (Supplemental Figure 16, A and D) and KPC-3403 (Supplemental Figure 16, B and E) tumors. In the KPC-3403 model, we were able to identify the CD8^+^, CD4^+^, Treg, and B cell populations and a cluster (cluster 6) with uncertain identity. However, we noticed a higher fraction of CD8^+^ T cells and lower fraction of B cells in KPC-4545 than KPC-3403 tumors (Supplemental Figure 16C). Nevertheless, we would not know whether such differences were the intrinsic features of 2 different KPC tumors associated with different metastasis potential or due to the different responses to the treatments; however, such comparisons provide a clue for future studies.

## Discussion

To the best of our knowledge, this is the first study that evaluates the novel TGF-β-TRAP molecule in a preclinical murine PDAC model and the first that comprehensively investigates the role of TGF-β-TRAP in combination with a-PD-1 in modulating the PDAC TME and the mechanisms underlying the heterogeneous response to TGF-β depletion using snRNA-Seq. This study showed that TGF-β-TRAP modulates CAF heterogeneity, counteracts a-PD-1–induced T cell exhaustion, enhances cytotoxic effector T cell infiltration, suppresses granulocyte degranulation and CD4^+^ T cell response to granulocyte degranulation signals, and modulates ligand-receptor signaling, particularly the CCL5/CCR5 axis and costimulatory/checkpoint signaling from CAFs and myeloid cells, all preferentially in the tumor model with a liver metastasis tropism.

We did not observe differences between human TGF-β-TRAP (AVID200) and mouse TGF-β-TRAP; therefore, we only repeated selected experiments with both human and mouse TGF-β-TRAP. Antitumor effect of TGF-β-TRAP was demonstrated by multiple syngeneic KPC tumor models with KPC-4545 cells while using KPC-3403 cells as a control. The results would not be sufficient to conclude that TGF-β-TRAP preferentially targets KPC tumors with liver metastasis potentially. Nevertheless, this study is more focused on demonstrating the impact of different TMEs represented by KPC-4545 and KPC-3403 tumors on the response to TGF-β-TRAP with or without a-PD-1 treatment.

Our result showed that TGF-β-TRAP and combination treatment are capable of depleting *Lrrc15*^+^ CAFs, which could mediate the increased survival in the KPC-4545 model compared with the control group. Interestingly, a-PD-1 was also able to deplete *Lrrc15*^+^ CAFs and suppress the F-TBRS gene signature and TGF-β downstream genes. Nevertheless, TGF-β-TRAP and a-PD-1 treatments targeted different TGF-β downstream pathways in CAFs whereas TGF-β-TRAP, but not a-PD-1, targeted TGF-β downstream pathways in the epithelial compartment. As mentioned above, the snRNA-Seq data suggested that Tregs may have been reprogrammed transcriptionally and not truly depleted in their cell proportion. A further investigation on the specific intracellular signaling pathways that are modulated by TGF-β-TRAP, but not a-PD-1, may lead to the discovery of better therapeutic targets for Treg reprogramming.

Our bioinformatic analysis with the snRNA-Seq data showed that CCL5, a ligand of CCR5 known to recruit immunosuppressive macrophages, almost disappeared from the KPC-4545 tumors following TGF-β-TRAP treatment. However, the ligands of CCR5 in the TGF-β-TRAP+a-PD-1 combo treatment expanded to CCL7 and CCL8, which may mediate the immune agonist activity of CCR5. Previously, it was shown that TGF-β depletion enhanced the expression of CCL5 in different tumor models (39). Interestingly, following the combo treatment, the CCL5 signaling appeared to be limited to granulocytes. Nevertheless, the differential gene expression analysis showed that TGF-β-TRAP suppresses the granulocyte degranulation and CD4^+^ T cell response to granulocyte degranulation signals, consistent with a published study showing TGF-β reprograms tumor-associated neutrophils (40). These results motivate future studies testing whether targeting neutrophils in combination with TGF-β-TRAP and anti–PD-1 treatment would further suppress CCL5 signaling and degranulation in granulocytes.

Given its numerous roles in cancer progression, TGF-β has been explored as a potential target in oncologic therapy for pancreatic cancer (6, 41). Various agents based on different strategies have been developed and tested preclinically (7). Antisense oligonucleotides, such as the TGF-β2 specific phosphorothioate antisense oligodeoxynucleotide Trabedersen, have been shown to inhibit cancer growth and migration while reversing TGF-β–induced immunosuppression (42). LY364947, a small TGF-βR1 inhibitor, was shown to reprogram intratumor CD8^+^ T cells and Tregs and sensitize tumor cells to ICIs by increasing expression of PD-L1 (43). While LY364947 also shifted the myCAF/iCAF ratio, this effect was transient, and the ratio eventually returned to baseline levels (43). A wide range of neutralizing antibodies have also been tested against PDAC. NIS793 is a monoclonal antibody that blocks TGF-β1/2 ligands and was recently reported to repolarize PDAC tumor cells and increase sensitivity to combination chemotherapy (44). However, no marked effects of NIS793 on CAFs and T cells were observed (44). Our study builds upon this knowledge by using TGF-β-TRAP/AVID-200, a ligand trap that effectively depletes only TGF-β1/3, which are most associated with cancer progression, and not TGF-β2, which is critical in hematopoiesis and cardiac function (6). In addition to demonstrating its efficacy in enhancing ICI, we also utilize snRNA-Seq, which better captures the stromal component, to identify the potential reprogramming of CAFs and myeloid cells by TGF-β-TRAP.

While preclinical targeting of TGF-β in PDAC has shown success, these positive results have yet to be translated to the clinic. Difficulties in bringing these various TGF-β–targeting agents to patients include the pleiotropic effects of TGF-β, which may cause a wide range of side effects from systemic TGF-β inhibition (45). Particularly, TGF-β’s tumor-restraining roles in normal tissue complicate the use of TGF-β–targeting therapies (46). Furthermore, TGF-β inhibition alone is not likely to be effective against PDAC, requiring more complex studies on combination treatment with chemotherapy or immunotherapy (44).

There are multiple ongoing clinical trials for the various agents, most of which are still in the phase I/II stage (41). While some of these agents have been demonstrated to be safe and tolerated, their efficacy has yet to be determined. Notably, galunisertib, a small molecule TGF-βR1 inhibitor, in combination with gemcitabine, improved overall survival compared with gemcitabine alone in first-line treatment for unresectable PDAC (47). However, galunisertib plus PD-L1 blockade showed no significant improvement in survival compared with historical records in second-line treatment of metastatic PDAC (48). In another promising phase III clinical trial (NCT04935359), the efficacy of NIS793 in combination with chemotherapy is currently being assessed for first-line treatment of metastatic PDAC.

Complicating these efforts is the heterogeneity of tumors between different patients, who may respond differently to TGF-β inhibition. A previous preclinical study using pharmacological blockade of TGF-βR2 found a therapeutic effect for PDAC that harbors epithelial loss of TGF-βR2 (49). As such, there is a need to identify biomarkers to determine subsets of patients who may benefit more from TGF-β–targeting therapies. While the finding of KPC-3403 not responding to TGF-β-TRAP in our study is not conclusive of TGF-β-TRAP being less effective against PDAC with lung metastasis potential, it does demonstrate that heterogeneity between tumors may result in different response to TGF-β–targeting therapies.

Clinically, although a majority of patients develop metastatic disease, previous studies have reported differential outcomes depending on the site of organ metastasis, with lung metastasis being associated with better prognosis compared with liver metastasis (50, 51). Therefore, the findings of this study shall be further validated in human PDAC specimens, particularly by comparing liver metastases and lung metastases.

## Methods

### Sex as a biological variable.

Murine experiments in our study were performed with female C57BL/6 mice to limit variability. It is not known whether the findings are relevant to male mice.

### Cell lines.

The original KPC tumor cell line is an established PDAC cell line derived from a KPC transgenic mouse model in a C57BL/6 background with pancreatic tissue–specific Kras and p53 knockin mutations. The KPC-4545 tumor cell line was derived from the primary tumor of a KPC mouse with liver metastases only; and the KPC-3403 tumor cell line was derived from the primary tumor of a KPC mouse with lung metastases only (31).

### Mice.

C57BL/6 mice (6–8 weeks old) mice were purchased from Jackson Laboratory. For survival studies, mice were monitored once a day. Mice with signs of distress including hunched posture, lethargy, dehydration, and rough hair coat were considered to have reached survival endpoint and euthanized.

### Orthotopic model and treatment regimens.

The KPC PDAC orthotopic model has been previously described (52). Briefly, 2 × 10^6^ KPC cells were injected subcutaneously in the flanks of syngeneic female C57BL/6 mice. The subcutaneous tumors were harvested after 1–2 weeks and cut into 2–3 mm^3^ pieces. Such subcutaneous tumors maintain a measurable, discrete mass after implantation into the pancreas, harbor the TME as they spontaneously developed KPC tumors, and remain resistant to the ICI single-agent treatment (32). New 6- to 8-week-old syngeneic female C57BL/6 mice were anesthetized. A left subcostal incision was created to enter the abdomen and access the body and tail of the pancreas. Using microscissors, a 2–3 mm^3^ piece of subcutaneous tumor was implanted within a small pocket in the distal body and tail portion of the pancreas and secured with 7-0 Prolene suture. The peritoneum and skin were sutured in 2 layers with 4-0 sutures.

AVID200 (human surrogate form of TGF-β-TRAP; BMS-986416, Bristol Myers Squibb) and anti-hIgG1f (clone 23H3, Bristol Myers Squibb) were administered intraperitoneally (IP) at 100 μg (5 mg/kg) per dose on days 7, 10, 14, 17, 21, and 24 after orthotopic tumor implantation. mTGF-β-TRAP (mouse surrogate form of TGF-β-TRAP; PPB-5777, Bristol Myers Squibb) and anti-mouse IgG2a isotype control antibodies (Bristol Myers Squibb) were administered IP at 300 μg (15 mg/kg) per dose on days 7, 14, and 21 after orthotopic tumor implantation. Anti-mouse PD-1 G1-D265A antibodies (clone 6A1, Bristol Myers Squibb) and anti-mouse IgG1 isotype control antibodies (clone 5E4, Bristol Myers Squibb) were administered IP at 100 μg (5 mg/kg) per dose on days 7, 10, 14, 17, 21, and 24. Tumor size was measured by Vevo 3100 US in the transverse and longitudinal orientations with respect to the transducer.

### Metastatic model and treatment regimens.

The KPC PDAC liver metastasis model via the hemispleen technique was previously described (53). In brief, 6- to 8-week-old syngeneic female C57BL/6 mice were anesthetized. After creation of a left subcostal incision, the spleen was eviscerated, clipped, and divided in half. A total of 2 × 10^5^ KPC cells in 100 μL PBS (Gibco) were injected into half of the spleen and then flushed with 150 μL PBS in the same syringe. The splenic vessels were subsequently clipped, and the injected hemispleen was subsequently resected to remove residual tumor cells. The peritoneum and skin were sutured in 2 layers with 4-0 sutures.

mTGF-β-TRAP (mouse surrogate form of TGF-β-TRAP; PPB-5777, Bristol Myers Squibb) and anti-mouse IgG2a isotype control antibodies (Bristol Myers Squibb) were administered IP at 300 μg (15 mg/kg) per dose on days 4, 11, and 18 after hemispleen surgery. Anti-mouse PD-1 G1-D265A antibodies (clone 6A1, PPB-3808, Bristol Myers Squibb) and anti-mouse IgG1 isotype control antibodies (clone 5E4, Bristol Myers Squibb) were administered IP at 100 μg (5 mg/kg) per dose on days 4, 7, 11, 14, 18, and 21.

### Cell cultures.

These KPC PDAC cell lines were cultured in RPMI 1640 medium (Life Technologies) with 10% fetal bovine serum (FBS, Benchmark), 1% minimum essential medium nonessential amino acids (MEM-NEAA; Life Technologies), 1% penicillin/streptomycin (Life Technologies), 1% sodium pyruvate (MilliporeSigma), and 1% l-glutamine (Life Technologies), maintained at 37°C in 5% CO_2_. Harvested tumor-infiltrating immune cells were processed in T cell media consisting of RPMI 1640 with 10% FBS, 1% MEM-NEAA, 1% penicillin/streptomycin, 1% l-glutamine, and 0.05% 2-mercaptoethanol (MilliporeSigma).

### Cell processing and flow cytometry.

Murine orthotopic pancreatic tumors were resected on day 13 after tumor implantation for analysis of tumor-infiltrating immune cells as previously described (54). Each tumor was mechanically and enzymatically processed using the mouse Tumor Dissociation Kit (Miltenyi Biotec) and gentleMACS Octo Dissociator (Miltenyi Biotec), filtered through a 70 μm strainer (CELLTREAT), and brought to a volume of 20 mL in T cell medium. Cell suspensions were centrifuged at 1,500 rpm for 5 minutes. Cell pellets were suspended in 4 mL of Ammonium-Chloride-Potassium lysis buffer (Quality Biological) and spun at 1,500 rpm for 5 minutes. Cell pellets were then resuspended in 6 mL of 80% Percoll (Cytiva), overlaid with 6 mL of 40% Percoll, and centrifuged for 25 minutes at 3,200 rpm without brake at room temperature. The leukocyte layer was removed and quenched with T cell media.

Following isolation of tumor-infiltrating immune cells from murine pancreatic tumor, cells were stained with Live/Dead Aqua (Invitrogen L34957) for 30 minutes on ice, washed twice with PBS, and then blocked with anti-mouse Fc antibody (BD Biosciences) in FACS buffer for 10 minutes on ice. The cell surface antibodies used were CD45-PerCP Cy5.5 (BioLegend 30-F11), CD8-PE Cy7 (BioLegend 53-6.7), CD4-APC Fire (BioLegend GK1.5), CD25-BV421 (BioLegend PC61), CD45-FITC (BioLegend 30-F11), CD3-APC Cy7 (BioLegend 17A2), CD4-BV650 (BioLegend RM4-5), CD11b-PE Texas Red (Thermo Fisher Scientific M1/70.15), Ly6C-PerCP Cy5.5 (BioLegend HK1.4), Ly6G-V450 (BD Biosciences 1A8), F4/80-PE Cy7 (BioLegend BM8), OX40-FITC (BioLegend Ber-ACT35), LAG3-PE (BioLegend C9B7W), TIM3-PE CF594 (BD Biosciences 5D12), CD137-APC (BioLegend 17B5), and PD-1-BV421 (BioLegend 29F.1A12).

Intracellular staining for FoxP3 was performed following cell surface marker incubation. Cells were suspended in cold Fixation/Permeabilization solution (eBioscience) and incubated for 30 minutes on ice at 4°C. Cells were then washed twice with Permeabilization/Wash buffer (eBioscience). FoxP3-PE antibody (BioLegend 150D) was added, and the cells were incubated for 40 minutes on ice. Cells were then washed twice in Permeabilization/Wash buffer and resuspended in FACS buffer. All flow cytometry experiments were performed using CytoFLEX (Beckman Coulter), and flow data were analyzed using CytExpert software (Beckman Coulter).

### Intracellular staining for IFN-γ and flow cytometry.

Isolation of tumor-infiltrating immune cells from murine pancreatic tumors was performed on day 13 after tumor implantation surgery as described above. Tumor-infiltrating immune cells of each mouse were enriched for CD8^+^ T cells by negative isolation per the EasySep Mouse CD8^+^ T Cell Isolation Kit (STEMCELL Technologies). Isolated CD8^+^ T cells were incubated with CD3/CD28 stimulation beads using the Dynabeads Mouse T-Activator CD3/CD28 Kit (Life Technologies) according to the manufacturer’s protocol at 1:1 cell-to-bead ratio in T cell media for 15 hours at 37°C in 5% CO_2_. GolgiPlug (BD Biosciences) was added subsequently at 1:1,000 volume ratio, and the cells were incubated for an additional 5 hours at 37°C in 5% CO_2_. After a total incubation time of 20 hours, the beads were removed per manufacturer’s protocol and washed twice with PBS. The cells from mice in the same treatment group were pooled and resuspended in PBS. Triplicates of this pooled cell suspension were placed into wells of a 96-well plate at a volume of 100 μL per well. Cells were stained with Live/Dead Aqua (Invitrogen), CD8-PE Cy7 (BioLegend 53-6.7), and CD3-APC Cy7 (BioLegend 17A2) as described above.

Intracellular staining for IFN-γ was performed following cell surface marker incubation using the BD Cytofix/Cytoperm Fixation/Permeabilization Kit. Cells were suspended in cold Fixation/Permeabilization buffer (BD Biosciences) and incubated for 30 minutes on ice at 4°C. Cells were then washed twice with Permeabilization/Wash buffer (BD Biosciences). IFN-γ-BV421 antibody (BioLegend XMG1.2) was added, and the cells were incubated for 30 minutes on ice. Cells were then washed twice in Permeabilization/Wash buffer and resuspended in FACS buffer prior to flow cytometry.

### snRNA-Seq nuclei isolation.

Frozen mouse orthotopic KPC PDAC tumors were homogenized and isolated into single nuclei, modified from a previously described 10x Genomics protocol (55). Briefly, fresh sucrose density buffer containing sucrose (0.344 g/mL, MilliporeSigma), 10 mM HEPES (Gibco), 5 mM CaCl_2_ (MilliporeSigma), 3 mM magnesium acetate tetrahydrate (MilliporeSigma), 1 mM DTT (MilliporeSigma), and 0.2 U/μL NxGen RNase inhibitor (Lucigen) was prepared. Frozen mouse orthotopic KPC PDAC tumors were mechanically homogenized into powder form using mortar and pestle in liquid nitrogen. Lysis buffer containing 0.1% Triton X-100 (MilliporeSigma), 0.2 U/μL NxGen RNase inhibitor, and sucrose density buffer was added to the homogenized tissue and allowed to sit for 3 minutes on ice. Following lysis, the crude homogenate was pipetted over a 100 μm cell strainer, washed with sucrose density buffer, and centrifuged at 400 rcf for 5 minutes at 4°C on low-brake setting. The supernatant was aspirated, and the pellet was resuspended in resuspension buffer containing PBS + 1% BSA (MilliporeSigma) and 0.2 U/μL RNase inhibitor over a 35 μm filter flow tube. Amplified cDNA was generated from RNA from each single nucleus using the 10x Genomics Chromium Next GEM Single Cell V(D)J Reagent Kits v.1.1, per the manufacturer’s instructions (56).

### snRNA-Seq processing and analysis.

Our snRNA-Seq data included 2 batches of data from both KPC-4545 and KPC-3403 tumor models, respectively. Each batch consisted of 6 samples: 1) isotype controls, 2) mTGF-β-TRAP, 3) a-PD-1, 4) anti–IL-1β, 5) mTGF-β-TRAP+a-PD-1, and 6) anti–IL-1β+a-PD-1. Samples including anti–IL-1β (samples 4 and 6) were included in initial clustering as part of a related study, but downstream analysis was only performed on samples 1, 2, 3, and 5 in regard to this manuscript testing the efficacy of TGF-β-TRAP in combination with a-PD-1. BCL files were converted to FASTQ format using Illumina bcl2fastq software. CellRanger v6.1.2 (10x Genomics) was used to demultiplex the FASTQ reads and align them to the GRCm38-mm10 transcriptome reference. The “include-intron” option was included to account for intronic reads. Unique molecular identifiers and nuclei barcodes were extracted, outputting a digital gene expression matrix for each sample. CellRanger output showed fraction reads in cells, defined as the fraction of reads with valid barcodes that are confidently mapped and associated with a cell, ranging from 15.3% to 47.1% across all the TGF-β-TRAP samples (Supplemental Table 19). Similarly, CellRanger output showed reads mapped confidently to transcriptome, defined as reads mapped to a unique gene in the transcriptome, ranging from 4.9% to 32.9% among all samples (Supplemental Table 19). Downstream analysis such as identification of highly variable genes, dimensional reduction, unsupervised clustering, and differential gene expression was performed with Seurat v4.1.0 (57).

CellBender remove-background was run on Terra workspace with default parameters to remove ambient RNA and other technical artifacts to enhance marker specificity (58). Cells from all 6 samples of each batch were subsequently merged into a single object via the Seurat merge function. Nuclei with fewer than 200 features and more than 2,500 features were filtered out. Genes *Gm42418* and *AY036618* were removed from subsequent snRNA-Seq analysis given their overlap with rRNA element Rn45s and may possibly represent rRNA contamination (59). Counts were log-normalized with the NormalizeData function from Seurat by a scale factor of 10,000. The 2,000 most variable genes were identified and used to perform principal component analysis. Nuclei were clustered with the FindClusters function at a resolution of 0.8, and the dimensionally reduced data were represented through UMAP (60). Clusters identified from the previous step were annotated using known cell type–specific genes found in the literature (25, 35, 61, 62). No substantial batch effects between samples were observed. The 4 samples pertaining to this TGF-β-TRAP study were then extracted for further downstream analysis. This process was performed twice, once for KPC-4545 batch and once for KPC-3403 batch.

Differential gene expression of the same cell type between samples of different treatments was carried out with the FindMarkers function and the MAST test on Seurat (63). A threshold of log_2_FC ≥ 0.5 or log_2_FC ≤ –0.5 and adjusted *P* < 0.05 was used to determine significantly differentiated genes, which were then used for functional pathway analysis with Metascape (64). Pathways with an FDR-adjusted *P* value of less than 0.05 were deemed significant.

Mouse genes were converted to their human homologs, and iTALK v0.1.0 was used to estimate ligand-receptor interactions between CAFs, macrophages, granulocytes, and CD8^+^ T cells. Given our interest in interaction between different stromal and immune cells, interactions between different clusters of CAFs were excluded. Ligand-receptor interactions were inferred separately for each sample (65). Ligand-receptor pairs were visualized according to the 4 groups set by iTALK, including growth factor, cytokines, checkpoint, and others. The top 30 ligand-receptor pairs for each group were visualized and contrasted between different samples.

### Statistics.

All statistical analyses and graphing were performed using GraphPad Prism 9 software. For comparison of cell number and cell percentage, the mean values were analyzed using the unpaired 2-tailed *t* test with Holm-Sidak correction method. Kaplan-Meier survival analysis were performed using the log-rank tests to estimate median survival and analyze survival outcomes among subgroups. A *P* < 0.05 was considered statistically significant.

### Study approval.

All mice were maintained in accordance with and with approval from the Johns Hopkins University Institutional Animal Care and Use Committee.

### Data availability.

Mouse snRNA-Seq data are available in the NCBI’s Gene Expression Omnibus database (GSE275596). Values for all data points in graphs are reported in the Supporting Data Values file. All code is available at the following repository: https://github.com/hkung2/PDAC-TGFB-TRAP-Manuscript; commit ID 21946af.

All data needed to evaluate the conclusions in the paper are present in the paper and the supplement.

## Author contributions

SYC, WRB, WLH, and LZ designed research studies. SYC, HCK, BE, IW, JW, HZ, ML, JF, RW, and KC conducted experiments. SYC, HCK, and BE acquired data. SYC, HCK, and BE analyzed data. BE and IW provided reagents. JW, HZ, ML, and JF were responsible for developing and providing mouse models. SYC, HCK, and LZ were responsible for writing the manuscript. SYC, HCK, WLH, and LZ were responsible for reviewing the manuscript. MP, WRB, WLH, and LZ were responsible for project supervision. LZ and MP were responsible for project administration. SYC conducted the experiments, analyzed the data, and wrote the manuscript, so she is first coauthor. HCK performed bioinformatic analysis, wrote the manuscript, and organized figures, so he is second coauthor. BE performed experiments and performed data analysis, so she is third coauthor.

## Supplementary Material

Supplemental data

Supporting data values

## Version 1 09/19/2024

In-Press Preview

## Version 2 11/08/2024

Electronic publication
