# Supplementary material for: Targeting heterogeneous tumor microenvironments in pancreatic cancer mouse models of metastasis by TGF-β depletion
Source: JCI Insight. 2024 Nov 8;9(21):e182766. doi: 10.1172/jci.insight.182766 (PMC11610749; doi:10.1172/jci.insight.182766)
Supplement: Supplemental data [file jciinsight-9-182766-s008.pdf]

<u>**Supplemental Materials**</u>

Contents

Supplementary Figures

- **Supplementary Figure 1 (Fig.S1): The combination of TGFβ-TRAP and a-PD-1 had comparable rates of tumor growth to a-PD-1 monotherapy in a PDAC orthotopic mouse model with KPC-3403 cell line.**
- **Supplementary Figure 2 (Fig.S2): Treatment schema for PDAC orthotopic model tumor growth experiment using mTGFβ-TRAP, the mouse surrogate form of TGFβ-TRAP.**
- **Supplementary Figure 3 (Fig.S3): Orthotopic tumor volumes at the time of necropsy were comparable among different treatment groups of mTGFβ-TRAP and a-PD-1.**
- **Supplementary Figure 4 (Fig.S4): Flow cytometry gating strategy for identification of effector T-cells**.
- **Supplementary Figure 5 (Fig.S5): The combination of mTGFβ-TRAP and a-PD-1 had comparable levels of isolated tumor-infiltrating CD4+ effector cells, regulatory T-cells, M-MDSC cells, and CD8+IFN𝛾+ cells for KPC-4545 and KPC-3403 orthotopic tumors.**
- **Supplementary Figure 6 (Fig.S6): Flow cytometry gating strategy for identification of regulatory T-cells**.
- **Supplementary Figure 7 (Fig.S7): Flow cytometry gating strategy for identification of IFN𝛾+ cells.**
- **Supplementary Figure 8 (Fig.S8): Flow cytometry gating strategy for identification of myeloid cell subtypes.**
- **Supplementary Figure 9 (Fig.S9): TGFβ pathway gene expression after treatment in tumor epithelial cells and CAFs in KPC-4545 tumor.**
- **Supplementary Figure 10 (Fig.S10): TGFβ pathway and CAF phenotype after treatment in the KPC-4545 orthotopic tumor.**
- **Supplemental Figure 11 (Fig.S11): *Thsd4* is highly expressed in CAF subcluster s5**
- **Supplementary Figure 12 (Fig.S12): CD8+ T-Cell Exhaustion Panel Across Samples.**
- **Supplementary Figure 13 (Fig.S13): Single-nuclear RNA-seq analysis of differentially expressed genes between different treatment groups.**
- **Supplementary Figure 14 (Fig.S14): Growth factor and other ligand-receptor analysis between CAFs and immune cells.**
- **Supplementary Figure 15 (Fig.S15): CAF phenotype of the KPC-3403 tumor.**
- **Supplementary Figure 16 (Fig.S16): Comparison of the immune landscape between KPC-4545 and KPC-3403 model.**

Supplementary Tables

- **Supplementary Table 1 (Table.S1): Cancer Associated Fibroblast Major and Subcluster Distribution**
- **Supplementary Table 2 (Table.S2): Top 50 Differentially Expressed Genes of Each Cancer Associated Fibroblast Subcluster**

- **Supplementary Table 3 (Table.S3): Top 30 Growth Factor Interactions in Control Sample**
- **Supplementary Table 4 (Table.S4): Top 30 Growth Factor Interactions in TGFβ-TRAP Sample**
- **Supplementary Table 5 (Table.S5): Top 30 Cytokine Interactions in Control Sample**
- **Supplementary Table 6 (Table.S6): Top 30 Cytokine Interactions in TGFβ-TRAP Sample**
- **Supplementary Table 7 (Table.S7): Top 30 Checkpoint Interactions in Control Sample**
- **Supplementary Table 8 (Table.S8): Top 30 Checkpoint Interactions in TGFβ-TRAP Sample**
- **Supplementary Table 9 (Table.S9): Top 30 Other Interactions in Control Sample**
- **Supplementary Table 10 (Table.S10): Top 30 Other Interactions in TGFβ-TRAP Sample**
- **Supplementary Table 11 (Table.S11): Top 30 Growth Factor Interactions in anti-PD-1 Sample**
- **Supplementary Table 12 (Table.S12): Top 30 Growth Factor Interactions in Combo (anti-PD-1+ TGFβ-TRAP) Sample**
- **Supplementary Table 13 (Table.S13): Top 30 Cytokines Interactions in anti-PD-1 Sample**
- **Supplementary Table 14 (Table.S14): Top 30 Cytokines Interactions in Combo (anti-PD-1+ TGFβ-TRAP) Sample**
- **Supplementary Table 15 (Table.S15): Top 30 Checkpoint Interactions in anti-PD-1 Sample**
- **Supplementary Table 16 (Table.S16): Top 30 Checkpoint Interactions in Combo (anti-PD-1+ TGFβ-TRAP) Sample**
- **Supplementary Table 17 (Table.S17): Top 30 Other Interactions in anti-PD-1 Sample**
- **Supplementary Table 18 (Table.S18): Top 30 Other Interactions in Combo (anti-PD-1+ TGFβ-TRAP) Sample**
- **Supplementary Table 19 (Table.S19): Sample Quality Metrics Output from CellRanger for KPC-4545 and KPC-3403**

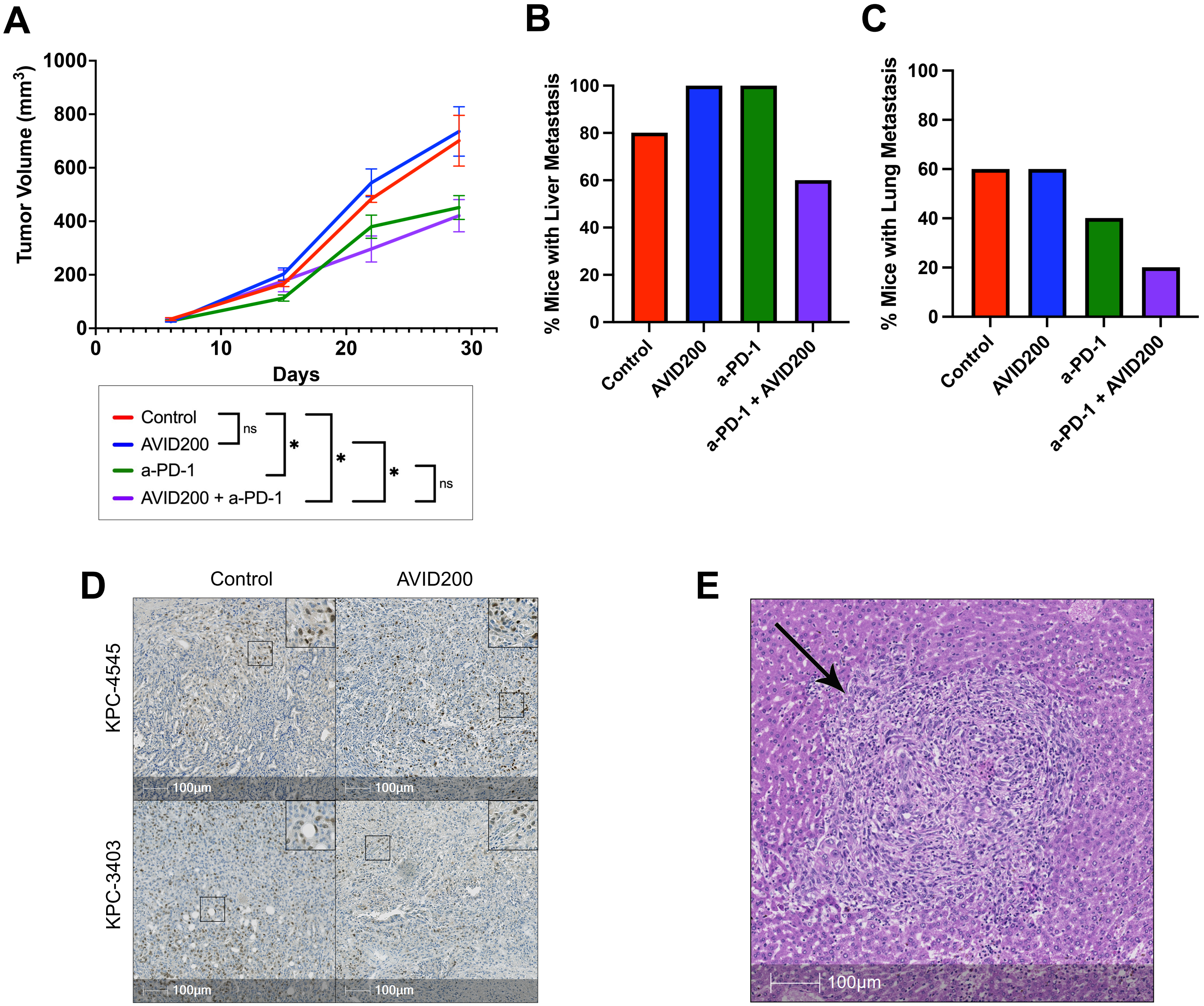

Supplemental Figure 1. The combination of TGFβ-TRAP and a-PD-1 had comparable rates of tumor growth to a-PD-1 monotherapy in a PDAC orthotopic mouse model with KPC-3403 cell line. (A) PDAC orthotopic tumor size was evaluated by ultrasound weekly until day 29 in mice treated with different combinations of AVID200 and a-PD-1 (n = 5 mice per group). **(B and C)** When PDAC orthotopic tumor mice reached survival endpoint, necropsies were performed, and the numbers of mice with liver metastases for the KPC-4545 cell line **(B)** and lung metastases for the KPC-3403 cell line **(C)** were identified grossly and histologically (n = 5 mice per group). No significant difference was observed between groups by Fisher's exact test, which was used to compare metastasis rates and treatment group; if not indicated, there is no

significance. **(D)** Representative images of Ki-67 immunohistochemistry staining in the Control and AVID200 groups for KPC-4545 and KPC-3403. **(E)** Representative H&E of liver metastasis established in the KPC-4545 hemispleen model. Unpaired t-test was used to compare day 29 tumor volumes between two treatment groups. Chi-squared test was used to examine the correlation between treatment groups and metastasis rates. *, $p<0.05$; **, $p<0.01$; ***, $p<0.001$.

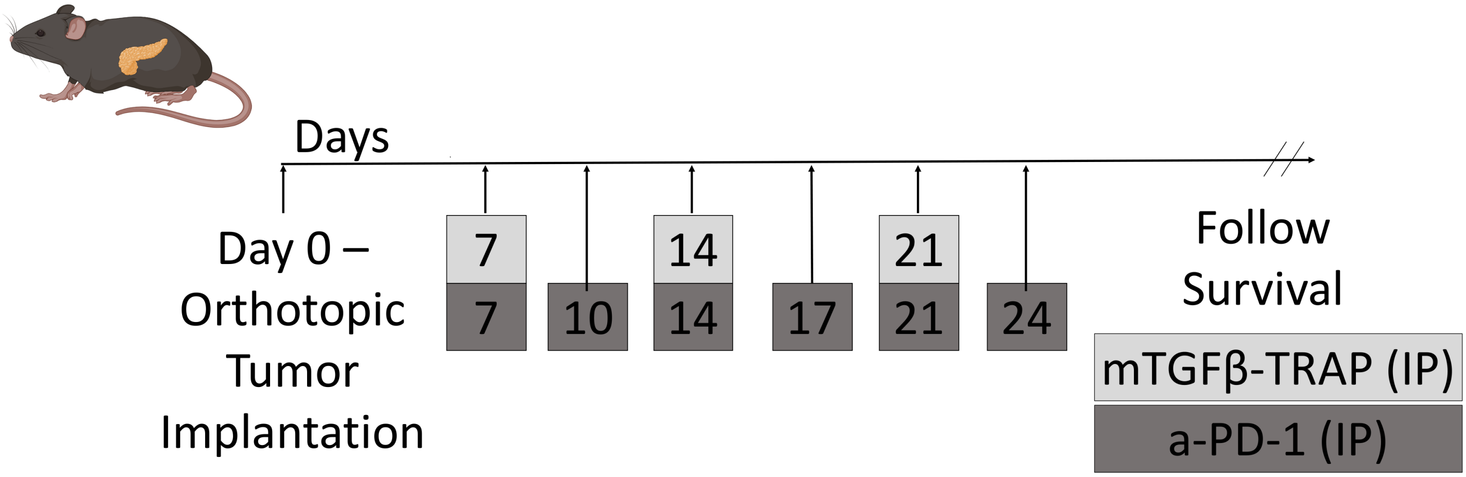

Supplemental Figure 2. Treatment schema for PDAC orthotopic model tumor growth experiment using mTGFβ-TRAP, the mouse surrogate form of TGFβ-TRAP. On day 0, subcutaneous tumors formed by injecting KPC-4545 or KPC-3403 tumor cells onto syngeneic wild-type C57Bl/6 mice approximately 2 weeks prior were resected and divided into cubes of 2-3 mm diameter. Each cube of tumor was then implanted orthotopically into the pancreas of a syngeneic wild-type C57Bl/6 mouse. Following orthotopic tumor implantation, mice were randomized into different treatment groups as indicated (n = 9 to 10 mice per group). Tumor-bearing mice were treated with mTGFβ-TRAP (15 mg/kg i.p. once weekly for three weeks), a-PD-1 (5 mg/kg i.p. twice weekly for three weeks), or IgG control (5 mg/kg i.p. twice weekly for three weeks) on days indicated. Mice were followed for survival.

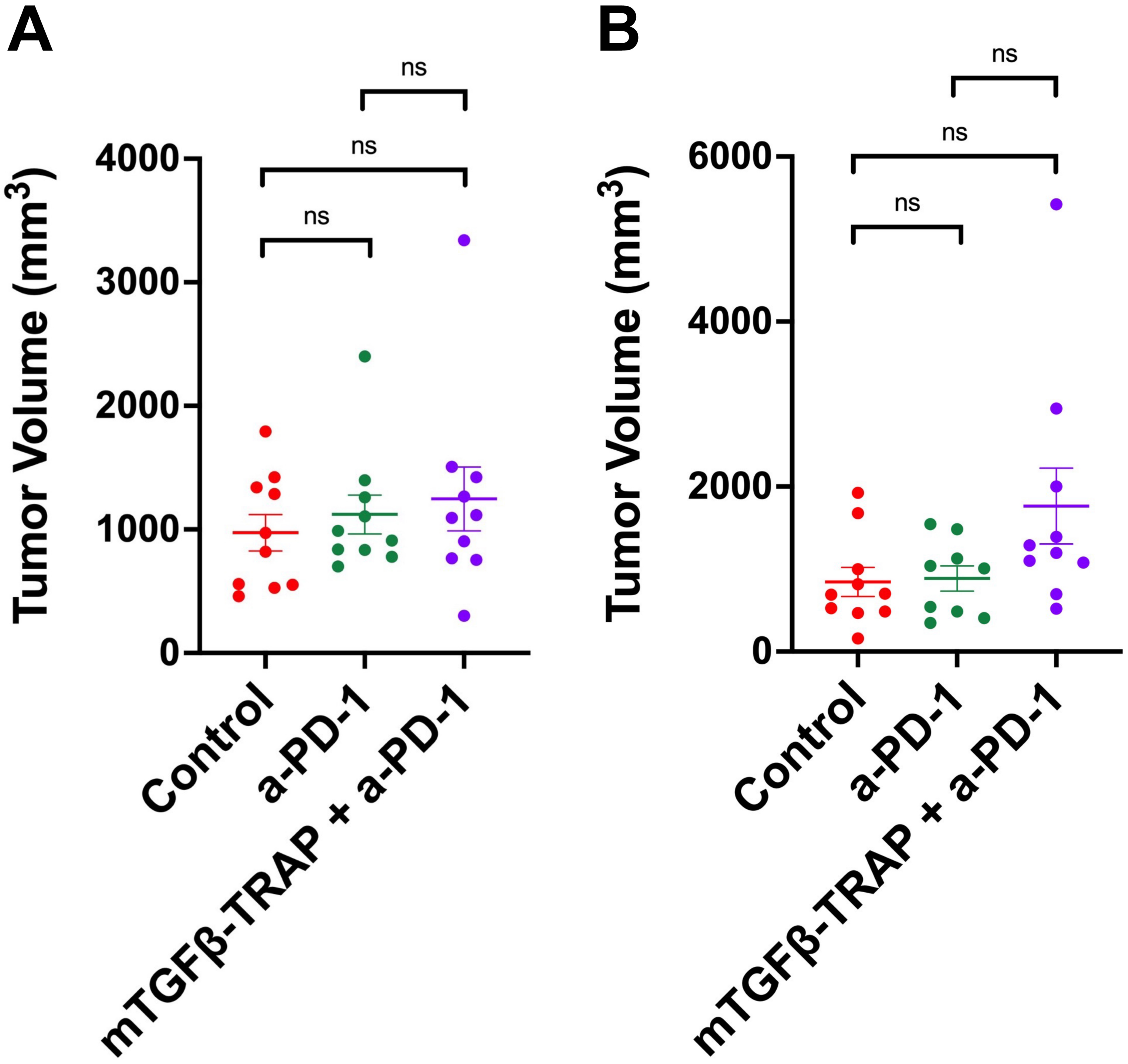

Supplemental Figure 3. Orthotopic tumor volumes at the time of necropsy were comparable among different treatment groups of mTGFβ-TRAP and a-PD-1. (A-B) Orthotopic tumor volumes measured by caliper at the time of necropsy for KPC-4545 **(A)** and KPC-3403 **(B)** orthotopic PDAC mice (n = 10 mice per group). Data represent mean ± SEM. ns, non-significant, by unpaired t-test.

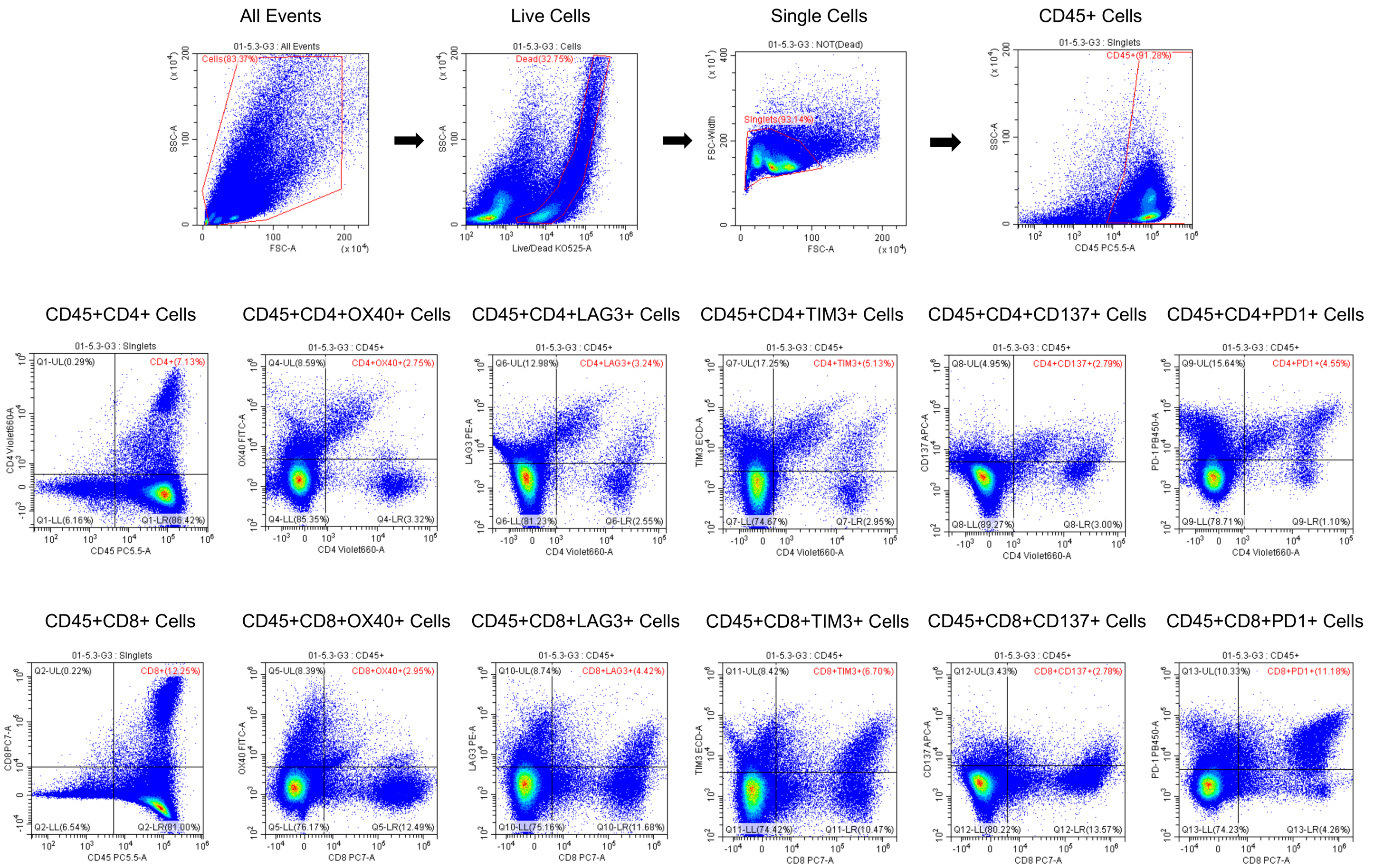

Supplemental Figure 4. Flow cytometry gating strategy for identification of effector T-cells. Dead cells were excluded by gating on cells negative for viability marker Aqua Blue. Forward scatter width (FSC-W) and scatter area (FSC-A) were used to exclude doublets. CD45+ T-cells were identified. The flow cytometry gating strategy of different effector T-cell types are shown.

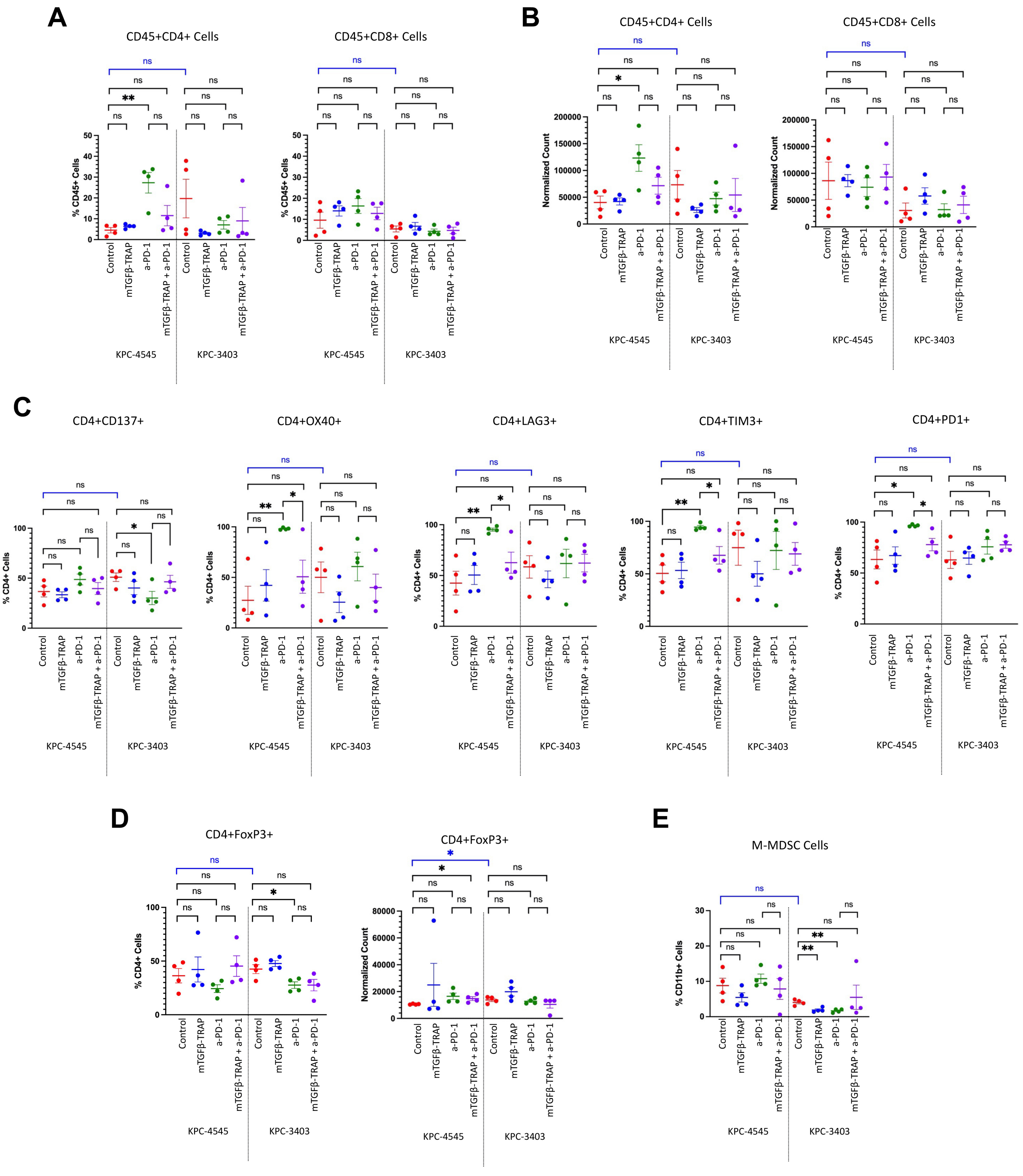

Supplemental Figure 5. The combination of mTGFβ-TRAP and a-PD-1 had comparable levels of isolated tumor-infiltrating CD4+ effector cells, regulatory T-cells, M-MDSC cells, and CD8+IFN*γ*+ cells for KPC-4545 and KPC-3403 orthotopic tumors. (A-E) Flow

cytometry was performed on isolated tumor-infiltrating immune cells from resected orthotopic tumor on day 13 (data in A, B, C, and D are from one experiment, data in E are from a separate experiment). The following isolated tumor-infiltrating immune cells were analyzed: **(A)** percentage of CD4+ and CD8+ cells among CD45+ cells (n = 4 mice per group), **(B)** normalized cell counts of CD4+ and CD8+ cells per 1 x 10^6 cells (n = 4 mice per group), **(C)** percentage of CD137+, OX40+, LAG3+, TIM3+, and PD1+ cells among CD45+CD4+ cells (n = 4 mice per group), **(D)** percentage of FoxP3+ cells among CD4+ cells (n = 4 mice per group), normalized cell counts of CD4+FoxP3+ per 1 x 10^6 cells (n = 4 mice per group), **(E)** percentage of M-MDSC cells among CD45+CD11b+ cells (n = 4 mice per group), *, $p<0.05$; **, $p<0.01$; ***, $p<0.001$, by unpaired t-test. Triplicate measurement; Data represent mean ± SEM.

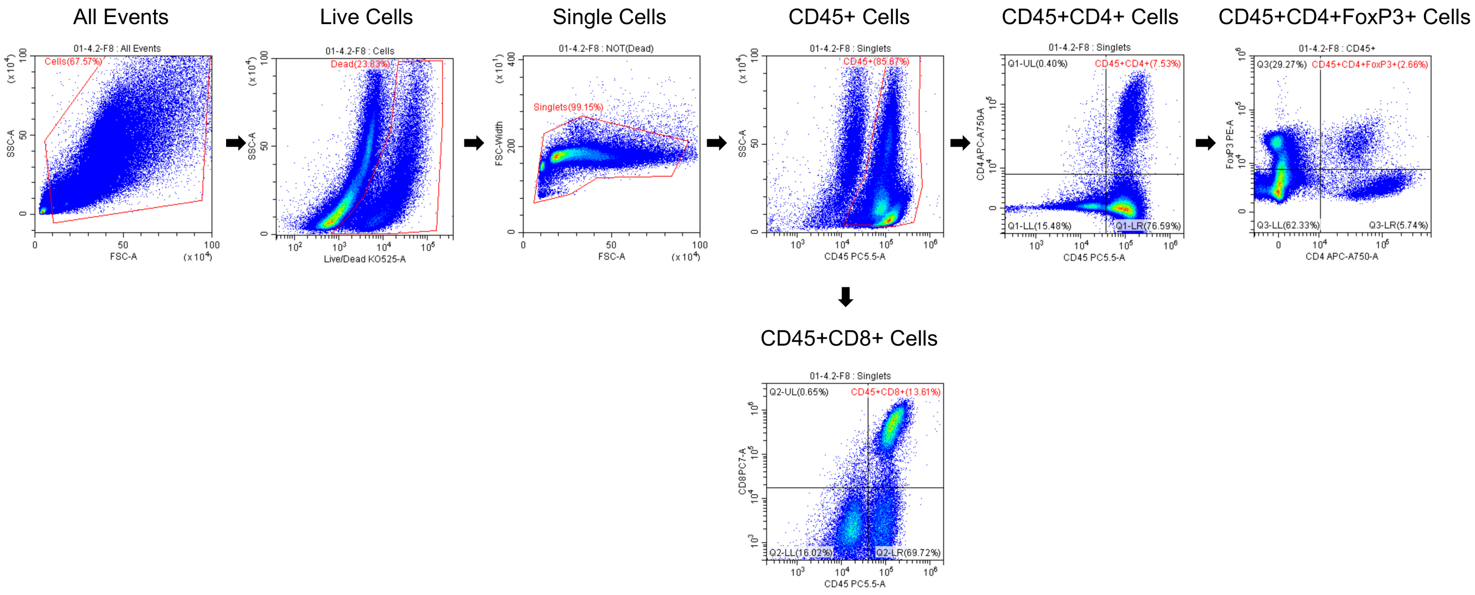

Supplemental Figure 6. Flow cytometry gating strategy for identification of regulatory T-cells. Dead cells were excluded by gating on cells negative for viability marker Aqua Blue. Forward scatter width (FSC-W) and scatter area (FSC-A) were used to exclude doublets. CD45+ cells were identified. Regulatory T-cells were defined as CD45+CD4+FoxP3+ cells.

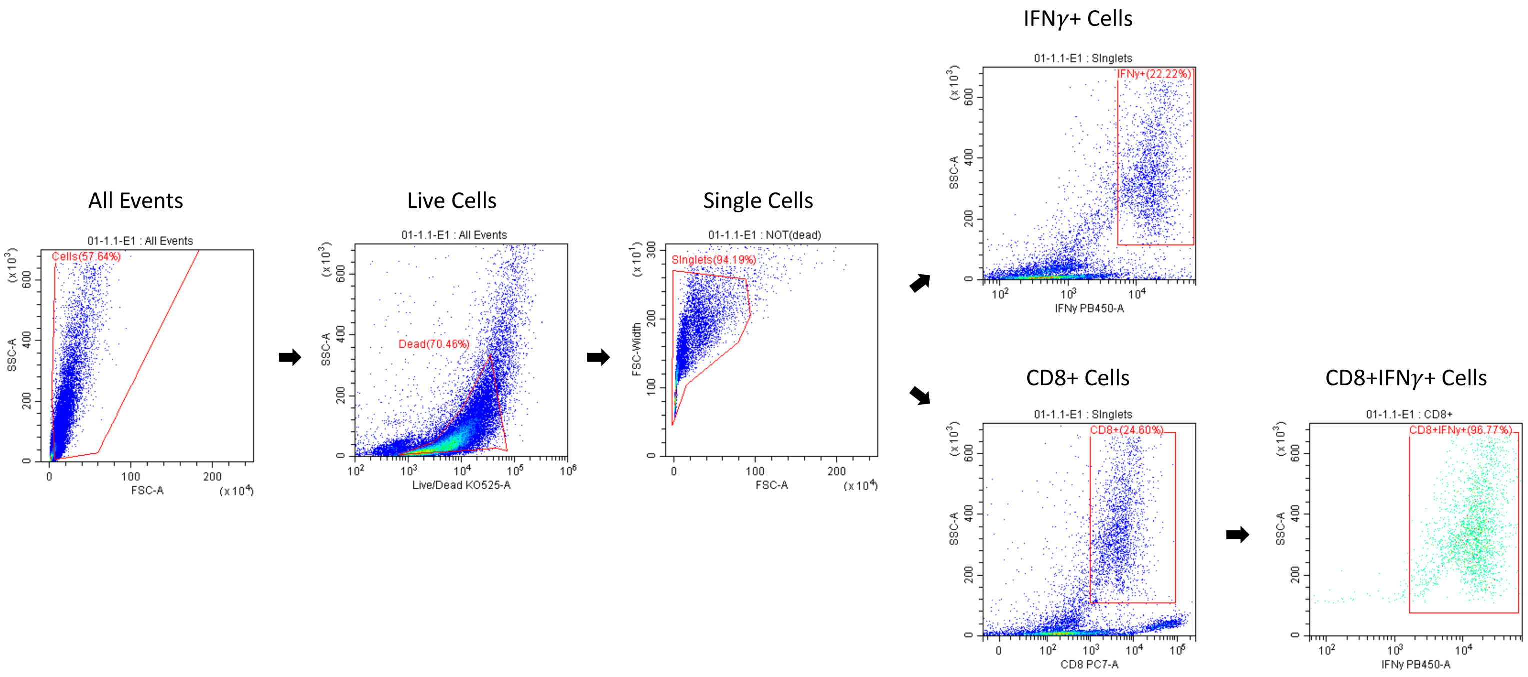

Supplemental Figure 7. Flow cytometry gating strategy for identification of IFNγ+ cells. Dead cells were excluded by gating on cells negative for viability marker Aqua Blue. Forward scatter width (FSC-W) and scatter area (FSC-A) were used to exclude doublets. IFNγ+ cells (CD8+ and CD8- cells) were identified with the gating strategy shown**.**

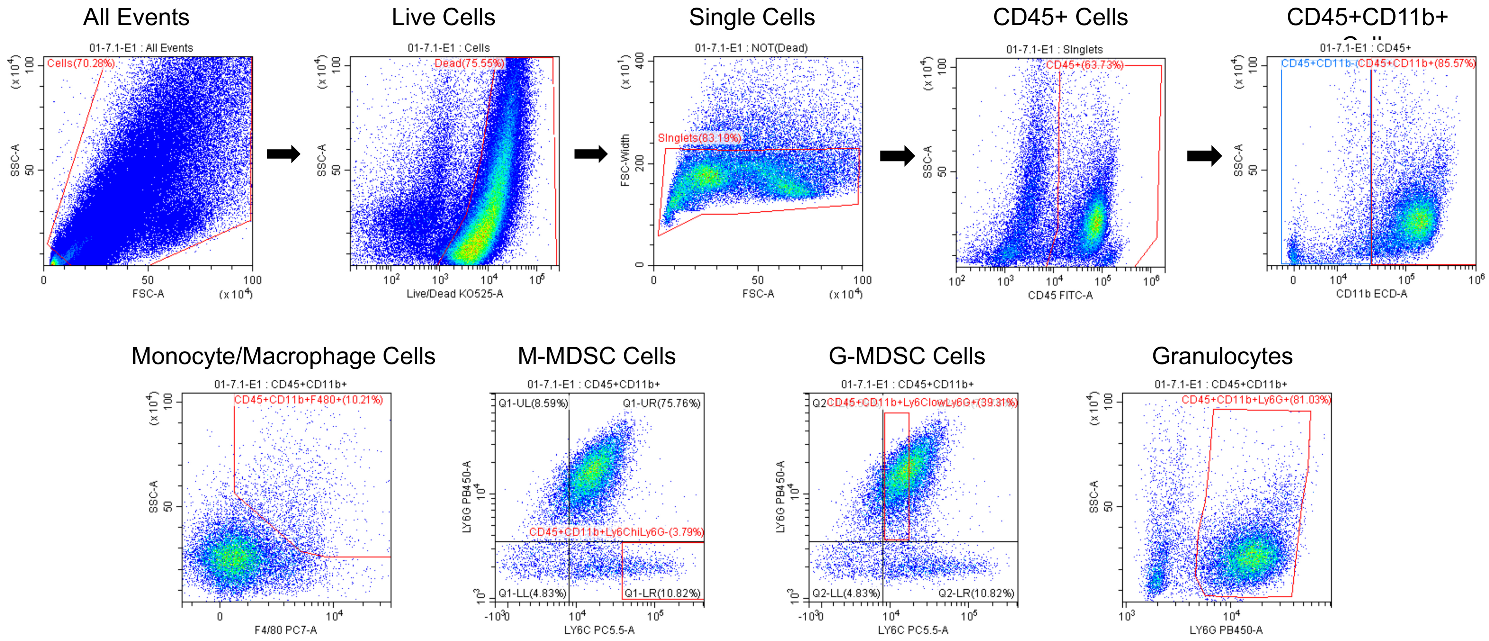

Supplemental Figure 8. Flow cytometry gating strategy for identification of myeloid cell subtypes. Dead cells were excluded by gating on cells negative for viability marker Aqua Blue. Forward scatter width (FSC-W) and scatter area (FSC-A) were used to exclude doublets. CD45+CD11b+ cells were identified. Monocyte/macrophage cells were defined as CD45+CD11b+F4/80+ cells. M-MDSC cells were defined as CD45+CD11b+Ly6C^{high}Ly6G- cells. G-GMDSC cells were defined as CD45+CD11b+Ly6C^{low}Ly6G+ cells. Granulocytes were defined as CD45+CD11b+Ly6G+ cells.

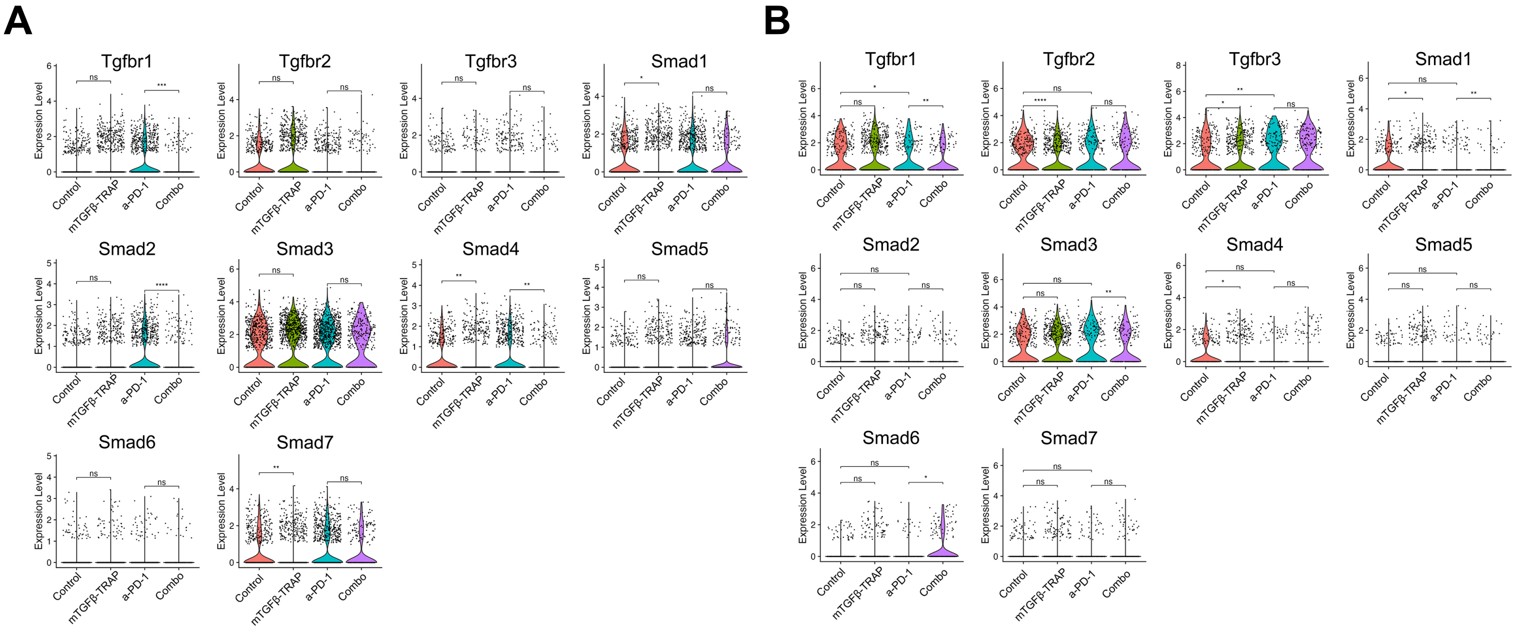

Supplemental Figure 9. TGFβ pathway gene expression after treatment in tumor epithelial cells and CAFs in KPC-4545 tumor. (A&B) Violin plot of normalized gene expression of TGFβ downstream target genes across samples in all tumor epithelial cells **(A)** and CAFs **(B)**. Combo represents a-PD-1+mTGFβ-TRAP.

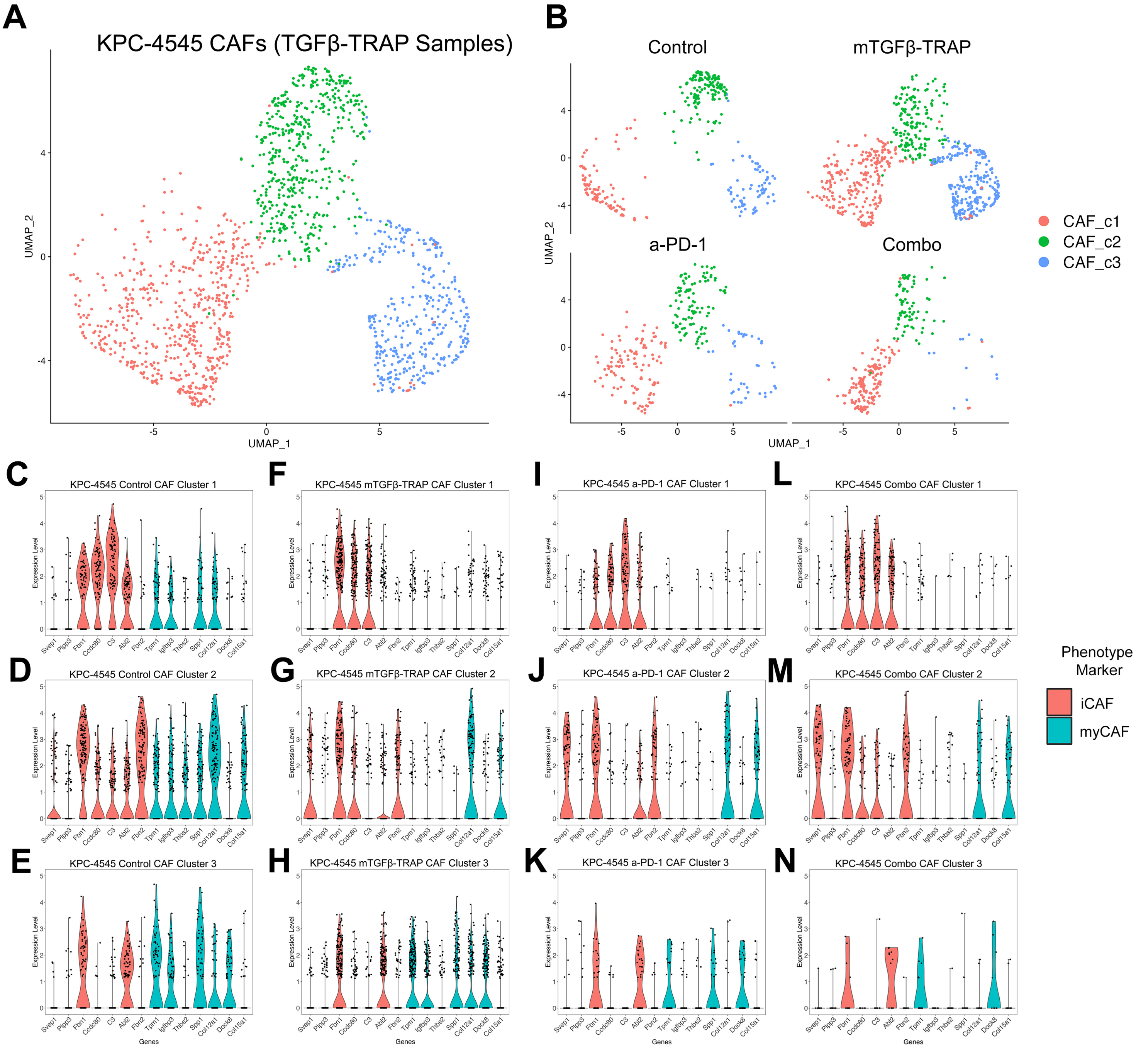

Supplemental Figure 10. Cancer associated fibroblast phenotype after treatment in the KPC-4545 orthotopic tumor. (A, B) Uniform manifold approximation and projection (UMAP) embedding of re-clustered cancer associated fibroblasts (CAFs) labeled as the three major clusters across the four TGFβ-TRAP samples combined **(A)** and separated by treatments **(B)**. **(C-N)** KPC-4545 CAF phenotype gene panel with iCAF (red) and myCAF (blue) signature genes across the three major CAF clusters in control **(C-E)**, TGFβ-TRAP treated **(F-H)**, a-PD-1 treated

(I-K), and a-PD-1 + TGFβ-TRAP treated samples **(L-N)**. Combo represents a-PD-1 + mTGFβ-TRAP. *, $p<0.05$ by Mann-Whitney U-test

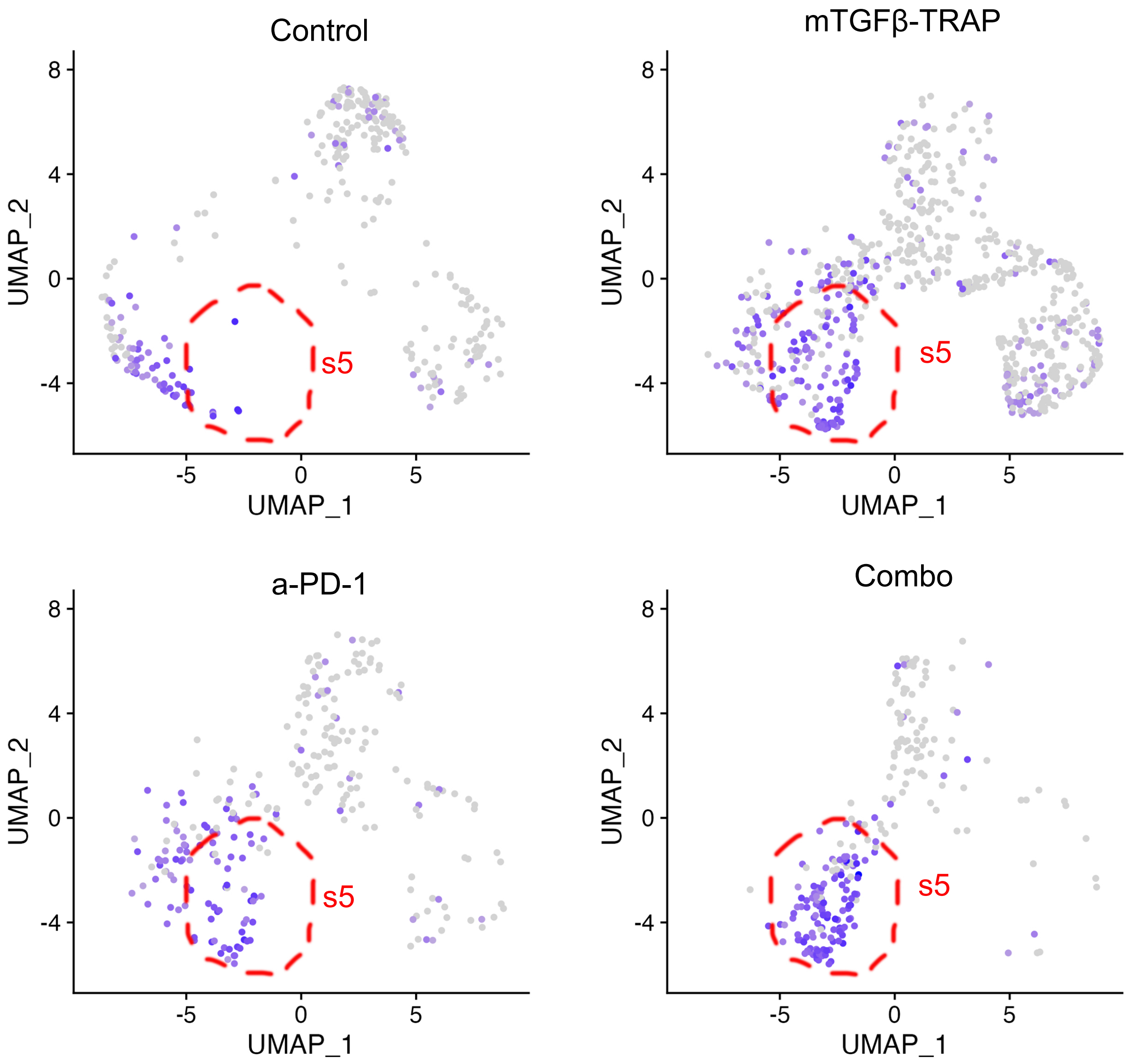

Supplemental Figure 11. *Thsd4* is highly expressed in CAF subcluster s5. Normalized expression of *Thsd4* in CAFs across TGFβ-TRAP samples. CAF subcluster s5 is circled in red. Combo represents a-PD-1 + mTGFβ-TRAP.

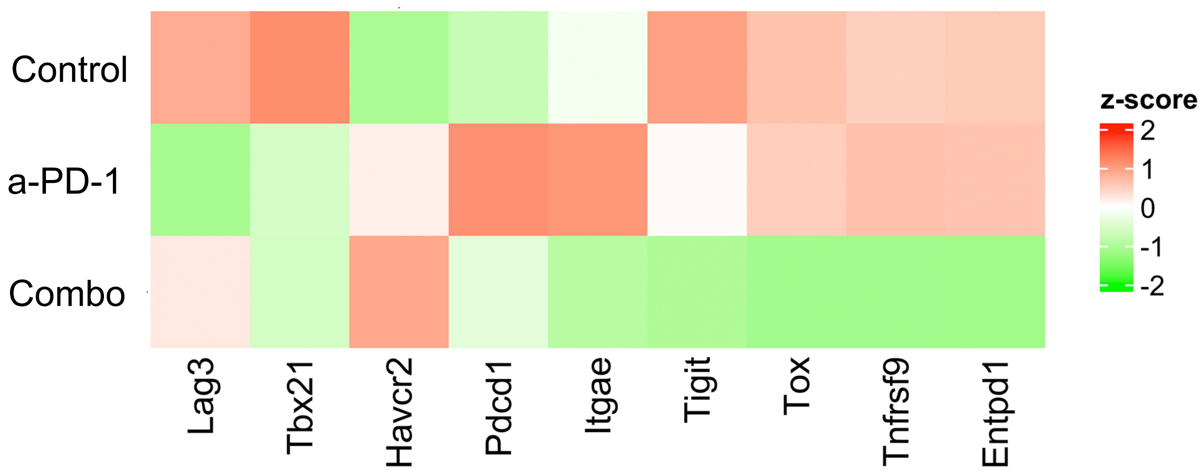

Supplemental Figure 12. CD8+ T-Cell Exhaustion Panel Across Samples. (A) Heatmap visualizing the normalized mean expression of T cell exhaustion genes in CD8+ T-cells across samples. The mTGFβ-TRAP single-agent group was not included in analysis given the low infiltration of CD8+ T-cells in this sample. Combo represents a-PD-1 + mTGFβ-TRAP.

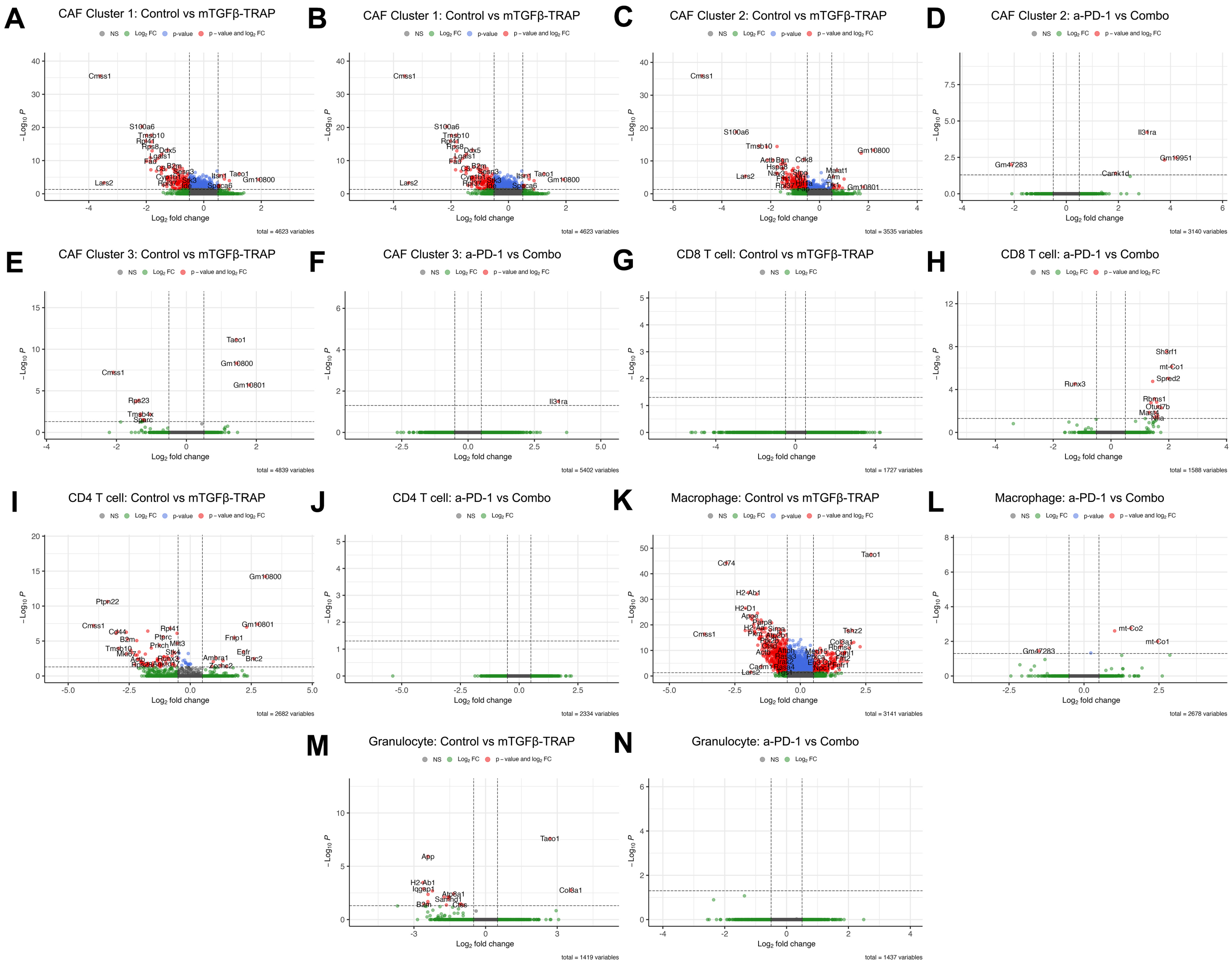

Supplemental Figure 13. Single-nuclear RNA-seq analysis of differentially expressed genes between different treatment groups. (A-N) Volcano plot of differentially expressed genes (DEG) between control and TGFβ-TRAP, a-PD1 and a-PD-1 + TGFβ-TRAP in CAF cluster 1 (A, B), CAF cluster 2 (C, D), CAF cluster 3 (E, F), CD8 T cells (G, H), CD4 T cells (I, J), macrophages (K, L), granulocytes (M, N). Significant DEGs meet the following criteria: adjusted p-value < 0.05 and $\log_2$(fold change) > 0.5 or <-0.5 using FindMarker function from Seurat with MAST method. Combo represents a-PD-1 + mTGFβ-TRAP.

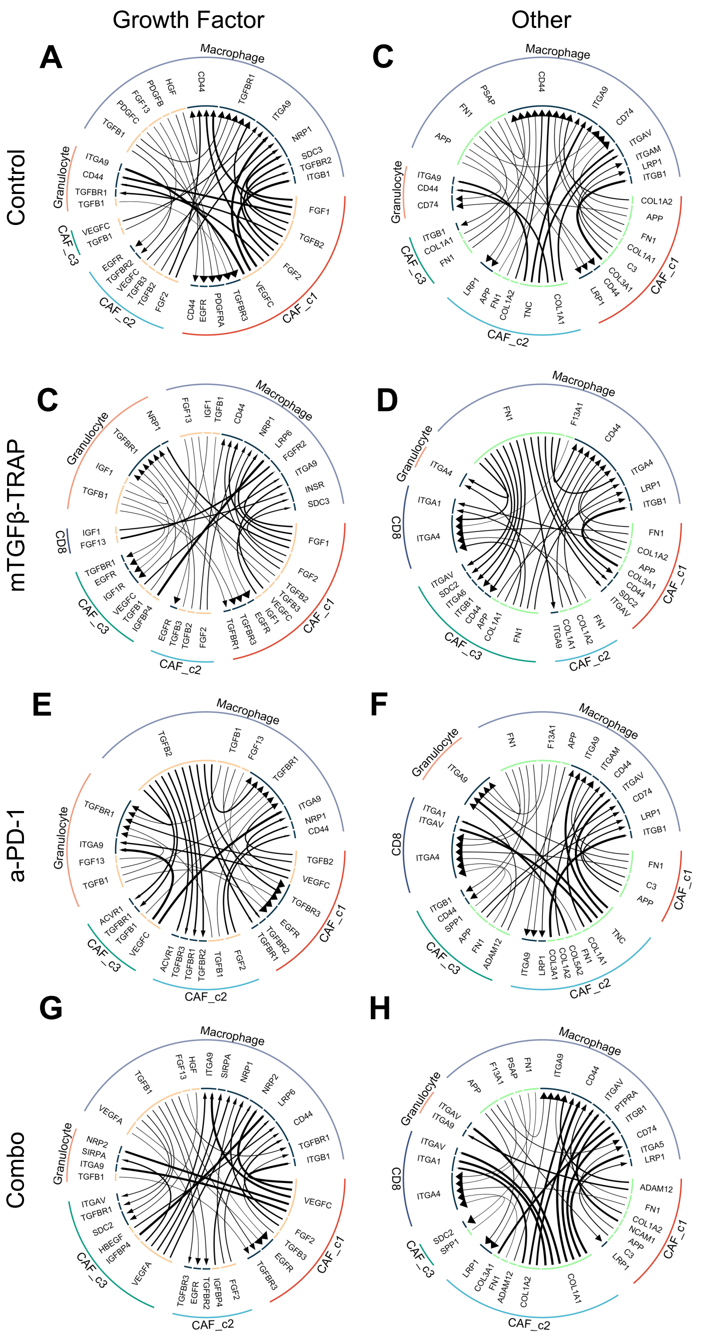

Supplemental Figure 14. Growth factor and other ligand-receptor analysis between CAFs and immune cells. (A-H) Chord diagram of ligand-receptor interactions between all three major clusters of CAFs, CD8 T-cells, macrophages, and granulocytes inferred by iTALK. Each row represents a treatment sample in the order of control **(A-B**), mTGFβ-TRAP **(C-D),** a-PD-1 **(E-F),** and combo (a-PD-1+mTGFβ-TRAP) **(G-H)** from top to bottom. Each column represents a category defined by iTALK, including growth factor and other.

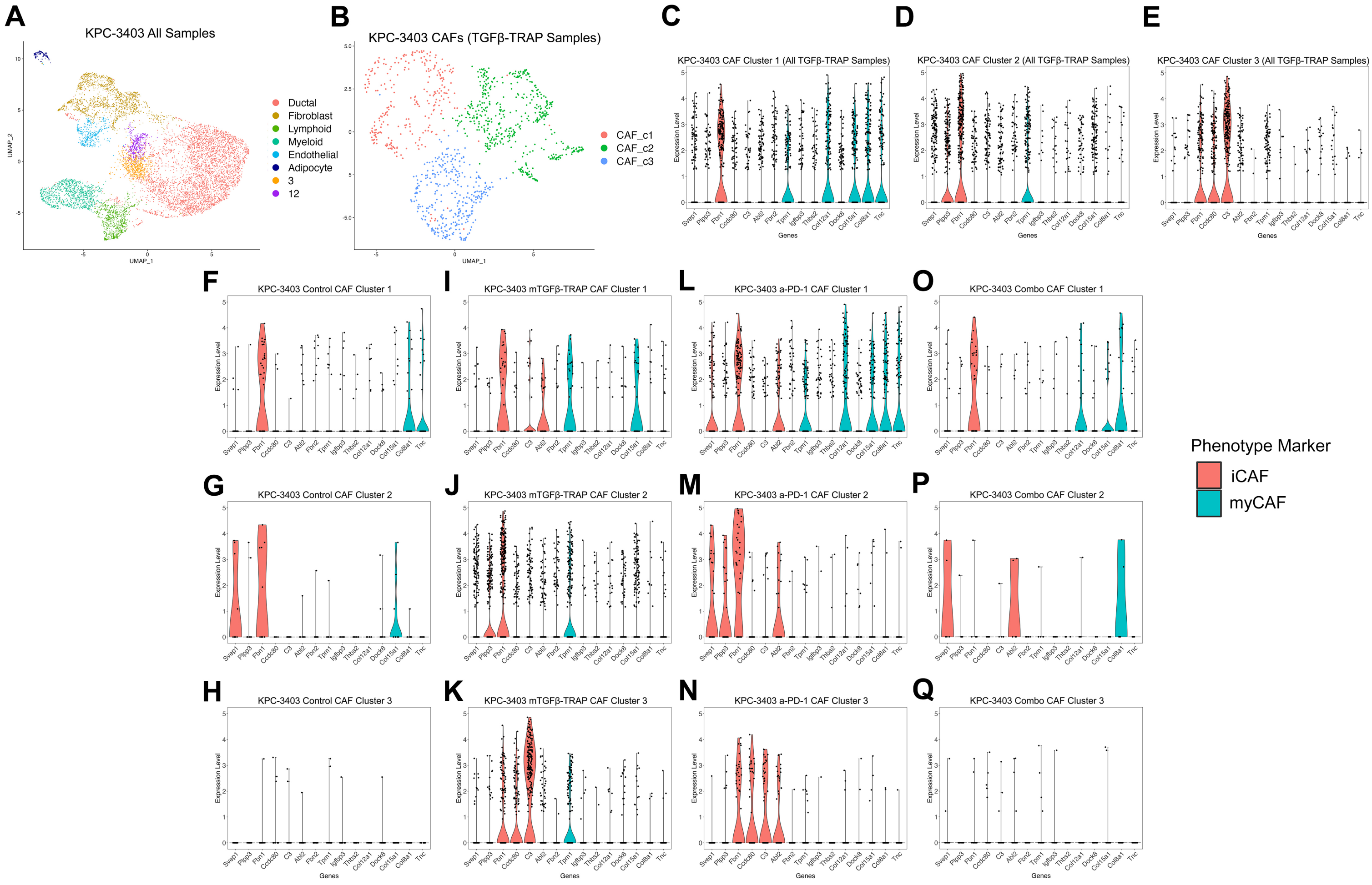

Supplemental Figure 15. CAF phenotype of the KPC-3403 tumor. (A) Uniform manifold approximation and projection (UMAP) embedding of single-nucleus profiles of representative cell types in the six KPC-3403 samples combined. (B) UMAP of the three major KPC-3403 CAF cluster across the four TGFβ-TRAP samples combined. (C-Q) KPC-3403 CAF phenotype gene panel with iCAF (red) and myCAF (blue) signature genes across the three major CAF clusters in all TGFβ-TRAP samples (C-E), control (F-H), TGFβ-TRAP treated (I-K), a-PD-1 treated (L-N), and a-PD-1 + TGFβ-TRAP treated samples (O-Q). Combo represents a-PD-1 + mTGFβ-TRAP.

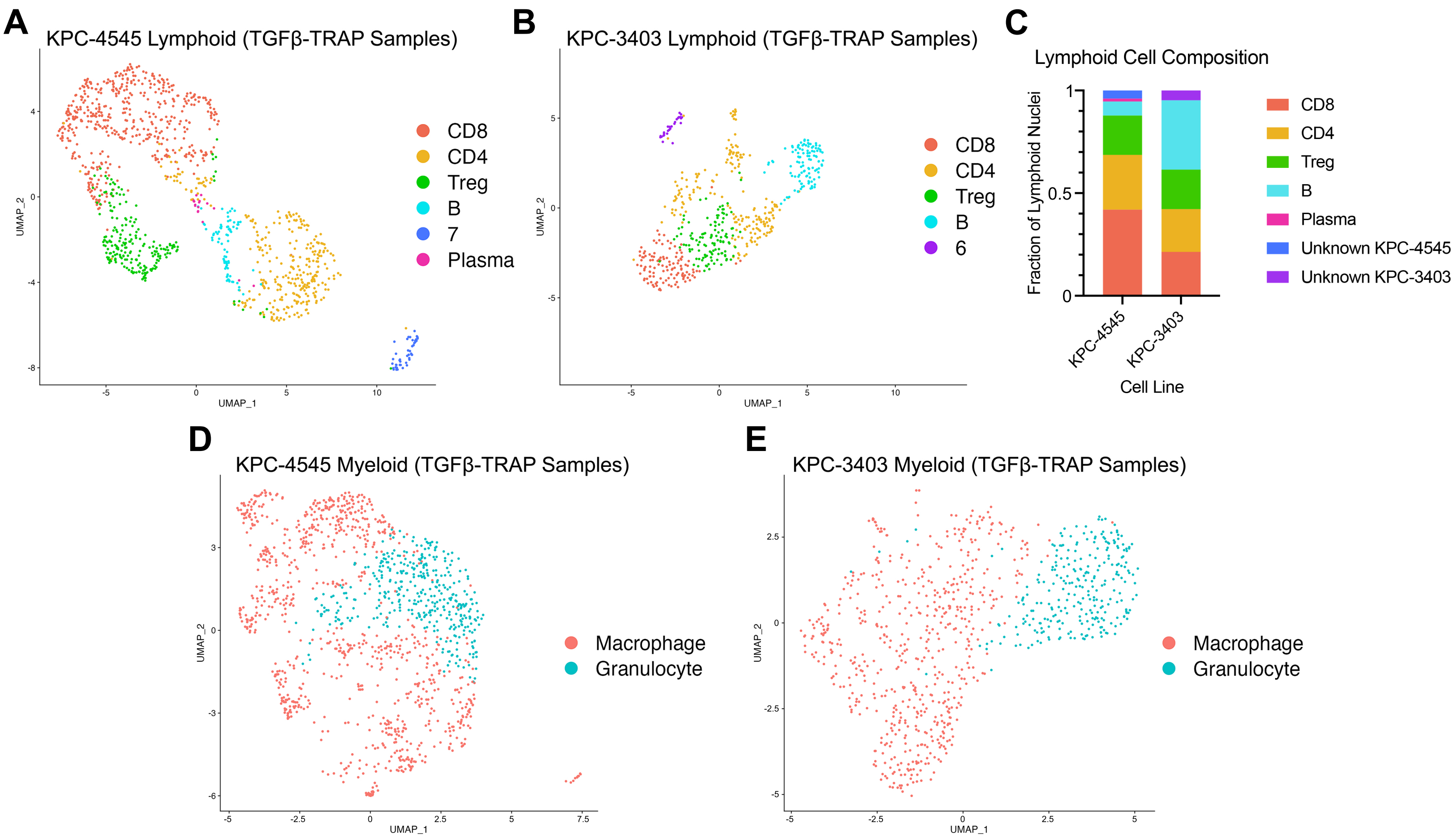

Supplemental Figure 16. Comparison of the immune landscape between KPC-4545 and KPC-3403 model. (A, B) Uniform manifold approximation and projection (UMAP) of annotated lymphoid subsets across the four TGFβ-TRAP samples combined in the KPC-4545 **(A)** and KPC-3403 models **(B)**. **(C)** Lymphoid cell type distribution across the four TGFβ-TRAP samples combined for each cell line. Fraction (y axis) of cell subsets across KPC-4545 (left) and KPC-3403 (right) model. **(D, E)** UMAP of annotated myeloid cells as macrophages and granulocytes across the four TGFβ-TRAP samples in the KPC-4545 **(D)** and KPC-3403 models **(E)**.

Supplementary Table 1. Cancer Associated Fibroblast Major and Subcluster Distribution

Major Cluster	Subcluster	**Control**	**TGFβ-TRAP**	**anti-PD-1**	**Combo**	**Total**	**Total**
CAF Cluster 1	s0	74	53	39	7	173	
	s1	7	85	37	20	149	
	s5	8	112	43	119	282	604
CAF Cluster 2	s2	125	58	50	33	266	
	s4	17	106	52	45	220	486
CAF Cluster 3	s3	53	136	19	5	213	
	s6	23	112	21	7	163	376
Total		307	662	261	236	1466	

Combo represents (anti-PD-1+ TGFβ-TRAP).

Supplementary Table 2. Top Differentially Expressed Genes of Each Cancer Associated Fibroblast Subcluster

p_val	avg_log2FC	pct.1	pct.2	p_val_adj	cluster	gene
1.31E-158	1.867	0.925	0.338	2.90E-154	s0	Sntg1
5.78E-153	1.625	0.984	0.54	1.28E-148	s0	Syne1
1.98E-139	2.039	0.733	0.189	4.38E-135	s0	Enpp2
2.22E-135	1.686	0.766	0.2	4.91E-131	s0	Wt1os
1.04E-122	1.897	0.596	0.121	2.31E-118	s0	Fgf1
8.36E-117	1.436	0.848	0.299	1.85E-112	s0	Dcn
1.58E-114	1.502	0.861	0.347	3.50E-110	s0	Mpp6
2.35E-114	1.510	0.793	0.262	5.20E-110	s0	C3
2.24E-113	1.402	0.872	0.325	4.97E-109	s0	Wt1
2.25E-112	1.563	0.698	0.19	4.98E-108	s0	Ildr2
4.39E-111	1.774	0.75	0.246	9.73E-107	s0	Bicd1
1.28E-100	1.210	0.952	0.499	2.84E-96	s0	Heg1
9.45E-99	1.899	0.541	0.13	2.09E-94	s0	Rapgef5
1.86E-97	1.424	0.735	0.239	4.13E-93	s0	Tmeff2
4.47E-97	1.463	0.823	0.335	9.90E-93	s0	Flrt2
3.42E-90	2.020	0.395	0.065	7.58E-86	s0	Mapk10
6.65E-89	1.081	0.865	0.352	1.47E-84	s0	Adgrd1
6.12E-85	1.168	0.852	0.398	1.36E-80	s0	Rbpms
1.66E-84	1.465	0.777	0.346	3.67E-80	s0	Ptpn13
1.24E-83	1.944	0.453	0.1	2.74E-79	s0	Igfbp5
2.22E-83	1.129	0.943	0.606	4.92E-79	s0	Exoc6b
2.95E-83	1.959	0.45	0.099	6.53E-79	s0	Gabrb1
4.27E-83	1.368	0.625	0.193	9.47E-79	s0	Arhgap28
4.64E-83	1.424	0.671	0.233	1.03E-78	s0	Ccdc80
1.92E-82	2.292	0.338	0.05	4.25E-78	s0	A530020G20Rik
2.12E-82	1.399	0.68	0.239	4.71E-78	s0	Celf4
1.96E-80	1.213	0.821	0.366	4.34E-76	s0	Naaladl2
1.72E-77	0.963	0.839	0.342	3.81E-73	s0	Zbtb7c
2.01E-76	1.594	0.554	0.171	4.46E-72	s0	Eml1
2.31E-76	1.276	0.629	0.205	5.12E-72	s0	Tmem108
1.33E-75	1.271	0.768	0.342	2.95E-71	s0	Sema5a
2.71E-75	1.584	0.439	0.099	6.01E-71	s0	Sez6l
4.59E-75	1.142	0.814	0.375	1.02E-70	s0	Frmd4b
8.44E-75	1.082	0.814	0.361	1.87E-70	s0	Tmtc2

8.38E-74	1.270	0.627	0.212	1.86E-69	s0	Arhgap29
2.64E-72	1.253	0.706	0.288	5.85E-68	s0	Gda
1.00E-71	1.141	0.863	0.488	2.22E-67	s0	Meis1
1.58E-71	2.142	0.291	0.041	3.51E-67	s0	Adamts19
2.62E-71	1.195	0.72	0.292	5.80E-67	s0	Cdon
1.15E-69	1.287	0.755	0.346	2.56E-65	s0	Add3
1.11E-68	1.011	0.793	0.354	2.46E-64	s0	Tgfbr3
7.20E-67	1.261	0.508	0.149	1.60E-62	s0	Nckap5
2.72E-66	1.219	0.764	0.338	6.03E-62	s0	Adamtsl1
9.03E-66	2.231	0.254	0.034	2.00E-61	s0	Vit
6.22E-65	1.103	0.753	0.35	1.38E-60	s0	Ust
5.00E-64	0.888	0.927	0.579	1.11E-59	s0	Airn
7.95E-64	1.457	0.57	0.21	1.76E-59	s0	Cxadr
2.00E-63	1.079	0.718	0.311	4.42E-59	s0	Fndc1
6.32E-63	1.665	0.388	0.094	1.40E-58	s0	Mrvi1
4.58E-61	1.664	0.38	0.094	1.01E-56	s0	Gabra3
8.99E-63	1.628	0.747	0.397	1.99E-58	s1	Sntg1
1.97E-54	1.446	0.818	0.591	4.35E-50	s1	Syne1
1.00E-33	0.952	0.886	0.839	2.22E-29	s1	Lrmda
1.10E-31	1.220	0.729	0.561	2.43E-27	s1	Heg1
2.35E-31	0.443	1	1	5.21E-27	s1	Malat1
2.61E-25	1.326	0.666	0.529	5.79E-21	s1	Il1r1
5.77E-24	1.642	0.451	0.267	1.28E-19	s1	Enpp2
2.19E-23	1.302	0.59	0.426	4.84E-19	s1	Adgrd1
3.16E-21	0.934	0.711	0.632	7.00E-17	s1	Nfia
7.67E-20	0.640	0.805	0.71	1.70E-15	s1	Tshz2
3.07E-18	1.268	0.561	0.443	6.80E-14	s1	Frmd4b
1.22E-17	0.566	0.895	0.815	2.69E-13	s1	Slit3
1.68E-16	1.133	0.545	0.42	3.73E-12	s1	Zbtb7c
2.79E-15	0.993	0.54	0.41	6.17E-11	s1	Flrt2
8.85E-15	1.584	0.428	0.306	1.96E-10	s1	Celf4
2.45E-14	1.152	0.522	0.416	5.42E-10	s1	Wt1
2.79E-14	1.282	0.516	0.435	6.17E-10	s1	Mpp6
8.59E-14	1.695	0.321	0.194	1.90E-09	s1	Fgf1
1.96E-13	1.291	0.484	0.392	4.35E-09	s1	Dcn
3.95E-13	0.636	0.726	0.722	8.75E-09	s1	Celf2
6.57E-13	1.019	0.666	0.674	1.46E-08	s1	Exoc6b
1.85E-12	0.789	0.648	0.585	4.09E-08	s1	Ror1
1.12E-10	1.168	0.489	0.418	2.47E-06	s1	Ust

1.38E-09	0.940	0.518	0.437	3.05E-05	s1	Tmtc2
1.82E-09	0.840	0.594	0.567	4.03E-05	s1	Gpr39
5.04E-09	1.191	0.466	0.419	0.00011162	s1	Add3
6.20E-09	1.009	0.516	0.482	0.00013742	s1	Rbpms
6.30E-09	1.279	0.365	0.273	0.00013962	s1	Tmem108
6.32E-09	0.465	0.785	0.83	0.00014001	s1	Zbtb20
6.34E-09	1.036	0.491	0.43	0.00014039	s1	Tgfbr3
1.13E-08	0.576	0.695	0.693	0.0002492	s1	Sox5
3.76E-08	1.299	0.372	0.3	0.00083204	s1	Wt1os
5.05E-08	0.514	0.767	0.824	0.0011176	s1	Rbms1
2.03E-07	1.260	0.417	0.369	0.00449084	s1	Cdon
3.84E-07	0.959	0.401	0.324	0.00851647	s1	Tmeff2
5.81E-07	0.526	0.655	0.704	0.01286956	s1	Xist
1.09E-06	0.270	0.132	0.266	0.0240669	s1	Pign
1.98E-06	0.881	0.455	0.415	0.04387812	s1	Adamtsl1
8.46E-141	3.034	0.586	0.091	1.87E-136	s2	Fbn2
4.07E-135	2.345	0.789	0.222	9.00E-131	s2	Cacna1c
2.37E-133	2.568	0.614	0.109	5.25E-129	s2	Kcnma1
7.99E-112	2.714	0.528	0.094	1.77E-107	s2	Csgalnact1
1.82E-109	2.102	0.764	0.237	4.04E-105	s2	Gpc6
1.35E-107	2.265	0.761	0.26	2.98E-103	s2	Spon1
9.79E-105	3.699	0.345	0.032	2.17E-100	s2	Lama1
5.48E-97	2.260	0.67	0.199	1.21E-92	s2	Adamts12
1.86E-95	2.615	0.645	0.195	4.11E-91	s2	Col12a1
7.55E-94	1.922	0.718	0.247	1.67E-89	s2	Ebf1
7.16E-91	2.666	0.64	0.194	1.59E-86	s2	Tnc
1.15E-86	1.803	0.647	0.187	2.55E-82	s2	Ptprm
1.84E-85	2.788	0.426	0.076	4.08E-81	s2	Epha3
8.28E-81	2.181	0.437	0.083	1.83E-76	s2	Cped1
2.75E-78	1.331	0.959	0.635	6.08E-74	s2	Pdzrn3
3.91E-77	2.211	0.569	0.165	8.66E-73	s2	Adam19
4.45E-75	2.463	0.396	0.074	9.86E-71	s2	Pdgfrb

2.81E-74	1.699	0.84	0.435	6.22E-70	s2	Dlc1
4.45E-74	2.366	0.388	0.072	9.85E-70	s2	Svep1
1.07E-73	2.609	0.363	0.062	2.36E-69	s2	Fap
1.40E-73	2.345	0.345	0.054	3.10E-69	s2	Prr16
9.51E-73	1.429	0.901	0.514	2.11E-68	s2	Col5a2
5.16E-70	2.422	0.579	0.201	1.14E-65	s2	Col8a1
5.79E-68	1.102	0.964	0.809	1.28E-63	s2	Rbms3
1.12E-67	2.698	0.279	0.037	2.49E-63	s2	Gabrb3
1.72E-66	2.450	0.426	0.099	3.81E-62	s2	Pcsk5
4.28E-66	3.265	0.251	0.029	9.47E-62	s2	Tspan18
2.29E-65	2.137	0.508	0.147	5.06E-61	s2	Col15a1
3.32E-65	1.473	0.868	0.52	7.34E-61	s2	Tcf4
3.51E-65	1.488	0.886	0.598	7.78E-61	s2	Cald1
3.99E-65	2.453	0.35	0.067	8.83E-61	s2	Unc5c
1.39E-64	1.993	0.678	0.28	3.08E-60	s2	Nav3
1.54E-64	1.847	0.553	0.18	3.40E-60	s2	Tln2
1.36E-63	1.916	0.467	0.125	3.02E-59	s2	Robo1
5.31E-63	2.882	0.363	0.079	1.18E-58	s2	Brinp3
8.55E-63	1.461	0.817	0.473	1.89E-58	s2	Fbn1
3.31E-59	1.466	0.825	0.467	7.33E-55	s2	Col5a1
8.81E-59	1.812	0.772	0.447	1.95E-54	s2	Kif26b
9.74E-59	1.666	0.579	0.208	2.16E-54	s2	Prrx1
4.53E-58	1.680	0.835	0.541	1.00E-53	s2	Col1a1
2.32E-57	2.846	0.277	0.045	5.13E-53	s2	Kcnq3
2.96E-57	2.013	0.487	0.155	6.56E-53	s2	Slc39a14
1.17E-55	1.545	0.599	0.239	2.58E-51	s2	Glis3
2.10E-54	2.564	0.322	0.068	4.66E-50	s2	Thbs2
1.67E-50	1.411	0.464	0.142	3.71E-46	s2	Mrc2
1.83E-50	0.905	0.942	0.801	4.06E-46	s2	Foxp1
7.49E-49	1.351	0.652	0.293	1.66E-44	s2	Setbp1
9.74E-49	2.868	0.284	0.059	2.16E-44	s2	Dkk2
1.21E-48	2.412	0.261	0.048	2.67E-44	s2	Postn
2.34E-48	1.436	0.741	0.397	5.18E-44	s2	Phldb2
6.96E-245	3.649	0.671	0.044	1.54E-240	s3	4930467D21Rik
8.44E-234	2.807	0.859	0.113	1.87E-229	s3	Cdh13
1.55E-230	2.895	0.759	0.077	3.43E-226	s3	Kirrel3
2.32E-229	2.624	0.928	0.149	5.14E-225	s3	Hmga2
6.45E-221	3.459	0.615	0.041	1.43E-216	s3	Gm49890

1.29E-218	2.575	0.979	0.213	2.86E-214	s3	Mecom
5.75E-193	2.753	0.74	0.095	1.27E-188	s3	Ltbp1
8.84E-189	3.746	0.477	0.023	1.96E-184	s3	Klhl1
7.18E-170	2.632	0.668	0.086	1.59E-165	s3	Prune2
3.07E-165	3.430	0.451	0.026	6.80E-161	s3	Sema3e
3.03E-164	2.507	0.615	0.071	6.71E-160	s3	Ank3
2.94E-160	2.307	0.743	0.128	6.52E-156	s3	Adgrl3
1.35E-154	3.952	0.401	0.02	2.98E-150	s3	Cdkn2a
6.09E-150	2.277	0.753	0.142	1.35E-145	s3	Kcnq5
2.12E-148	2.316	0.703	0.12	4.69E-144	s3	Ank
7.54E-142	2.884	0.501	0.051	1.67E-137	s3	Adgrg6
5.44E-139	2.017	0.735	0.144	1.20E-134	s3	Nav2
6.18E-138	2.686	0.485	0.049	1.37E-133	s3	Fnbp1l
2.46E-136	2.468	0.541	0.067	5.45E-132	s3	Edil3
5.65E-134	2.350	0.57	0.078	1.25E-129	s3	Tiam2
1.51E-133	3.024	0.448	0.041	3.34E-129	s3	Cpe
4.79E-133	2.060	0.822	0.207	1.06E-128	s3	Epn2
6.31E-130	3.389	0.387	0.027	1.40E-125	s3	Cdh1
2.29E-129	2.773	0.48	0.054	5.07E-125	s3	Arhgef28
1.77E-127	4.288	0.31	0.012	3.92E-123	s3	Itga3
9.73E-126	2.559	0.541	0.078	2.15E-121	s3	Anxa3
5.97E-119	3.155	0.318	0.017	1.32E-114	s3	Hmgcll1
1.12E-117	3.252	0.355	0.026	2.47E-113	s3	Onecut2
6.31E-115	1.734	0.942	0.376	1.40E-110	s3	Morrbid
2.43E-110	1.729	0.886	0.309	5.38E-106	s3	Cdk6
5.75E-110	2.593	0.385	0.037	1.27E-105	s3	Ccnd1
4.88E-109	1.890	0.687	0.154	1.08E-104	s3	Osbpl3
2.21E-102	2.165	0.509	0.084	4.90E-98	s3	Errfi1
1.29E-101	1.268	0.756	0.195	2.86E-97	s3	Alcam
4.53E-101	1.764	0.814	0.27	1.00E-96	s3	Nbea
2.63E-100	2.818	0.321	0.026	5.82E-96	s3	Lama5
1.35E-99	1.788	0.7	0.183	2.99E-95	s3	Slc4a4
5.27E-99	2.697	0.316	0.025	1.17E-94	s3	Eva1c
5.27E-99	2.802	0.329	0.029	1.17E-94	s3	Slc12a2
2.69E-97	1.874	0.647	0.155	5.95E-93	s3	Oxr1
3.00E-96	2.021	0.597	0.133	6.64E-92	s3	Pkp4
4.06E-96	1.899	0.512	0.09	9.00E-92	s3	Cadm1
7.24E-96	3.209	0.276	0.017	1.60E-91	s3	Pax3
7.80E-95	1.911	0.496	0.085	1.73E-90	s3	Myo1b

3.09E-94	2.605	0.435	0.066	6.85E-90	s3	Chka
3.54E-94	2.935	0.273	0.017	7.84E-90	s3	Igf2bp1
5.35E-94	2.424	0.509	0.094	1.19E-89	s3	Spp1
5.31E-93	2.439	0.411	0.058	1.18E-88	s3	Itga6
3.79E-91	1.331	0.788	0.227	8.41E-87	s3	Runx2
3.16E-90	1.689	0.735	0.22	7.00E-86	s3	Msn
3.87E-42	1.985	0.559	0.264	8.57E-38	s4	Cacna1c
2.18E-39	2.139	0.556	0.277	4.82E-35	s4	Ebf1
7.16E-16	2.064	0.312	0.16	1.59E-11	s4	Kcnma1
1.83E-15	1.526	0.449	0.29	4.06E-11	s4	Gpc6
3.40E-15	1.399	0.466	0.31	7.52E-11	s4	Spon1
4.47E-14	1.672	0.373	0.234	9.91E-10	s4	Ptprm
1.72E-13	0.359	0.997	1	3.80E-09	s4	Malat1
4.49E-13	1.475	0.611	0.532	9.95E-09	s4	Gm10800
3.86E-12	0.441	0.932	0.969	8.54E-08	s4	Gm26917
9.68E-11	0.598	0.751	0.738	2.14E-06	s4	Taco1
1.24E-10	0.769	0.8	0.836	2.75E-06	s4	Rbms3
3.42E-09	0.735	0.77	0.828	7.57E-05	s4	Foxp1
7.31E-08	1.472	0.345	0.254	0.00161897	s4	Adamts12
2.20E-07	1.339	0.353	0.27	0.00487539	s4	Arhgap15
8.88E-07	1.531	0.329	0.246	0.01966267	s4	Tnc
1.11E-06	0.906	0.425	0.358	0.02457471	s4	Dock2
1.98E-06	1.139	0.504	0.491	0.04388941	s4	Dlc1
2.23E-145	2.983	0.836	0.241	4.94E-141	s5	Thsd4
2.50E-123	4.128	0.405	0.036	5.55E-119	s5	Muc16
2.08E-105	3.420	0.493	0.082	4.60E-101	s5	Gpm6a
2.14E-94	3.819	0.29	0.021	4.73E-90	s5	Pcnx2
3.54E-81	3.715	0.361	0.052	7.84E-77	s5	Kcnab1
9.28E-80	1.782	0.886	0.464	2.06E-75	s5	Plxna4
1.76E-78	2.444	0.49	0.11	3.90E-74	s5	Ppp2r2b
7.95E-78	3.224	0.531	0.141	1.76E-73	s5	Shroom3
8.49E-73	1.656	0.874	0.489	1.88E-68	s5	Kalrn
1.72E-71	1.694	0.897	0.605	3.81E-67	s5	Efna5
1.10E-70	3.069	0.378	0.068	2.45E-66	s5	Pdzd2
6.41E-70	1.866	0.739	0.309	1.42E-65	s5	Plce1

6.32E-69	1.166	0.979	0.831	1.40E-64	s5	Mast4
1.15E-68	3.329	0.293	0.038	2.56E-64	s5	Upk1b
2.43E-67	3.561	0.252	0.027	5.37E-63	s5	Cdh3
9.05E-59	1.292	0.906	0.712	2.00E-54	s5	Zfpm2
3.30E-57	1.406	0.806	0.389	7.31E-53	s5	Zbtb7c
1.06E-56	2.141	0.487	0.144	2.36E-52	s5	Pkhd1l1
3.95E-56	3.493	0.378	0.092	8.76E-52	s5	Rbfox1
5.70E-56	1.986	0.587	0.22	1.26E-51	s5	Sox6
5.97E-53	2.583	0.408	0.108	1.32E-48	s5	Myo5b
5.66E-49	2.098	0.472	0.159	1.25E-44	s5	Nos1ap
2.04E-43	1.024	0.903	0.567	4.51E-39	s5	Pcdh7
2.70E-41	1.137	0.818	0.491	5.97E-37	s5	Bicc1
1.18E-40	1.252	0.76	0.407	2.61E-36	s5	Tmtc2
1.11E-38	1.766	0.367	0.108	2.46E-34	s5	Col4a6
1.33E-38	2.120	0.39	0.128	2.94E-34	s5	Lrrc1
5.25E-38	2.710	0.334	0.098	1.16E-33	s5	Il18r1
6.71E-38	1.496	0.589	0.283	1.49E-33	s5	Cfh
1.24E-37	2.262	0.466	0.202	2.74E-33	s5	Syne2
9.92E-37	1.613	0.501	0.206	2.20E-32	s5	Klhl29
1.71E-36	2.060	0.29	0.074	3.80E-32	s5	Lvrn
1.43E-35	1.922	0.587	0.316	3.18E-31	s5	Lrba
9.44E-35	1.220	0.713	0.406	2.09E-30	s5	Spsb1
1.20E-34	1.627	0.384	0.126	2.65E-30	s5	Tmtc1
2.82E-33	0.706	0.9	0.544	6.25E-29	s5	Heg1
1.00E-32	1.748	0.519	0.235	2.22E-28	s5	Col24a1
6.53E-31	1.724	0.493	0.235	1.45E-26	s5	Crim1
9.03E-31	1.845	0.431	0.184	2.00E-26	s5	Epb41l4a
1.51E-30	1.440	0.563	0.298	3.34E-26	s5	Myo1d
4.13E-30	1.954	0.302	0.094	9.15E-26	s5	Tmem132d
5.93E-30	1.541	0.343	0.115	1.31E-25	s5	C1qtnf7
9.33E-30	1.340	0.551	0.279	2.07E-25	s5	Ptprd
1.12E-29	1.483	0.434	0.174	2.48E-25	s5	Bmper
4.32E-29	1.371	0.61	0.345	9.57E-25	s5	Adam12
9.61E-29	1.915	0.387	0.161	2.13E-24	s5	Ezr
1.18E-28	1.239	0.657	0.417	2.61E-24	s5	Ppfibp1
2.68E-28	2.016	0.337	0.122	5.95E-24	s5	Chn2
2.80E-28	0.773	0.968	0.809	6.20E-24	s5	Slit3
3.38E-27	1.123	0.563	0.288	7.49E-23	s5	Pde3a
1.62E-79	2.178	0.741	0.262	3.59E-75	s6	Mecom

9.65E-67	2.223	0.625	0.207	2.14E-62	s6	Hmga2
3.48E-43	1.242	0.972	0.964	7.70E-39	s6	Gm26917
8.45E-41	2.101	0.489	0.191	1.87E-36	s6	Kcnq5
2.63E-38	1.986	0.476	0.181	5.84E-34	s6	Cdh13
1.32E-37	1.604	0.703	0.42	2.92E-33	s6	Morrbid
5.03E-32	2.105	0.451	0.195	1.11E-27	s6	Nav2
4.27E-25	1.561	0.577	0.363	9.46E-21	s6	Cdk6
8.48E-23	2.219	0.347	0.147	1.88E-18	s6	Kirrel3
1.39E-22	1.508	0.681	0.525	3.09E-18	s6	Gm10800
2.46E-22	2.126	0.35	0.154	5.44E-18	s6	Inpp4b
3.27E-22	1.276	0.808	0.731	7.25E-18	s6	Taco1
3.82E-21	1.895	0.334	0.143	8.46E-17	s6	Prune2
8.96E-21	1.099	0.748	0.625	1.98E-16	s6	Ahnak
1.36E-20	1.506	0.637	0.455	3.00E-16	s6	Cdk8
1.69E-20	1.611	0.533	0.346	3.75E-16	s6	Dock2
6.35E-18	1.559	0.36	0.179	1.41E-13	s6	Ank
5.92E-17	0.754	0.779	0.631	1.31E-12	s6	Runx1
1.04E-16	1.492	0.438	0.261	2.30E-12	s6	Arhgap15
9.18E-16	1.167	0.53	0.372	2.03E-11	s6	Elmo1
5.90E-15	1.649	0.366	0.205	1.31E-10	s6	Bcl2
1.56E-14	0.751	0.719	0.6	3.45E-10	s6	Msi2
2.64E-14	1.220	0.549	0.413	5.85E-10	s6	Gse1
6.73E-14	1.417	0.319	0.164	1.49E-09	s6	Ltbp1
6.80E-14	1.384	0.379	0.223	1.51E-09	s6	Plcl2
7.49E-14	1.115	0.524	0.374	1.66E-09	s6	Rad51b
1.14E-13	1.792	0.262	0.123	2.53E-09	s6	Myo1f
3.35E-13	1.205	0.502	0.362	7.42E-09	s6	Mllt3
3.61E-13	1.203	0.536	0.428	7.99E-09	s6	Fam49b
3.81E-13	1.099	0.558	0.423	8.43E-09	s6	Fmnl2
6.47E-13	0.909	0.574	0.413	1.43E-08	s6	Maml3
1.10E-12	1.834	0.265	0.129	2.44E-08	s6	Ank3
1.15E-12	0.919	0.65	0.542	2.56E-08	s6	Peak1
1.24E-12	1.422	0.413	0.26	2.76E-08	s6	Gm10801
5.11E-12	0.926	0.644	0.546	1.13E-07	s6	Cmip
6.23E-12	1.693	0.328	0.196	1.38E-07	s6	Tmem131l
8.29E-12	1.510	0.338	0.195	1.84E-07	s6	Adgrl3
9.50E-12	1.761	0.262	0.134	2.10E-07	s6	Il34
1.28E-11	0.809	0.593	0.435	2.84E-07	s6	Pmepa1
1.42E-11	0.828	0.662	0.603	3.14E-07	s6	Ankrd11

1.98E-11	1.314	0.426	0.287	4.38E-07	s6	Runx2
5.11E-11	0.935	0.574	0.459	1.13E-06	s6	Cdk14
7.12E-11	1.727	0.3	0.176	1.58E-06	s6	Chd7
8.81E-11	1.231	0.511	0.38	1.95E-06	s6	Camk1d
3.38E-10	1.095	0.634	0.548	7.49E-06	s6	Tenm3
3.61E-10	1.428	0.265	0.141	8.00E-06	s6	Nrg1
5.55E-10	1.304	0.331	0.202	1.23E-05	s6	Ptprc
7.99E-10	0.651	0.801	0.755	1.77E-05	s6	Neat1
8.88E-10	1.462	0.268	0.15	1.97E-05	s6	Inpp5d
1.35E-09	1.566	0.252	0.141	2.99E-05	s6	Tpd52

Supplementary Table 3. Top 30 Growth Factor Interactions in Control Sample

Top 30 Growth Factor Interactions in Control					
**ligand**	**receptor**	**cell_from**	**cell_to**	**Present in Control Top 30**	**Present in TGFβ-TRAP Top 30**
FGF1	CD44	CAF_c1	Macrophage	Yes	Yes
FGF1	NRP1	CAF_c1	Macrophage	Yes	Yes
FGF1	CD44	CAF_c1	Granulocyte	Yes	No
FGF13	EGFR	Macrophage	CAF_c1	Yes	Yes
FGF13	EGFR	Macrophage	CAF_c2	Yes	Yes
FGF2	CD44	CAF_c1	Macrophage	Yes	Yes
FGF2	CD44	CAF_c2	Macrophage	Yes	Yes
FGF2	NRP1	CAF_c1	Macrophage	Yes	Yes
FGF2	NRP1	CAF_c2	Macrophage	Yes	Yes
FGF2	CD44	CAF_c1	Granulocyte	Yes	No
FGF2	SDC3	CAF_c1	Macrophage	Yes	Yes
HGF	CD44	Macrophage	Macrophage	Yes	No
HGF	CD44	Macrophage	CAF_c1	Yes	No
PDGFB	PDGFRA	Macrophage	CAF_c1	Yes	No
PDGFC	PDGFRA	Macrophage	CAF_c1	Yes	No
TGFB1	TGFBR3	Macrophage	CAF_c1	Yes	Yes
TGFB1	TGFBR1	Macrophage	Macrophage	Yes	No
TGFB1	TGFBR1	CAF_c3	Macrophage	Yes	No
TGFB1	TGFBR3	Granulocyte	CAF_c1	Yes	Yes
TGFB1	TGFBR2	Macrophage	CAF_c2	Yes	No
TGFB2	TGFBR1	CAF_c1	Macrophage	Yes	No
TGFB2	TGFBR1	CAF_c2	Macrophage	Yes	No
TGFB2	TGFBR1	CAF_c1	Granulocyte	Yes	Yes
TGFB2	TGFBR2	CAF_c1	Macrophage	Yes	No
TGFB3	TGFBR1	CAF_c2	Macrophage	Yes	No
VEGFC	ITGA9	CAF_c1	Macrophage	Yes	Yes
VEGFC	ITGA9	CAF_c3	Macrophage	Yes	Yes
VEGFC	ITGA9	CAF_c1	Granulocyte	Yes	No
VEGFC	ITGA9	CAF_c2	Macrophage	Yes	No
VEGFC	ITGB1	CAF_c1	Macrophage	Yes	No

Top thirty growth factor interactions as inferred by iTALK between all three clusters of cancer associated fibroblasts (CAF_c1, CAF_c2, CAF_c3), CD8 T cells, macrophage, and granulocytes in the Control sample. Interactions between cancer associated fibroblasts clusters were excluded. Last column represents whether interaction was in the top thirty growth factor interactions of the TGFβ-TRAP sample.

Supplementary Table 4. Top 30 Growth Factors in TGFβ-TRAP Sample

Top 30 Growth Factor Interactions in TGFB-TRAP					
**ligand**	**receptor**	**cell_from**	**cell_to**	**Present in TGFβ-TRAP Top 30**	**Present in Control Top 30**
FGF1	CD44	CAF_c1	Macrophage	Yes	Yes
FGF1	NRP1	CAF_c1	Macrophage	Yes	Yes
FGF1	NRP1	CAF_c1	Granulocyte	Yes	No
FGF1	FGFR2	CAF_c1	Macrophage	Yes	No
FGF13	EGFR	Macrophage	CAF_c1	Yes	Yes
FGF13	EGFR	Macrophage	CAF_c3	Yes	No
FGF13	EGFR	Macrophage	CAF_c2	Yes	Yes
FGF13	FGFR2	CD8	Macrophage	Yes	No
FGF2	CD44	CAF_c1	Macrophage	Yes	Yes
FGF2	CD44	CAF_c2	Macrophage	Yes	Yes
FGF2	NRP1	CAF_c1	Macrophage	Yes	Yes
FGF2	NRP1	CAF_c2	Macrophage	Yes	Yes
FGF2	SDC3	CAF_c1	Macrophage	Yes	Yes
IGF1	IGF1R	Macrophage	CAF_c3	Yes	No
IGF1	IGF1R	Granulocyte	CAF_c3	Yes	No
IGF1	INSR	CAF_c1	Macrophage	Yes	No
IGF1	INSR	CD8	Macrophage	Yes	No
IGFBP4	LRP6	CAF_c3	Macrophage	Yes	No
TGFB1	TGFBR3	Granulocyte	CAF_c1	Yes	Yes
TGFB1	TGFBR1	CAF_c3	Granulocyte	Yes	No
TGFB1	TGFBR1	Granulocyte	CAF_c3	Yes	No
TGFB1	TGFBR3	Macrophage	CAF_c1	Yes	Yes
TGFB1	TGFBR1	Granulocyte	Granulocyte	Yes	No
TGFB1	TGFBR1	Granulocyte	CAF_c1	Yes	No
TGFB2	TGFBR1	CAF_c1	Granulocyte	Yes	Yes
TGFB2	TGFBR1	CAF_c2	Granulocyte	Yes	No
TGFB3	TGFBR1	CAF_c1	Granulocyte	Yes	No
TGFB3	TGFBR1	CAF_c2	Granulocyte	Yes	No
VEGFC	ITGA9	CAF_c1	Macrophage	Yes	Yes
VEGFC	ITGA9	CAF_c3	Macrophage	Yes	Yes

Top thirty growth factor interactions as inferred by iTALK between all three clusters of cancer associated fibroblasts (CAF_c1, CAF_c2, CAF_c3), CD8 T cells, macrophage, and granulocytes in the TGFβ-TRAP sample. Interactions between cancer associated fibroblasts clusters were excluded. Last column represents whether interaction was in the top thirty growth factor interactions of the Control sample.

Supplementary Table 5. Top 30 Cytokine Interactions in Control Sample

Top 30 Cytokine Interactions in Control					
**ligand**	**receptor**	**cell_from**	**cell_to**	**Present in Control Top 30**	**Present in TGFβ-TRAP Top 30**
CCL13	CCR5	CAF_c1	Macrophage	Yes	No
CCL13	CCR5	CAF_c1	Granulocyte	Yes	No
CCL4	CCR5	CD8	Macrophage	Yes	No
CCL5	CCR5	Granulocyte	Macrophage	Yes	No
CCL5	SDC4	Granulocyte	CAF_c1	Yes	No
CCL5	CCR5	Granulocyte	Granulocyte	Yes	No
CCL8	CCR5	Macrophage	Macrophage	Yes	No
CXCL10	SDC4	Granulocyte	CAF_c1	Yes	No
IL15	IL2RB	Macrophage	CD8	Yes	Yes
IL16	CCR5	CAF_c1	Macrophage	Yes	Yes
IL16	CCR5	CAF_c1	Granulocyte	Yes	Yes
IL16	CCR5	CAF_c1	CD8	Yes	Yes
IL16	CCR5	Macrophage	Macrophage	Yes	Yes
IL16	CCR5	CAF_c2	Macrophage	Yes	Yes
IL16	CCR5	Macrophage	Granulocyte	Yes	Yes
IL18	IL1RAPL1	Macrophage	CAF_c1	Yes	No
IL18	IL1RAPL1	Granulocyte	CAF_c1	Yes	No
IL18	IL1RAPL1	Macrophage	CAF_c3	Yes	No
IL1A	IL1R1	Granulocyte	CAF_c1	Yes	Yes
IL1A	IL1R1	Macrophage	CAF_c1	Yes	Yes
IL1B	IL1R1	Granulocyte	CAF_c1	Yes	Yes
IL1B	IL1R1	Macrophage	CAF_c1	Yes	Yes
IL1B	IL1R1	Granulocyte	CAF_c2	Yes	Yes
IL1B	IL1R1	Macrophage	CAF_c2	Yes	Yes
IL1B	IL1R1	Granulocyte	CAF_c3	Yes	Yes
IL1B	IL1R1	Macrophage	CAF_c3	Yes	Yes
IL1RN	IL1R1	Granulocyte	CAF_c1	Yes	No
IL34	CSF1R	CAF_c3	Macrophage	Yes	Yes
IL34	CSF1R	CAF_c3	Granulocyte	Yes	Yes
IL34	CSF1R	CAF_c1	Macrophage	Yes	No

Top thirty cytokine interactions as inferred by iTALK between all three clusters of cancer associated fibroblasts (CAF_c1, CAF_c2, CAF_c3), CD8 T cells, macrophage, and granulocytes in the Control sample. Interactions between cancer associated fibroblasts clusters were excluded. Last column represents whether interaction was in the top thirty cytokine interactions of the TGFβ-TRAP sample.

Supplementary Table 6. Top 30 Cytokine Interactions in TGFβ-TRAP Sample

Top 30 Cytokines Interactions in TGFB-TRAP					
**ligand**	**receptor**	**cell_from**	**cell_to**	**Present in TGFβ-TRAP Top 30**	**Present in Control Top 30**
CCL5	CCR5	CAF_c3	Macrophage	Yes	No
CXCL12	ITGB1	Granulocyte	CAF_c3	Yes	No
IL15	IL2RB	CAF_c3	CD8	Yes	No
IL15	IL2RB	Macrophage	CD8	Yes	Yes
IL15	IL15RA	CAF_c3	Macrophage	Yes	No
IL16	CCR5	CAF_c1	Macrophage	Yes	Yes
IL16	CCR5	CAF_c1	Granulocyte	Yes	Yes
IL16	CCR5	CAF_c1	CD8	Yes	Yes
IL16	KCND2	CAF_c1	CD8	Yes	No
IL16	CCR5	Macrophage	Macrophage	Yes	Yes
IL16	CCR5	CAF_c3	Macrophage	Yes	No
IL16	CCR5	Granulocyte	Macrophage	Yes	No
IL16	CCR5	Macrophage	CAF_c3	Yes	No
IL16	CCR5	CAF_c2	Macrophage	Yes	Yes
IL16	CCR5	Macrophage	Granulocyte	Yes	Yes
IL16	KCND2	Macrophage	CAF_c1	Yes	No
IL1A	IL1R1	Granulocyte	CAF_c1	Yes	Yes
IL1A	IL1R1	Macrophage	CAF_c1	Yes	Yes
IL1A	IL1R1	Granulocyte	CAF_c2	Yes	No
IL1A	IL1R1	Granulocyte	CAF_c3	Yes	No
IL1B	IL1R1	Macrophage	CAF_c1	Yes	Yes
IL1B	IL1R1	Granulocyte	CAF_c1	Yes	Yes
IL1B	IL1R1	Macrophage	CAF_c2	Yes	Yes
IL1B	IL1R1	Macrophage	CAF_c3	Yes	Yes
IL1B	IL1R1	Granulocyte	CAF_c2	Yes	Yes
IL1B	IL1R1	Granulocyte	CAF_c3	Yes	Yes
IL1RN	IL1R1	Macrophage	CAF_c1	Yes	No
IL34	CSF1R	CAF_c3	Macrophage	Yes	Yes
IL34	CSF1R	CAF_c3	Granulocyte	Yes	Yes
IL34	CSF1R	CAF_c2	Macrophage	Yes	No

Top thirty cytokine interactions as inferred by iTALK between all three clusters of cancer associated fibroblasts (CAF_c1, CAF_c2, CAF_c3), CD8 T cells, macrophage, and granulocytes in the TGFβ-TRAP sample. Interactions between cancer associated fibroblasts clusters were excluded. Last column represents whether interaction was in the top thirty cytokine interactions of the Control sample.

Supplementary Table 7. Top 30 Checkpoint Interactions in Control Sample

Top 30 Checkpoint Interactions in Control					
**ligand**	**receptor**	**cell_from**	**cell_to**	**Present in Control Top 30**	**Present in TGFβ-TRAP Top 30**
BTLA	CD247	CAF_c3	CD8	Yes	Yes
BTLA	CD247	CD8	CD8	Yes	No
BTLA	CD247	CAF_c1	CD8	Yes	No
BTLA	CD247	Granulocyte	CD8	Yes	Yes
CD274	PDCD1	Granulocyte	CD8	Yes	No
CD274	PDCD1	Macrophage	CD8	Yes	No
CD274	PDCD1	CAF_c3	CD8	Yes	No
CD274	PDCD1	Granulocyte	CAF_c3	Yes	No
CD40LG	ITGAM	CD8	Macrophage	Yes	No
CD40LG	TRAF3	CD8	Macrophage	Yes	No
CD40LG	ITGB2	CD8	Macrophage	Yes	No
CD80	CD28	Macrophage	CD8	Yes	No
CD80	CTLA4	Macrophage	CD8	Yes	No
CD86	CD28	Granulocyte	CD8	Yes	No
CD86	CD28	Macrophage	CD8	Yes	No
CD86	CTLA4	Granulocyte	CD8	Yes	No
CD86	CTLA4	Macrophage	CD8	Yes	No
CD86	CTLA4	Granulocyte	CAF_c3	Yes	No
CD86	CTLA4	Macrophage	CAF_c3	Yes	No
CD86	CD28	CAF_c3	CD8	Yes	No
CD86	CD28	Granulocyte	CAF_c3	Yes	No
CD86	CTLA4	CAF_c3	CD8	Yes	No
CD86	CD28	Macrophage	CAF_c3	Yes	No
LGALS9	HAVCR2	Macrophage	Macrophage	Yes	Yes
LGALS9	HAVCR2	Macrophage	Granulocyte	Yes	Yes
LGALS9	HAVCR2	CAF_c1	Macrophage	Yes	Yes
LGALS9	HAVCR2	CAF_c2	Macrophage	Yes	Yes
LGALS9	HAVCR2	CAF_c3	Macrophage	Yes	Yes
LGALS9	HAVCR2	CAF_c1	Granulocyte	Yes	Yes
PDCD1LG2	PDCD1	Granulocyte	CD8	Yes	No

Top thirty checkpoint interactions as inferred by iTALK between all three clusters of cancer associated fibroblasts (CAF_c1, CAF_c2, CAF_c3), CD8 T cells, macrophage, and granulocytes in the Control sample. Interactions between cancer associated fibroblasts clusters were excluded. Last column represents whether interaction was in the top thirty checkpoint interactions of the TGFβ-TRAP sample.

Supplementary Table 8. Top 30 Checkpoint Interactions in TGFβ-TRAP Sample

Top 30 Checkpoint Interactions in TGFB-TRAP					
**ligand**	**receptor**	**cell_from**	**cell_to**	**Present in TGFβ-TRAP Top 30**	**Present in Control Top 30**
BTLA	CD247	CAF_c3	CD8	Yes	Yes
BTLA	CD247	Granulocyte	CD8	Yes	Yes
BTLA	CD247	CAF_c2	CD8	Yes	No
BTLA	CD247	Macrophage	CD8	Yes	No
BTLA	CD247	CAF_c3	Macrophage	Yes	No
BTLA	VTCN1	Granulocyte	CAF_c3	Yes	No
BTLA	CD247	CAF_c3	Granulocyte	Yes	No
BTLA	TNFRSF14	CAF_c3	Granulocyte	Yes	No
CD86	CTLA4	Macrophage	Macrophage	Yes	No
LGALS9	HAVCR2	CAF_c3	CD8	Yes	No
LGALS9	HAVCR2	CAF_c2	CD8	Yes	No
LGALS9	HAVCR2	CAF_c1	CD8	Yes	No
LGALS9	HAVCR2	Macrophage	CD8	Yes	No
LGALS9	HAVCR2	CAF_c3	Macrophage	Yes	Yes
LGALS9	HAVCR2	CAF_c2	Macrophage	Yes	Yes
LGALS9	HAVCR2	Granulocyte	CD8	Yes	No
LGALS9	HAVCR2	CAF_c1	Macrophage	Yes	Yes
LGALS9	HAVCR2	Macrophage	Macrophage	Yes	Yes
LGALS9	HAVCR2	Macrophage	CAF_c3	Yes	No
LGALS9	HAVCR2	Granulocyte	Macrophage	Yes	No
LGALS9	HAVCR2	CAF_c3	Granulocyte	Yes	No
LGALS9	HAVCR2	Macrophage	CAF_c1	Yes	No
LGALS9	HAVCR2	Macrophage	CAF_c2	Yes	No
LGALS9	HAVCR2	Granulocyte	CAF_c3	Yes	No
LGALS9	HAVCR2	CAF_c2	Granulocyte	Yes	No
LGALS9	HAVCR2	CAF_c1	Granulocyte	Yes	Yes
LGALS9	HAVCR2	Macrophage	Granulocyte	Yes	No
LGALS9	HAVCR2	Granulocyte	CAF_c1	Yes	No
LGALS9	HAVCR2	Granulocyte	CAF_c2	Yes	No
TNFRSF14	BTLA	Granulocyte	CAF_c3	Yes	No

Top thirty checkpoint interactions as inferred by iTALK between all three clusters of cancer associated fibroblasts (CAF_c1, CAF_c2, CAF_c3), CD8 T cells, macrophage, and granulocytes in the TGFβ-TRAP sample. Interactions between cancer associated fibroblasts clusters were excluded. Last column represents whether interaction was in the top thirty checkpoint interactions of the Control sample.

Supplementary Table 9. Top 30 Other Interactions in Control Sample

Top 30 Other Interactions in Control					
**ligand**	**receptor**	**cell_from**	**cell_to**	**Present in Control Top 30**	**Present in TGFβ-TRAP Top 30**
APP	CD74	CAF_c1	Macrophage	Yes	No
APP	CD74	Macrophage	Macrophage	Yes	No
APP	CD74	CAF_c1	Granulocyte	Yes	No
APP	LRP1	Macrophage	CAF_c2	Yes	No
APP	CD74	CAF_c2	Macrophage	Yes	No
APP	LRP1	Macrophage	CAF_c1	Yes	No
APP	CD74	Macrophage	Granulocyte	Yes	No
C3	ITGAM	CAF_c1	Macrophage	Yes	No
C3	LRP1	CAF_c1	Macrophage	Yes	No
COL1A1	CD44	CAF_c2	Macrophage	Yes	Yes
COL1A1	CD44	CAF_c1	Macrophage	Yes	No
COL1A1	CD44	CAF_c3	Macrophage	Yes	Yes
COL1A1	CD44	CAF_c2	Granulocyte	Yes	No
COL1A1	ITGAV	CAF_c2	Macrophage	Yes	No
COL1A2	CD44	CAF_c2	Macrophage	Yes	Yes
COL1A2	CD44	CAF_c1	Macrophage	Yes	Yes
COL3A1	ITGB1	CAF_c1	Macrophage	Yes	Yes
FN1	CD44	CAF_c1	Macrophage	Yes	Yes
FN1	CD44	CAF_c3	Macrophage	Yes	Yes
FN1	CD44	Macrophage	Macrophage	Yes	Yes
FN1	CD44	Macrophage	CAF_c1	Yes	Yes
FN1	CD44	CAF_c2	Macrophage	Yes	No
FN1	ITGB1	Macrophage	CAF_c3	Yes	Yes
FN1	ITGA9	CAF_c1	Macrophage	Yes	No
FN1	ITGA9	CAF_c3	Macrophage	Yes	No
PSAP	LRP1	Macrophage	CAF_c2	Yes	No
PSAP	LRP1	Macrophage	CAF_c1	Yes	No
TNC	ITGA9	CAF_c2	Macrophage	Yes	No
TNC	ITGAV	CAF_c2	Macrophage	Yes	No
TNC	ITGA9	CAF_c2	Granulocyte	Yes	No

Top thirty other interactions as inferred by iTALK between all three clusters of cancer associated fibroblasts (CAF_c1, CAF_c2, CAF_c3), CD8 T cells, macrophage, and granulocytes in the Control sample. Interactions between CAF clusters were excluded. Last column represents whether interaction was in the top thirty other interactions of the TGFβ-TRAP sample.

Supplementary Table 10. Top 30 Other Interactions in TGFβ-TRAP Sample

Top 30 Other Interactions in TGFB-TRAP					
**ligand**	**receptor**	**cell_from**	**cell_to**	**Present in TGFβ-TRAP Top 30**	**Present in Control Top 30**
APP	LRP1	CAF_c1	Macrophage	Yes	No
APP	LRP1	CAF_c3	Macrophage	Yes	No
COL1A1	CD44	CAF_c2	Macrophage	Yes	Yes
COL1A1	CD44	CAF_c3	Macrophage	Yes	Yes
COL1A2	CD44	CAF_c1	Macrophage	Yes	Yes
COL1A2	CD44	CAF_c2	Macrophage	Yes	Yes
COL1A2	ITGA1	CAF_c1	CD8	Yes	No
COL1A2	ITGA1	CAF_c2	CD8	Yes	No
COL3A1	ITGB1	CAF_c1	Macrophage	Yes	Yes
F13A1	ITGA4	Macrophage	CD8	Yes	No
FN1	ITGA4	Macrophage	CD8	Yes	No
FN1	CD44	Macrophage	CAF_c3	Yes	No
FN1	ITGA4	CAF_c3	CD8	Yes	No
FN1	ITGB1	Macrophage	CAF_c3	Yes	Yes
FN1	ITGA4	CAF_c1	CD8	Yes	No
FN1	CD44	Macrophage	CAF_c1	Yes	Yes
FN1	CD44	Macrophage	Macrophage	Yes	Yes
FN1	ITGA6	Macrophage	CAF_c3	Yes	No
FN1	ITGA9	Macrophage	CAF_c2	Yes	No
FN1	ITGA4	CAF_c2	CD8	Yes	No
FN1	SDC2	Macrophage	CAF_c3	Yes	No
FN1	ITGAV	Macrophage	CAF_c3	Yes	No
FN1	CD44	CAF_c3	Macrophage	Yes	Yes
FN1	ITGA4	Macrophage	Granulocyte	Yes	No
FN1	ITGA4	Macrophage	Macrophage	Yes	No
FN1	CD44	CAF_c1	Macrophage	Yes	Yes
FN1	SDC2	Macrophage	CAF_c1	Yes	No
FN1	ITGA4	CAF_c3	Granulocyte	Yes	No
FN1	ITGA4	CAF_c3	Macrophage	Yes	No
FN1	ITGAV	Macrophage	CAF_c1	Yes	No

Top thirty other interactions as inferred by iTALK between all three clusters of cancer associated fibroblasts (CAF_c1, CAF_c2, CAF_c3), CD8 T cells, macrophage, and granulocytes in the TGFβ-TRAP sample. Interactions between cancer associated fibroblasts clusters were excluded. Last column represents whether interaction was in the top thirty other interactions of the Control sample.

Supplementary Table 11. Top 30 Growth Factor Interactions in anti-PD-1 Sample

Top 30 Growth Factor Interactions in anti-PD-1					
**ligand**	**receptor**	**cell_from**	**cell_to**	**Present in anti-PD-1 Top 30**	**Present in Combo Top 30**
FGF13	EGFR	Granulocyte	CAF_c1	Yes	No
FGF13	EGFR	Macrophage	CAF_c1	Yes	Yes
FGF2	NRP1	CAF_c2	Macrophage	Yes	Yes
FGF2	CD44	CAF_c2	Macrophage	Yes	Yes
TGFB1	TGFBR3	Macrophage	CAF_c1	Yes	Yes
TGFB1	TGFBR3	Granulocyte	CAF_c1	Yes	Yes
TGFB1	TGFBR1	Macrophage	Macrophage	Yes	Yes
TGFB1	TGFBR1	Granulocyte	Macrophage	Yes	No
TGFB1	TGFBR1	Macrophage	Granulocyte	Yes	No
TGFB1	TGFBR1	Granulocyte	Granulocyte	Yes	No
TGFB1	TGFBR1	CAF_c2	Macrophage	Yes	No
TGFB1	TGFBR1	CAF_c2	Granulocyte	Yes	No
TGFB1	TGFBR1	CAF_c3	Macrophage	Yes	No
TGFB2	TGFBR3	Macrophage	CAF_c1	Yes	No
TGFB2	TGFBR1	Macrophage	Macrophage	Yes	No
TGFB2	TGFBR1	Macrophage	Granulocyte	Yes	No
TGFB2	TGFBR1	CAF_c1	Macrophage	Yes	No
TGFB2	TGFBR1	CAF_c1	Granulocyte	Yes	No
TGFB2	TGFBR2	Macrophage	CAF_c2	Yes	No
TGFB2	TGFBR1	Macrophage	CAF_c3	Yes	No
TGFB2	TGFBR1	Macrophage	CAF_c2	Yes	No
TGFB2	TGFBR3	Macrophage	CAF_c2	Yes	No
TGFB2	ACVR1	Macrophage	CAF_c3	Yes	No
TGFB2	TGFBR2	Macrophage	CAF_c1	Yes	No
TGFB2	TGFBR1	Macrophage	CAF_c1	Yes	No
TGFB2	ACVR1	Macrophage	CAF_c2	Yes	No
VEGFC	ITGA9	CAF_c3	Granulocyte	Yes	No
VEGFC	ITGA9	CAF_c3	Macrophage	Yes	No
VEGFC	ITGA9	CAF_c1	Granulocyte	Yes	Yes
VEGFC	ITGA9	CAF_c1	Macrophage	Yes	Yes

Top thirty growth factor interactions as inferred by iTALK between all three clusters of cancer associated fibroblasts (CAF_c1, CAF_c2, CAF_c3), CD8 T cells, macrophage, and granulocytes in the anti-PD-1 sample. Interactions between cancer associated fibroblasts clusters were excluded. Last column represents whether interaction was in the top thirty growth factor interactions of the Combo (anti-PD-1+ TGFβ-TRAP) sample.

Supplementary Table 12. Top 30 Growth Factor Interactions in Combo (anti-PD-1+ TGFβ-TRAP) Sample

Top 30 Growth Factor Interactions in Combo					
**ligand**	**receptor**	**cell_from**	**cell_to**	**Present in Combo Top 30**	**Present in anti-PD-1 Top 30**
FGF13	EGFR	Macrophage	CAF_c1	Yes	Yes
FGF2	NRP1	CAF_c1	Macrophage	Yes	No
FGF2	CD44	CAF_c1	Macrophage	Yes	No
FGF2	NRP1	CAF_c2	Macrophage	Yes	Yes
FGF2	CD44	CAF_c2	Macrophage	Yes	Yes
HBEGF	CD44	CAF_c3	Macrophage	Yes	No
HGF	SDC2	Macrophage	CAF_c3	Yes	No
IGFBP4	LRP6	CAF_c3	Macrophage	Yes	No
IGFBP4	LRP6	CAF_c2	Macrophage	Yes	No
TGFB1	TGFBR3	Macrophage	CAF_c1	Yes	Yes
TGFB1	TGFBR2	Macrophage	CAF_c2	Yes	No
TGFB1	SDC2	Macrophage	CAF_c3	Yes	No
TGFB1	TGFBR1	Macrophage	CAF_c3	Yes	No
TGFB1	TGFBR3	Granulocyte	CAF_c1	Yes	Yes
TGFB1	TGFBR1	Macrophage	Macrophage	Yes	Yes
TGFB1	TGFBR3	Macrophage	CAF_c2	Yes	No
TGFB1	ITGAV	Macrophage	CAF_c3	Yes	No
TGFB3	TGFBR1	CAF_c1	Macrophage	Yes	No
VEGFA	SIRPA	CAF_c3	Macrophage	Yes	No
VEGFA	ITGA9	CAF_c3	Macrophage	Yes	No
VEGFA	EGFR	Macrophage	CAF_c1	Yes	No
VEGFA	NRP1	CAF_c3	Macrophage	Yes	No
VEGFA	SIRPA	CAF_c3	Granulocyte	Yes	No
VEGFA	NRP2	CAF_c3	Macrophage	Yes	No
VEGFA	EGFR	Macrophage	CAF_c2	Yes	No
VEGFC	ITGA9	CAF_c1	Macrophage	Yes	Yes
VEGFC	NRP2	CAF_c1	Macrophage	Yes	No
VEGFC	ITGA9	CAF_c1	Granulocyte	Yes	Yes
VEGFC	NRP2	CAF_c1	Granulocyte	Yes	No
VEGFC	ITGB1	CAF_c1	Macrophage	Yes	No

Top thirty growth factor interactions as inferred by iTALK between all three clusters of cancer associated fibroblasts (CAF_c1, CAF_c2, CAF_c3), CD8 T cells, macrophage, and granulocytes in the Combo (anti-PD-1+ TGFβ-TRAP) sample. Interactions between cancer associated fibroblasts clusters were excluded. Last column represents whether interaction was in the top thirty growth factor interactions of the anti-PD-1 sample.

Supplementary Table 13. Top 30 Cytokine Interactions in anti-PD-1 Sample

Top 30 Cytokines Interactions in anti-PD-1					
**ligand**	**receptor**	**cell_from**	**cell_to**	**Present in anti-PD-1 Top 30**	**Present in Combo Top 30**
CCL13	CCR5	CAF_c2	Macrophage	Yes	No
CCL4	CCR5	CD8	Macrophage	Yes	Yes
CCL5	CCR5	Granulocyte	Macrophage	Yes	Yes
CCL5	CCR5	Granulocyte	Granulocyte	Yes	Yes
CCL5	CCR5	Macrophage	Macrophage	Yes	No
CCL5	CCR5	CAF_c2	Macrophage	Yes	No
CCL5	CCR5	Macrophage	Granulocyte	Yes	No
IL15	IL2RB	CAF_c3	CD8	Yes	Yes
IL15	IL2RB	Macrophage	CD8	Yes	Yes
IL15	IL15RA	CAF_c3	Macrophage	Yes	No
IL15	IL2RA	CAF_c3	CD8	Yes	No
IL16	CCR5	CAF_c1	Macrophage	Yes	Yes
IL16	CCR5	CAF_c1	Granulocyte	Yes	Yes
IL16	CCR5	CAF_c1	CD8	Yes	Yes
IL16	CCR5	CAF_c2	Macrophage	Yes	Yes
IL16	CCR5	CAF_c2	Granulocyte	Yes	No
IL18	IL1RAPL1	Macrophage	CAF_c1	Yes	No
IL1A	IL1R1	Macrophage	CAF_c1	Yes	Yes
IL1A	IL1R1	Granulocyte	CAF_c1	Yes	No
IL1A	IL1R1	Macrophage	CAF_c2	Yes	Yes
IL1B	IL1R1	Granulocyte	CAF_c1	Yes	Yes
IL1B	IL1R1	Macrophage	CAF_c1	Yes	Yes
IL1B	IL1R1	Granulocyte	CAF_c2	Yes	No
IL1B	IL1R1	Macrophage	CAF_c2	Yes	Yes
IL1RN	IL1R1	Granulocyte	CAF_c1	Yes	No
IL1RN	IL1R1	Macrophage	CAF_c1	Yes	No
IL34	CSF1R	CAF_c3	Macrophage	Yes	Yes
IL34	CSF1R	CAF_c3	Granulocyte	Yes	Yes
IL34	CSF1R	CAF_c1	Macrophage	Yes	No
IL34	CSF1R	CAF_c1	Granulocyte	Yes	No

Top thirty cytokine interactions as inferred by iTALK between all three clusters of cancer associated fibroblasts (CAF_c1, CAF_c2, CAF_c3), CD8 T cells, macrophage, and granulocytes in the anti-PD-1 sample. Interactions between cancer associated fibroblasts clusters were excluded. Last column represents whether interaction was in the top thirty cytokine interactions of the Combo (anti-PD-1+ TGFβ-TRAP) sample.

Supplementary Table 14. Top 30 Cytokine Interactions in Combo (anti-PD-1+ TGFβ-TRAP) Sample

Top 30 Cytokines Interactions in Combo					
**ligand**	**receptor**	**cell_from**	**cell_to**	**Present in Combo Top 30**	**Present in anti-PD-1 Top 30**
CCL4	CCR5	CD8	Macrophage	Yes	Yes
CCL5	CCR5	Granulocyte	CAF_c3	Yes	No
CCL5	CCR5	Granulocyte	Macrophage	Yes	Yes
CCL5	CCR5	Granulocyte	Granulocyte	Yes	Yes
CCL5	SDC4	Granulocyte	CAF_c1	Yes	No
CCL5	SDC4	Granulocyte	CAF_c3	Yes	No
CCL5	CCR5	CD8	Macrophage	Yes	No
CCL7	CCR5	CAF_c3	Macrophage	Yes	No
CCL7	CCR5	Macrophage	CAF_c3	Yes	No
CCL7	CCR5	CAF_c3	Granulocyte	Yes	No
CCL8	CCR5	Macrophage	CAF_c3	Yes	No
CCL8	CCR5	Macrophage	Macrophage	Yes	No
CCL8	CCR5	CAF_c3	Macrophage	Yes	No
CCL8	CCR5	Macrophage	Granulocyte	Yes	No
CCL8	CCR5	CAF_c3	Granulocyte	Yes	No
IL15	IL2RB	CAF_c3	CD8	Yes	Yes
IL15	IL2RB	Macrophage	CD8	Yes	Yes
IL16	CCR5	CAF_c1	Macrophage	Yes	Yes
IL16	CCR5	CAF_c1	Granulocyte	Yes	Yes
IL16	CCR5	CAF_c1	CD8	Yes	Yes
IL16	CCR5	CAF_c2	Macrophage	Yes	Yes
IL18	IL18R1	CAF_c3	CD8	Yes	No
IL1A	IL1R1	Macrophage	CAF_c1	Yes	Yes
IL1A	IL1R1	Macrophage	CAF_c2	Yes	Yes
IL1B	IL1R1	Macrophage	CAF_c1	Yes	Yes
IL1B	IL1R1	Macrophage	CAF_c2	Yes	Yes
IL1B	IL1R1	Granulocyte	CAF_c1	Yes	Yes
IL1B	IL1R1	Macrophage	CAF_c3	Yes	No
IL34	CSF1R	CAF_c3	Macrophage	Yes	Yes
IL34	CSF1R	CAF_c3	Granulocyte	Yes	Yes

Top thirty cytokine interactions as inferred by iTALK between all three clusters of cancer associated fibroblasts (CAF_c1, CAF_c2, CAF_c3), CD8 T cells, macrophage, and granulocytes in the Combo (anti-PD-1+ TGFβ-TRAP) sample. Interactions between cancer associated fibroblasts clusters were excluded. Last column represents whether interaction was in the top thirty cytokine interactions of the anti-PD-1 sample.

Supplementary Table 15. Top 30 Checkpoint Interactions in anti-PD-1 Sample

Top 30 Checkpoint Interactions in anti-PD-1					
**ligand**	**receptor**	**cell_from**	**cell_to**	**Present in anti-PD-1 Top 30**	**Present in Combo Top 30**
BTLA	CD247	CD8	CD8	Yes	Yes
CD274	PDCD1	Granulocyte	CD8	Yes	Yes
CD274	PDCD1	CAF_c3	CD8	Yes	Yes
CD274	PDCD1	Macrophage	CD8	Yes	Yes
CD274	PDCD1	Granulocyte	CAF_c3	Yes	No
CD274	PDCD1	CAF_c2	CD8	Yes	No
CD274	PDCD1	Macrophage	CAF_c3	Yes	No
CD80	CD28	Macrophage	CD8	Yes	No
CD80	CD28	Granulocyte	CD8	Yes	No
CD86	CD28	Granulocyte	CD8	Yes	Yes
CD86	CD28	Macrophage	CD8	Yes	Yes
CD86	CTLA4	Granulocyte	CAF_c2	Yes	No
CD86	CTLA4	Granulocyte	CD8	Yes	Yes
CD86	CTLA4	Macrophage	CAF_c2	Yes	No
CD86	CTLA4	Macrophage	CD8	Yes	Yes
CD86	CD28	CD8	CD8	Yes	No
CD86	CD28	CAF_c3	CD8	Yes	Yes
LGALS9	HAVCR2	Macrophage	CD8	Yes	Yes
LGALS9	HAVCR2	CAF_c3	CD8	Yes	Yes
LGALS9	HAVCR2	Macrophage	Granulocyte	Yes	No
LGALS9	HAVCR2	Macrophage	Macrophage	Yes	Yes
LGALS9	HAVCR2	CAF_c3	Granulocyte	Yes	Yes
LGALS9	HAVCR2	Macrophage	CAF_c3	Yes	Yes
LGALS9	HAVCR2	CAF_c2	CD8	Yes	Yes
LGALS9	HAVCR2	CAF_c3	Macrophage	Yes	Yes
LGALS9	HAVCR2	Granulocyte	CD8	Yes	Yes
LGALS9	HAVCR2	CAF_c1	CD8	Yes	Yes
LGALS9	HAVCR2	CD8	CD8	Yes	No
PDCD1LG2	PDCD1	Granulocyte	CD8	Yes	No
PDCD1LG2	PDCD1	Macrophage	CD8	Yes	Yes

Top thirty checkpoint interactions as inferred by iTALK between all three clusters of cancer associated fibroblasts (CAF_c1, CAF_c2, CAF_c3), CD8 T cells, macrophage, and granulocytes in the anti-PD-1 sample. Interactions between cancer associated fibroblasts clusters were excluded. Last column represents whether interaction was in the top thirty checkpoint interactions of the Combo (anti-PD-1+ TGFβ-TRAP) sample.

Supplementary Table 16. Top 30 Checkpoint Interactions in Combo (anti-PD-1+ TGFβ-TRAP) Sample

Top 30 Checkpoint Interactions in Combo					
**ligand**	**receptor**	**cell_from**	**cell_to**	**Present in Combo Top 30**	**Present in anti-PD-1 Top 30**
BTLA	CD247	CAF_c3	CD8	Yes	No
BTLA	CD247	CD8	CD8	Yes	Yes
BTLA	CD247	CAF_c1	CD8	Yes	No
CD274	PDCD1	CAF_c3	CD8	Yes	Yes
CD274	PDCD1	Macrophage	CD8	Yes	Yes
CD274	PDCD1	Granulocyte	CD8	Yes	Yes
CD80	CTLA4	Macrophage	CD8	Yes	No
CD80	CTLA4	Granulocyte	CD8	Yes	No
CD86	CTLA4	Macrophage	CD8	Yes	Yes
CD86	CD28	Macrophage	CD8	Yes	Yes
CD86	CD28	Macrophage	CAF_c3	Yes	No
CD86	CTLA4	CAF_c3	CD8	Yes	No
CD86	CTLA4	Granulocyte	CD8	Yes	Yes
CD86	CD28	CAF_c3	CD8	Yes	Yes
CD86	CD28	Granulocyte	CD8	Yes	Yes
CD86	CD28	Granulocyte	CAF_c3	Yes	No
CD86	CTLA4	CAF_c2	CD8	Yes	No
LGALS9	HAVCR2	CAF_c3	CD8	Yes	Yes
LGALS9	HAVCR2	CAF_c2	CD8	Yes	Yes
LGALS9	HAVCR2	Macrophage	CD8	Yes	Yes
LGALS9	HAVCR2	CAF_c3	Macrophage	Yes	Yes
LGALS9	HAVCR2	CAF_c1	CD8	Yes	Yes
LGALS9	HAVCR2	Macrophage	CAF_c3	Yes	Yes
LGALS9	HAVCR2	CAF_c3	Granulocyte	Yes	Yes
LGALS9	HAVCR2	CAF_c2	Macrophage	Yes	No
LGALS9	HAVCR2	Granulocyte	CD8	Yes	Yes
LGALS9	HAVCR2	Macrophage	Macrophage	Yes	Yes
LGALS9	HAVCR2	CAF_c1	Macrophage	Yes	No
LGALS9	HAVCR2	CAF_c2	Granulocyte	Yes	No
PDCD1LG2	PDCD1	Macrophage	CD8	Yes	Yes

Top thirty checkpoint interactions as inferred by iTALK between all three clusters of cancer associated fibroblasts (CAF_c1, CAF_c2, CAF_c3), CD8 T cells, macrophage, and granulocytes in the Combo (anti-PD-1+ TGFβ-TRAP) sample. Interactions between cancer associated fibroblasts clusters were excluded. Last column represents whether interaction was in the top thirty checkpoint interactions of the anti-PD-1 sample.

Supplementary Table 17. Top 30 Other Interactions in anti-PD-1 Sample

Top 30 Other Interactions in anti-PD-1					
**ligand**	**receptor**	**cell_from**	**cell_to**	**Present in anti-PD-1 Top 30**	**Present in Combo Top 30**
ADAM12	ITGA9	CAF_c3	Granulocyte	Yes	No
ADAM12	ITGA9	CAF_c3	Macrophage	Yes	No
APP	LRP1	Macrophage	CAF_c2	Yes	Yes
APP	CD74	CAF_c1	Macrophage	Yes	Yes
APP	LRP1	CAF_c1	Macrophage	Yes	No
APP	CD74	CAF_c3	Macrophage	Yes	No
APP	LRP1	CAF_c3	Macrophage	Yes	No
C3	ITGAM	CAF_c1	Macrophage	Yes	No
COL1A1	CD44	CAF_c2	Macrophage	Yes	Yes
COL1A2	CD44	CAF_c2	Macrophage	Yes	Yes
COL3A1	ITGB1	CAF_c2	Macrophage	Yes	Yes
COL5A2	ITGA1	CAF_c2	CD8	Yes	No
F13A1	ITGA4	Macrophage	CD8	Yes	Yes
F13A1	ITGA9	Macrophage	Granulocyte	Yes	No
F13A1	ITGA9	Macrophage	CAF_c2	Yes	No
FN1	ITGA4	CAF_c1	CD8	Yes	Yes
FN1	ITGA4	Macrophage	CD8	Yes	Yes
FN1	ITGA4	CAF_c2	CD8	Yes	Yes
FN1	ITGA9	CAF_c1	Granulocyte	Yes	No
FN1	ITGA4	CAF_c3	CD8	Yes	No
FN1	ITGA9	Macrophage	Granulocyte	Yes	No
FN1	ITGA9	Macrophage	CAF_c2	Yes	No
FN1	ITGA9	CAF_c1	Macrophage	Yes	Yes
FN1	CD44	Macrophage	CAF_c3	Yes	No
FN1	ITGB1	Macrophage	CAF_c3	Yes	No
SPP1	ITGA4	CAF_c3	CD8	Yes	Yes
TNC	ITGA9	CAF_c2	Granulocyte	Yes	No
TNC	ITGA9	CAF_c2	Macrophage	Yes	No
TNC	ITGAV	CAF_c2	Macrophage	Yes	No
TNC	ITGAV	CAF_c2	CD8	Yes	No

Top thirty other interactions as inferred by iTALK between all three clusters of cancer associated fibroblasts (CAF_c1, CAF_c2, CAF_c3), CD8 T cells, macrophage, and granulocytes in the anti-PD-1 sample. Interactions between cancer associated fibroblasts clusters were excluded. Last column represents whether interaction was in the top thirty other interactions of the Combo (anti-PD-1+ TGFβ-TRAP) sample.

Supplementary Table 18. Top 30 Other Interactions in Combo (anti-PD-1+TGFβ-TRAP) Sample

Top 30 Other Interactions in Combo					
**ligand**	**receptor**	**cell_from**	**cell_to**	**Present in Combo Top 30**	**Present in anti-PD-1 Top 30**
ADAM12	ITGA9	CAF_c1	Macrophage	Yes	No
ADAM12	ITGA9	CAF_c2	Macrophage	Yes	No
ADAM12	ITGA9	CAF_c1	Granulocyte	Yes	No
APP	LRP1	Macrophage	CAF_c2	Yes	Yes
APP	LRP1	Macrophage	CAF_c1	Yes	No
APP	CD74	CAF_c1	Macrophage	Yes	Yes
APP	CD74	Macrophage	Macrophage	Yes	No
C3	LRP1	CAF_c1	Macrophage	Yes	No
COL1A1	CD44	CAF_c2	Macrophage	Yes	Yes
COL1A1	ITGAV	CAF_c2	Macrophage	Yes	No
COL1A1	ITGA1	CAF_c2	CD8	Yes	No
COL1A1	ITGB1	CAF_c2	Macrophage	Yes	No
COL1A1	ITGAV	CAF_c2	CD8	Yes	No
COL1A1	ITGAV	CAF_c2	Granulocyte	Yes	No
COL1A1	ITGA5	CAF_c2	Macrophage	Yes	No
COL1A2	CD44	CAF_c2	Macrophage	Yes	Yes
COL1A2	ITGAV	CAF_c2	Macrophage	Yes	No
COL1A2	CD44	CAF_c1	Macrophage	Yes	No
COL1A2	ITGA1	CAF_c2	CD8	Yes	No
COL3A1	ITGB1	CAF_c2	Macrophage	Yes	Yes
F13A1	ITGA4	Macrophage	CD8	Yes	Yes
F13A1	ITGA9	Macrophage	Macrophage	Yes	No
FN1	ITGA4	CAF_c1	CD8	Yes	Yes
FN1	ITGA9	CAF_c1	Macrophage	Yes	Yes
FN1	ITGA4	CAF_c2	CD8	Yes	Yes
FN1	SDC2	Macrophage	CAF_c3	Yes	No
FN1	ITGA4	Macrophage	CD8	Yes	Yes
NCAM1	PTPRA	CAF_c1	Macrophage	Yes	No
PSAP	LRP1	Macrophage	CAF_c2	Yes	No
SPP1	ITGA4	CAF_c3	CD8	Yes	Yes

Top thirty other interactions as inferred by iTALK between all three clusters of cancer associated fibroblasts (CAF_c1, CAF_c2, CAF_c3), CD8 T cells, macrophage, and granulocytes in the Combo (anti-PD-1+ TGFβ-TRAP) sample. Interactions between cancer associated fibroblasts clusters were excluded. Last column represents whether interaction was in the top thirty other interactions of the anti-PD-1 sample.

Supplementary Table 19. Sample Quality Metrics Output from CellRanger for KPC-4545 and KPC-3403

	Sample/Treatment	Fraction Reads in Cell	Reads Mapped Confidently in Transcriptome
KPC-4545	Control (sn1_4)	47.1%	32.4%
	TGFβ-TRAP (sn2_4)	25.0%	12.1%
	anti-PD-1 (sn4_4)	32.3%	11.8%
	anti-PD-1 + TGFβ-TRAP (sn5_4)	29.0%	11.3%
KPC-3403	Control (sn1_4)	15.3%	4.9%
	TGFβ-TRAP (sn2_4)	27.8%	23.3%
	anti-PD-1 (sn4_4)	33.4%	23.9%
	anti-PD-1 + TGFβ-TRAP (sn5_4)	13.8%	5.7%
